# BHLS$_2$ upgrade: $\tau$ spectra, muon HVP and the [$\pi ^0,~\eta ,~{\eta ^\prime }$] system

**DOI:** 10.1140/epjc/s10052-022-10096-4

**Published:** 2022-02-28

**Authors:** M. Benayoun, L. DelBuono, F. Jegerlehner

**Affiliations:** 1 LPNHE des Universités Paris VI et Paris VII, IN2P3/CNRS, 75252 Paris, France; 2 Institut für Physik, Humboldt-Universität zu Berlin, Newtonstrasse 15, 12489 Berlin, Germany; 3 Deutsches Elektronen-Synchrotron (DESY), Platanenallee 6, 15738 Zeuthen, Germany

## Abstract

The generic hidden local symmetry (HLS) model has recently given rise to its $\hbox {BHLS}_2$ variant, defined by introducing symmetry breaking mostly in the vector meson sector; the central mechanism is a modification of the covariant derivative at the root of the HLS approach. However, the description of the $\tau$ dipion spectra, especially the Belle one, is not fully satisfactory, whereas the simultaneous dealing with its annihilation sector ($e^+ e^- \rightarrow \pi ^+ \pi ^-/\pi ^+ \pi ^-\pi ^0/ \pi ^0 \gamma /\eta \gamma /K^+ K^-/K_L K_S$) is optimum. We show that this issue is solved by means of an additional breaking term which also allows us to consistently include the mixing properties of the $[\pi ^0,\eta ,{\eta ^\prime }]$ system within this extended $\hbox {BHLS}_2$ ($\hbox {EBHLS}_2$) scope. This mechanism, an extension of the usual ’t Hooft determinant term, only affects the kinetic energy part of the $\hbox {BHLS}_2$ Lagrangian. One thus obtains a fair account for the $\tau$ dipion spectra which complements the fair account of the annihilation channels already reached. The Belle dipion spectrum is found to provide evidence in favor of a violation of the conserved vector current (CVC) in the $\tau$ lepton decay; this evidence is enforced by imposing the conditions $<0|J_\mu ^q |[q^\prime \overline{q^\prime }](p)>=ip_\mu f_q \delta _{q q^\prime }, \{ [q {\overline{q}}], q=u,d,s\}$ on $\hbox {EBHLS}_2$ axial current matrix elements. $\hbox {EBHLS}_2$ is found to recover the usual (completed) formulae for the [$\pi ^0,~\eta ,~{\eta ^\prime }$] mixing parameters, and the global fits return mixing parameter values in agreement with expectations and better uncertainties. Updating the muon hadronic vacuum polarization (HVP), one also argues that the strong tension between the KLOE and BaBar pion form factors imposes to provide two solutions, namely $a_\mu ^{HVP-LO}(\mathrm{KLOE})=687.48 \pm 2.93$ and $a_\mu ^{HVP-LO}(\mathrm{BaBar})=692.53 \pm 2.95$, in units of $10^{-10}$, rather than some combination of these. Taking into account common systematics, their differences from the experimental BNL-FNAL average value exhibit significance $> 5.4\sigma$ (KLOE) and $> 4.1\sigma$ (BaBar), with fit probabilities favoring the former.

## Introduction

The Standard Model provides the accepted framework which embodies the strong, electromagnetic and weak interactions; it accounts accurately for the observable values reported from low energies up to the highest ones reached at the Large Hadron Collider (LHC). A very few specific measurements look borderline enough, however, to raise a hint of a physics beyond the Standard Model. Among these, the very precisely measured muon anomalous magnetic moment $a_\mu$ plays a special role; it has generated – and still generates – an important experimental and theoretical activity related to its measurement by the E821 Experiment at Brookhaven National Laboratory (BN)L [1] $a_\mu (\mathrm{BNL})=[11659209.1 \pm 6.3]\times 10^{-10}$, for its latest update; this value is at variance with expectations by $3.5 \sigma$ to $4.5\sigma$, depending on the various predictions, essentially differing by their estimates of the leading-order hadronic vacuum polarization (HVP-LO) as reported in [2] and displayed therein in Fig. 44.

Actually, the significance just quoted refers to using HVP-LO evaluations derived by means of various dispersion relation (DR) methods. In contrast, the Lattice QCD (LQCD) Budapest-Marseille-Wuppertal (BMW) [3] Collaboration recently published an estimate for the HVP-LO [3] claiming 0.8% precision and very close to what is needed to match the BNL measurement; accordingly, the BMW HVP-LO is at variance by more than $2.6 \sigma$ with any of the reported DR evaluations of the HVP-LO.

The Muon $g-2$ experiment running at the Fermi National Accelerator Laboratory (FNAL) has very recently published its first results [4] and found them in excellent accord with the previous BNL measurement [1]; the consistency of these two measurements allowing for their combination, the experimental reference value becomes:

$$\begin{aligned} a_\mu (\mathrm{Exp})=[11659206.1 \pm 4.1]\times 10^{-10}. \end{aligned}$$

Compared to the BNL measurement, this weighted average provides a noticeably improved uncertainty (30% reduction) and a downward shift by $3 \times 10^{-10}$. This value still numerically favors the BMW estimate [3] for the HVP-LO over any of the DR ones.

Hence, the puzzle “DR versus data” may become^1^ “DR versus LQCD,” for which some different kind of physics beyond the Standard Model would have to be invoked. Indeed, if the HVP-LO as derived by DR methods provides a good global electroweak (EW) fit, the change suggested by the BMW evaluation severely impacts the goodness of the global EW fit [6], except if the changes can be localized at low enough energy – Reference [6] quotes 1.94 GeV, assuming the change in the cross sections to be a mere global rescaling.

As long as the missing piece may spread out across the whole non-perturbative region of QCD, which as shown by the KEDR data [7] does not extend much above $\simeq 2$ GeV, one has at hand a somewhat large lever arm. However, the recent KMPS study [8] has shown that the missing contribution to the HVP-LO should come from the energy region below $\simeq 0.7$ GeV to accommodate the global EW fit. This restricts the requested missing part of the annihilation cross section $\sigma (e^+e^- \rightarrow \mathrm{hadrons})$ to a very limited energy region widely explored by several independent groups having collected data using different detectors and colliders; this missing hadronic cross section piece is expected to contribute an additional $\delta a_\mu \simeq (15 \div 20) \times 10^{-10}$ to the muon HVP-LO, larger than the light-by-light (LbL) contribution to the HVP.

Thus, the energy region to scrutinize is located well inside the realm of the effective resonance Lagrangian approaches (RLA) which have extended the scope of the chiral perturbation theory (ChPT). The inclusion of resonances has given rise to the resonance chiral perturbation theory (R$\chi$PT) formulation and to the hidden local symmetry (HLS) model which have been proved equivalent [9, 10]. Alongside other processes, the HLS model [11] encompasses the non-anomalous annihilation channels $e^+e^- \rightarrow \pi ^+\pi ^-/K^+K^-/K_LK_S$; it can be complemented by its anomalous sector [12] which allows one to also cover the $e^+e^- \rightarrow \pi ^+\pi ^-\pi ^0/\pi ^0 \gamma /\eta \gamma$ annihilation channels. Because it deals with only the lowest-lying resonance nonet, the validity range of the HLS Lagrangian naturally extends up to the $\phi$ mass region and is thus quite appropriate to explore the faulty energy region in accordance with QCD which is at the root of the different RLA.

As the BMW evaluation of the muon HVP-LO questions the annihilation data in the energy region up to the $\phi$ meson mass, it is worth testing our understanding of its physics by means of such RLA, in particular the HLS model, which allows us to explore this region already well covered by a large number of data samples in all the significant channels, and thus shrink the window of possibilities to find a non-negligible missing $\delta a_\mu$.

As the original HLS model^2^ assumes U(3) symmetry in both the vector (V) and pseudoscalar (P or PS) sectors, it should obviously be complemented by symmetry breaking inputs in order to account for the rich amount of data samples it is supposed to cover. A first release named BHLS [14, 15], essentially based on the Bando-Kugo-Yamawak (BKY) breaking mechanism [16, 17], extended [18] to account for isospin breaking effects, was proven to perform satisfactorily; however, it exhibited some difficulty in managing the threshold and $\phi$ regions for the dipion and the 3 pion annihilation channels, respectively. In order to solve this issue, the breaking procedure was revisited in depth and gave rise to $\hbox {BHLS}_2$ under two variants [19], namely the Basic Solution (BS) and the reference solution (RS). Both $\hbox {BHLS}_2$ variants are derived by complementing the BKY breaking mechanisms at work in the $\mathcal{L}_A$ and $\mathcal{L}_V$ sectors of the non-anomalous HLS Lagrangian by additional breaking schemes affecting only the vector meson fields.

Regarding the vector sector of the $\hbox {BHLS}_2$ BS variant, the new breaking input – named covariant derivative (CD) breaking – turns out to perform the substitution^3^$V \Longrightarrow V + \delta V$ in the covariant derivative which is a fundamental ingredient of the HLS model; the aim of $\delta V$ is to break the $U(3)_V$ symmetry for the components along the basis matrices $T_0=I/\sqrt{6}$, $T_3$ and $T_8$ of the canonical Gell–Mann *U*(3) algebra. The $V \Longrightarrow V + \delta V$ rule naturally propagates to the anomalous sector – i.e. the *VVP* and *VPPP* Lagrangian pieces.^4^

Regarding the PS sector of the BS variant of $\hbox {BHLS}_2$: Besides the BKY breaking associated with the $\mathcal{L}_A$ part of the non-anomalous Lagrangian, the symmetry has been reduced by including the so-called ’t Hooft determinant term [20]; for our purpose, this turns out to add the singlet term $\lambda /2 \partial _\mu \eta _0 \partial ^\mu \eta _0$ to the kinetic energy of the PS fields.

However, if this canceled out the difficulties met at the dipion threshold and in the $\phi$ mass region, the account for the dipion spectra collected in the $\tau$ decay was not fully satisfactory, as can be seen in Table 3 of [19] (see also the discussion in Sect. 17 herein); a closer look indicated that it is the description of the high statistics Belle sample [21] which is faulty. This issue was circumvented by introducing an additional breaking mechanism (the primordial mixing) which defines the RS. The original aim of the present study was to reexamine the issue actually raised by the $\tau$ dipion spectra and to determine which kind of breaking could solve the $\tau$ problem met by the BS variant of $\hbox {BHLS}_2$. In order to motivate this new breaking scheme – a generalization of the usual ’t Hooft determinant term – Section 3 proceeds to a thorough study of the various dipion $\tau$ spectra and of their impact on the other channels involved in the $\hbox {BHLS}_2$ framework, especially on the pion form factor in the timelike and spacelike regions.

Once motivated, this extended breaking scheme is precisely defined and analyzed in Sects. 4 and . The purpose of Sect. 6 is to address the modifications generated in the non-anomalous $\hbox {BHLS}_2$ Lagrangian derived in [19] by this newly introduced kinetic breaking. Special emphasis is given to the pion form factor involved in the decay of the $\tau$ lepton $F_\pi ^\tau (s)$ compared to its partner in $e^+e^-$ annihilations $F_\pi ^e(s)$; it is shown that, while $F_\pi ^e(0)=1$ is still fulfilled,^5^ the CVC assumption is violated in the $\tau$ sector as $F_\pi ^\tau (0) \ne 1$. As the ALEPH [22] and Cleo [23] spectra easily accommodate a modeling with either of the $F_\pi ^\tau (0) \ne 1$ and $F_\pi ^\tau (0) = 1$ constraints, this result emphasizes the interest in having another $\tau$ dipion spectrum with statistics comparable to those of Belle [21] or larger.

However, allowing for a violation of CVC within $\hbox {BHLS}_2$ cannot be solely localized in the $\tau$ sector of the $\hbox {BHLS}_2$ Lagrangian, and it propagates to the anomalous Lagrangian pieces as noted in Sect. 7 and developed in the various appendices. Hence, the description of processes as important as the $e^+ e^- \rightarrow \pi ^+ \pi ^- \pi ^0/\pi ^0 \gamma /\eta \gamma$ annihilations is extensively modified and so should be tested versus data, as well as the $P \rightarrow \gamma \gamma$ decay modes and those involving ${\eta ^\prime }V \gamma$ couplings. Prior to this exercise, our set of reference data samples has to be updated to account for new data samples [24, 25] or updated ones [26, 27]. This is done in two steps. First, the purpose of Sect. 8 is to deal with the newly issued three-pion data sample collected by the BESIII Collaboration [24]. It is shown that the BESIII spectrum energies should be appropriately recalibrated to match the common energy scale of the other data samples included in our reference data set, especially in the $\omega$ and $\phi$ peak locations.

On the other hand, dealing with the dipion spectra is of course an important – and controversial – issue because of the long-standing discrepancy between some of the available high statistics data samples. Therefore, we take advantage of the newly published SND dipion spectrum [25] to revisit in Sect. 9 the consistency analysis of the different dipion samples to illustrate the full picture and motivate the way we deal with strong tensions when evaluating physics quantities of importance, especially the muon HVP-LO.

Having updated our reference set of data samples, in Sect. 10 we report on global fits performed under various conditions, updating the results derived with the BS and RS variants of the former version of $\hbox {BHLS}_2$ and those obtained using the extended formulation which is the subject of the present study; this extension will be named $\hbox {EBHLS}_2$ for clarity. Section 11 addresses a key topic of the broken HLS model within the $\hbox {EBHLS}_2$ context. Indeed, the question of supposedly uncontrolled uncertainties associated with using fit results based on an effective Lagrangian may cast some shadow on this kind of method. To definitively address this issue, the best is to quantify the effect by comparing the estimates for the muon HVP derived from $\hbox {EBHLS}_2$ with those derived using more traditional methods *under similar conditions*. Section 11.1 illustrates for the dipion contribution to the muon HVP that specific biases attributable to using $\hbox {EBHLS}_2$ are negligible compared to (i) the way the systematics, especially the normalization uncertainty of the various spectra, are dealt with by the various authors, and (ii) the sample content used to derive one’s estimates. These two major sources of uncertainty are, on the other hand, common to any of the reported evaluations.

With these conclusions at hand, our evaluations of the muon HVP-LO are derived. Our favored result which excludes from the fit the dipion spectra from KLOE08 [28] and BaBar [29, 30] is examined in Sect. 11.2; an alternative solution where all KLOE data samples are discarded in favor of the BaBar one is also presented in Sect. 11.5. The full HVP-LO is constructed (Sect. 11.4) and compared with the other currently reported evaluations in Sect. 11.6. Equipped with the kinetic breaking mechanism defined in Sect. 4.2, $\hbox {EBHLS}_2$ is well suited to address the mixing properties of the $[\pi ^0,\eta ,{\eta ^\prime }]$ system more precisely than was done with a similar – but much less sophisticated – modeling in [31]. The final aim is to rely on the results of the $\hbox {EBHLS}_2$ fit over the largest set of data samples ever used to derive the corresponding mixing parameter values with optimum accuracy.

The derivation of the axial currents is the subject of Sect. 13. Section 14 addresses the singlet-octet basis parameterization defined by Kaiser and Leutwyler [32–34]; it is shown that $\hbox {EBHLS}_2$ allows one to recover the expected extended ChPT relations. In Sect. 15, a similar exercise is performed within the quark flavor basis developed by Feldmann, Kroll, and Stech (FKS) in [35–37] and one also yields the expected results. This clearly represents a valuable piece of information about the dealing of $\hbox {EBHLS}_2$ in its PS sector.

The aim of Sects. 16 and 17 is to push $\hbox {EBHLS}_2$ a step further: we focus on how isospin symmetry breaking shows up in the axial currents $J_\mu ^q$ associated with light quark pairs $\{ [q {\overline{q}}], q=u,d,s\}$ when expressed in terms of PS bare fields – a leading-order approximation. The Kroll conditions [38]:

$$\begin{aligned} <0|J_\mu ^q |[q^\prime \overline{q\prime }](p)>=ip_\mu f_q \delta _{q q\prime } \end{aligned}$$

are then examined in detail and shown to exhibit – at $\mathcal{O}(\delta )$ in breakings – unexpected constraints among the various components of the kinetic breaking term. In particular, satisfying the Kroll conditions implies that a kinetic breaking with only a $\partial _\mu \eta _0 \partial ^\mu \eta _0$ term is not consistent and should be extended in order to involve $\partial \pi ^0,\partial \eta _0$ and $\partial \eta _8$ quadratic contributions. Whether this property is inherent to only $\hbox {EBHLS}_2$ looks unlikely.

In Sect. 18, we report on additional $\hbox {EBHLS}_2$ fits suggested by the Kroll conditions and tabulate the fit parameter values. The short Sect. 19 reports on side consequences on some physics parameters, especially the muon HVP-LO. Sections 20 and 21 report on the numerical evaluation of the $[\pi ^0,\eta ,{\eta ^\prime }]$ mixing parameters and compare this with available results from other groups.

Finally, Sect. 22 collects the conclusions of this work, an almost 100% COVID-19 lockdown work.

## Preamble: on the free parameters of the $\hbox {BHLS}_2$ model

Significant (anti-)correlations between $\Sigma _V$, $z_V$ and the specific HLS parameter *a* have been reported in our study [19]; this topic was the purpose of its Sect. 20.1. As parameter correlations may easily be of pure numerical origin,^6^ we did not go beyond analyzing the issue numerically but emphasized that the physics conclusions were safe, i.e. not shadowed by these correlations.

Actually, one can go a step further. Indeed, it can be remarked that the three parameters $\Sigma _V$, $z_V$ and *a* are involved only in the $\mathcal{L}_V$ piece of the non-anomalous $\hbox {BHLS}_2$ Lagrangian $\mathcal{L}_\mathrm{HLS}=\mathcal{L}_A + a\mathcal{L}_V$ and do not occur in its anomalous FKTUY pieces [12, 13]. Let us consider the pieces inherited from $a\mathcal{L}_V$ named here and in [19] $\mathcal{L}_\mathrm{VMD}$ and $\mathcal{L}_\tau$ and perform therein the following parameter redefinition:

1 $$\begin{aligned} \displaystyle a \longrightarrow a^\prime= & {} a(1+ \Sigma _V ) ,\nonumber \\ \displaystyle z_V \longrightarrow z_V^\prime= & {} z_V/(1+ \Sigma _V ) \simeq z_V (1- \Sigma _V ), \end{aligned}$$

where $\Sigma _V$ and $z_V$ are introduced by the $X_V$ breaking matrix affecting $\mathcal{L}_V$ which actually writes $X_V=\mathrm{diag} (1+\Sigma _V/2,1-\Sigma _V/2,\sqrt{z_V})$ in the $\hbox {BHLS}_2$ framework.^7^

One can then check that the dependency upon $\Sigma _V$ drops out everywhere except in the $W^\pm$ mass term shown in the $\mathcal{L}_\tau$ Lagrangian piece (see Eq. (40) below). Obviously, this mass term has no influence on the phenomenology we address and thus is discarded.

It follows from here that the *actual* values for *a*, $z_V$ and $\Sigma _V$ are in fact out of reach and that the single quantities which can be accessed using the data are their $a^\prime$, $z_V^\prime$ combinations. Practically, fitting within the $\hbox {BHLS}_2$ framework – having fixed $\Sigma _V=0$ – reduces the parameter freedom and the parameter correlations without any loss in the physics insight, being understood that the derived *a* and $z_V$ are nothing but $a^\prime$ and $z_V^\prime$ just defined.

On the other hand, specific parameters are involved in order to deal with the $[\pi ^0,~\eta ,~{\eta ^\prime }]$ system. They come from the transformation leading from the renormalized fields – those which diagonalize the PS kinetic energy term – to the physically observable $[\pi ^0,~\eta ,~{\eta ^\prime }]$ states and can be found^8^ in Sect. 5. These parameters have been named $\theta _P$, $\epsilon$ and $\epsilon ^\prime$ in accordance with the usual custom [35, 38].

In our previous works on the HLS model, in particular [14, 19], one of the ($\eta ,~{\eta ^\prime }$) mixing angles [32, 33] was constrained ($\theta _0 \equiv 0$) following an earlier study [31]. This turns out to impose the condition that the mixing angle $\theta _P$ be algebraically related to the BKY parameter $z_A$ and the nonet symmetry breaking parameter $\lambda$ (see Sect. 4.4 in [14]). The experimental picture having dramatically changed since [31], this assumption certainly deserve to be revisited, as will be done in the present work. Moreover, we also imposed [39]:

2 $$\begin{aligned}&\displaystyle \epsilon \epsilon ^\prime =-\epsilon _0^2 \sin {2 \theta _P},\quad \mathrm{with}\quad \displaystyle \epsilon _0 =\frac{\sqrt{3}}{4} ~\frac{m_d-m_u}{m_s-{\hat{m}}} \quad \mathrm{and}\nonumber \\&\quad {\hat{m}}=\frac{1}{2} (m_u+m_d). \end{aligned}$$

As a whole, this reduces the number of free parameters by two units without any degradation of the fit quality or any change in the HVP values.

However, for the present purpose, it has been found worthwhile to release these constraints and let $\theta _P$, $\epsilon$ and $\epsilon ^\prime$ vary freely. When analyzing below the $[\pi ^0,~\eta ,~{\eta ^\prime }]$ mixing properties, this assumption will be revisited in a wider context.

## Revisiting the $\tau$ dipion spectra: a puzzle?

Section 17 of [19] reported the properties of our set of – more than 50 – data samples when submitted to global fits based on either of the reference solution (RS) and basic solution (BS) variants of the $\hbox {BHLS}_2$ model. Table 3 therein displays a detailed account of the information returned by the fits for the various physics channels. More precisely, this table shows that the reported $\chi ^2/N_{points}$ averages for the displayed groups of the data samples held are generally of the order 1 – with the sole exception of the $K^+ K^-$ data sample from [40].

The $\tau$ channel $\chi ^2/N_\mathrm{points}$ overall piece of information displayed in this Table 3 covers a data group merging the samples provided by the Aleph [22], Cleo [23] and Belle [21] collaborations. One can read^9^ therein: $\chi ^2/N_\mathrm{points} = 92/85$ (RS variant) and $\chi ^2/N_\mathrm{points} = 98/85$ (BS variant). In the following, one may refer to these data samples as A, C and B, respectively.

However, this fair behavior of the $\tau$ channel data actually hides contrasted behaviors among the three samples gathered inside this group. This issue deserves reexamination^10^ within the $\hbox {BHLS}_2$ [19] context.

It was noted in Sect. 11 of [19] that the subtraction polynomials $P^\tau _\pi (s)$ and $P^e_\pi (s)$ of the $\pi ^\pm \pi ^0$ and $\pi ^+ \pi ^-$ pion loops, respectively, involved in the pion form factors are different, allowing this way for relative isospin symmetry breaking (IB) effects; more precisely, they are related by:

3 $$\begin{aligned} P^\tau _\pi (s) = P^e_\pi (s) +\delta P^\tau _\pi (s) , \end{aligned}$$

and the polynomial $\delta P^\tau _\pi (s)$ is also determined by fit.

Within the $\hbox {BHLS}_2$ context, $P^e_\pi (s)$, as any of the other loops involved, is a second-degree polynomial with floating parameters. However, in order to obtain good global fits when including the $\tau$ data – especially the Belle spectrum [21] – the degree of $\delta P^\tau _\pi (s)$ has been increased to the third degree in the BS variant^11^ of $\hbox {BHLS}_2$. Actually, this $\delta P^\tau _\pi (s)$ degree assumption is not harmless, as it corresponds to introducing a non-renormalizable counter term in the renormalized $\hbox {BHLS}_2$ Lagrangian. As A and C are well managed within the BHLS framework with a second-degree $\delta P^\tau _\pi (s)$, the issue raised by the Belle spectrum is thus worthy of being cautiously examined; this is the matter of the present section.

For the series of ($\hbox {BHLS}_2$) global fits presented in the present section, we have chosen to discard the data covering the $e^+ e^- \rightarrow \pi ^+ \pi ^- \pi ^0$ annihilation channel to shorten the fit code execution times. The lowest energy data point of the Cleo spectrum [23] is discarded as outlier; with this proviso, the three $\tau$ spectra [21–23] are fully addressed within our fits from threshold up to 1 GeV.

Formally, the differences between the dipion spectra in the $\tau$ decay and in the $e^+ e^-$ annihilation should solely follow from isospin symmetry breaking (IB) effects. Therefore, a real understanding of these supposes *a minima* a simultaneous dealing with the $e^+ e^- \rightarrow \pi ^+ \pi ^-$ annihilation channel and with the dipion spectra collected in the $\tau ^\pm \rightarrow \pi ^\pm \pi ^0 \nu _\tau$ decay. The annihilation data addressed in our fitting codes – CMD-2 [42–44], SND [45] KLOE [46, 47], BESIII [26], Cleo-c [48] – have been presented^12^ in detail in Sect. 13 of [19].

Actually, the BESIII Collaboration has recently published an erratum [27] to their [26] which essentially confirms the original spectrum but drastically reduces the statistical uncertainties. This will not be discussed at length and we only quote the $\chi ^2/N_\mathrm{points}$ evolution: running our standard $\hbox {BHLS}_2$ code with the *uncorrected* BESIII dipion spectrum [26], the various fits return $\chi ^2/N_\mathrm{points} \simeq 35/60$, whereas running it with the *corrected* data [27] yields $\chi ^2/N_\mathrm{points} \simeq 50/60$; this more realistic goodness of fit clearly indicates that the errors are indeed better understood, allowing the BESIII spectrum to really influence the physics results derived from fits.

We should also note that the two dipion spectra from KLOE08 [28] and BaBar [29, 30], exhibiting a poor consistency with all the ($> 50$) others, are discarded since the very beginning of the HLS modeling program [14, 15]. Finally, the SND dipion spectrum [25] measured over the $0.525<\sqrt{s}<0.883$ GeV energy interval will be analyzed separately below.

### Fitting the $\tau$ dipion spectra

The $\tau$ spectra submitted to global fits are defined by:

4 $$\begin{aligned} \displaystyle \frac{1}{\Gamma _\tau } \frac{\mathrm{d} \Gamma _{\pi \pi }}{\mathrm{d}s}= \mathcal{B}_{\pi \pi } \frac{1}{N}\frac{\mathrm{d} N(s)}{\mathrm{d}s} \end{aligned}$$

using the event distributions and branching fractions $\mathcal{B}_{\pi \pi }$ provided by each of the Aleph [22], Belle [21] and Cleo [23] collaborations. The full $\tau$ width is derived from its lifetime taken from [49]. The relation with the pion form factor is:

5 $$\begin{aligned} \displaystyle \frac{\mathrm{d} \Gamma _{\pi \pi }}{\mathrm{d}s}= \frac{G_F^2}{m_\tau ^3} [S_\mathrm{EW} G_\mathrm{EM}(s)] |F_\pi ^\tau (s)|^2 \end{aligned}$$

where $F_\pi ^\tau (s)$ is derived from the $\hbox {BHLS}_2$ Lagrangian [19] and $S_\mathrm{EW}$ collects the short-range radiative corrections [50]; the long-range radiative corrections are collected in $G_\mathrm{EM}(s)$ and evaluated on the basis of [51–53]. The normalization of the full form factor at the origin is, thus, given by the product $[S_\mathrm{EW} G_\mathrm{EM}(s)]$ in the standard $\hbox {BHLS}_2$, which automatically fulfills^13^$F_\pi ^\tau (0)=1 +\mathcal{O}(\delta ^2)$.

**Table 1:** Global fit properties (spacelike data excluded): $\chi ^2$ values for the various sample groups; the numbers of data points are given between parentheses. The subtraction polynomial $\delta P^\tau (s)$ is always second degree except when explicitly stated (second data column). The tag “spectra” stands for fitting with the reported A, B and C dipion spectra; the tag “lineshapes” stands for the case when these spectra are normalized to their integral over the fitted energy range; the tag “rescaled” covers the case when a common rescaling factor is applied to the three dipion $\tau$ spectra. The last data column displays the results obtained fitting within $\hbox {EBHLS}_2$

$\hbox {BHLS}_2$ fit (excl. spacelike)	$\hbox {BHLS}_2$ $\equiv$ ($\lambda _3=0$)				$\lambda _3$ free spectra
	Spectra	Spectra (3rd deg.)	Lineshapes	Rescaled	
NSK $\pi ^+\pi ^-$ (127)	138	136	135	138	138
KLOE $\pi ^+\pi ^-$ (135)	146	143	145	144	140
BESIII $\pi ^+\pi ^-$ (60)	47	47	48	48	50
$\tau$ (ABC) (84)	122	92	79	79	78
$\tau$ (ALEPH) (37)	41	22	23	23	21
$\tau$ (CLEO) (28)	33	34	30	31	32
$\tau$ (Belle) (19)	48	36	26	25	25
Fit Prob.	66%	93%	95%	94%	96%

Anticipating the following sections, let us state that we will define an extension of standard $\hbox {BHLS}_2$ [19] to allow for a violation of CVC in the $\tau$ sector; it will be named $\hbox {EBHLS}_2$. The main difference – not the only one – between $\hbox {EBHLS}_2$ and the standard $\hbox {BHLS}_2$ [19] is that it fulfills $F_\pi ^\tau (0)=1-\lambda _3^2/2$ where $\lambda _3$ is a floating parameter of order $\mathcal{O}(\sqrt{\delta })$ reflecting a symmetry breaking. Moreover, setting $\lambda _3=0$ therein allows us to recover exactly the standard $\hbox {BHLS}_2$ [19]. When $\lambda _3 \ne 0$, the rescaling generated by $\hbox {EBHLS}_2$ is numerically modulated by accompanying changes in the internal structure of the $\rho$ term in $F_\pi ^\tau (s)$. On the other hand, as the rest of the non-anomalous Lagrangian pieces are unchanged, the properties of $F_\pi ^e(s)$ remain unchanged; in particular, the condition $F_\pi ^e(0)=1 +\mathcal{O}(\delta ^2)$ is still valid as in $\hbox {BHLS}_2$ because the term of $\mathcal{O}(\delta )$ vanishes by having stated [19] $\xi _0=\xi _8$.

As the $\tau$ data analysis is the main motivation for the forthcoming $\hbox {EBHLS}_2$ extension, it was found worthwhile to display, besides the standard $\hbox {BHLS}_2$ fit results, the corresponding $\hbox {EBHLS}_2$ information, prior to dealing with its derivation.^14^

### $\hbox {BHLS}_2$ global fits excluding the spacelike data

Table 1 reports on a series of fits aimed at coping with the $\tau$ topic; the global fits reported in this subsection discard the spacelike data [54, 55], and the discussion emphasizes solely the behavior of the dipion data from the annihilation channel and from the $\tau$ decay.

**Fig. 1:**
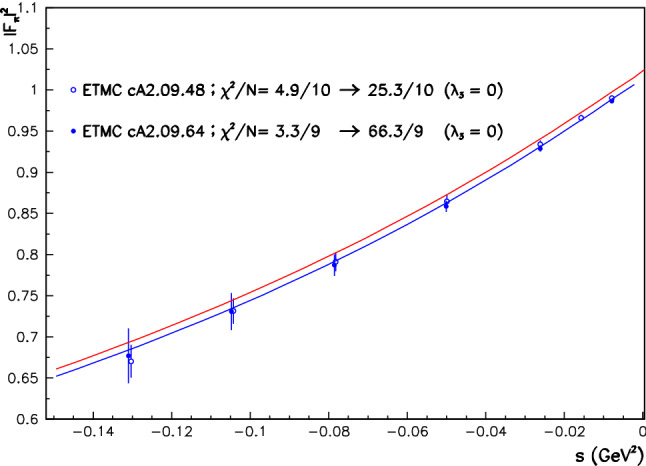
$|F_\pi (s)|^2$ derived from the fits with $\lambda _3=0$ (red curve) and with $\lambda _3 \ne 0$ (blue curve). The LQCD data points from [56], not fitted, are superimposed. The values for average $\chi ^2$ distances $\chi ^2/N_\mathrm{points}$ are shown for $\lambda _3 \ne 0$ and $\lambda _3 =0)$

As stated just above, the reference therein to the parameter $\lambda _3$ anticipates the $\hbox {EBHLS}_2$ extension proposed below, and states that the condition $\lambda _3=0$ is strictly identical to having $\hbox {BHLS}_2$ running, in particular its BS variant [19] solely used all along the present work, except as otherwise stated (Fig. 1).

**Fig. 2:**
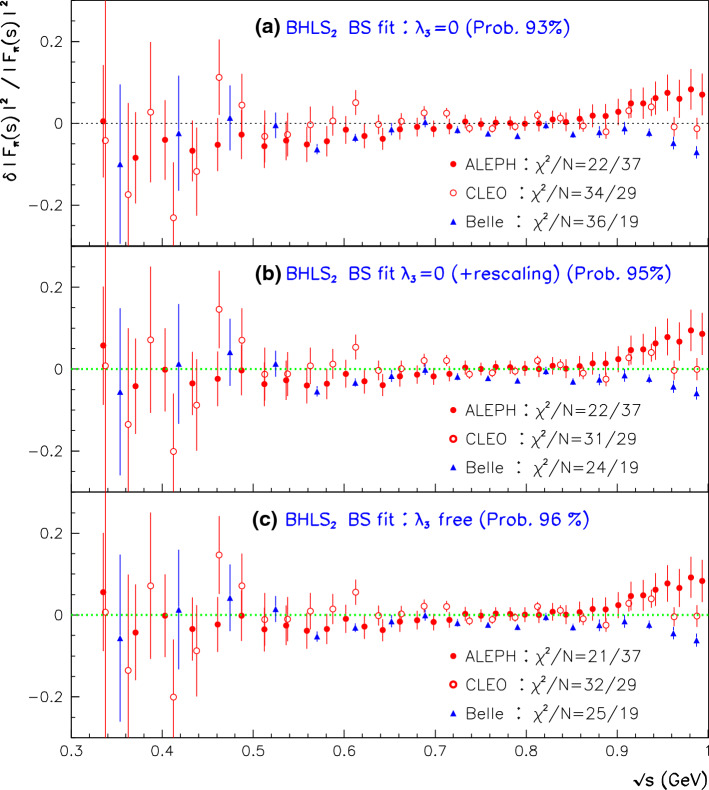
Normalized residuals derived in the global fits excluding the spacelike data. Top panel displays the case when A, B and C *as such* are simultaneously fitted; the middle panel displays the case where a common rescaling factor is applied to A, B and C. The bottom panel reports the corresponding results derived by fitting A, B and C *as such* within the $\hbox {EBHLS}_2$ framework

- The first data column displays the global fit performed using the published A, B and C spectra imposing the polynomial $\delta P^\tau _\pi (s)$ (see Eq. (3)) to be second degree. Obviously, the $\chi ^2/N_\mathrm{points}$ averages are reasonable for all the displayed data samples or groups shown (as well as those not shown) except for Belle, which yields the unacceptable average $\chi ^2/N_\mathrm{points} =2.52$.
- The simplest (ad hoc) choice to better accommodate the Belle spectrum turns out to allow $\delta P^\tau _\pi (s)$ to be third degree. Doing this, the second data column shows that the fit returns a fair probability, as already known since [19]. Indeed, besides a quite marginal improvement of the $e^+ e^- \rightarrow \pi ^+\pi ^-$ account, one observes a sensible improvement in the description of the Aleph ($\chi ^2:41 \rightarrow 22$) and Belle ($\chi ^2:48 \rightarrow 36$) spectra, whereas the Cleo spectrum $\chi ^2$ remains unchanged and satisfactory. The improvement for the Belle spectrum is significant ($\chi ^2/N_\mathrm{points}: 2.52 \rightarrow 1.90$) but not fully satisfactory. Nevertheless, the top panel in Fig. 2 shows that the normalized residuals for the $\tau$ spectra exhibit a reasonably flat behavior, thanks to having a third-degree $\delta P^\tau _\pi (s)$.
  So, once the degree for $\delta P^\tau _\pi (s)$ is appropriate, $\hbox {BHLS}_2$ gets a fair account for the A and C data and an acceptable one for the Belle spectrum.

However, inspired by the fit summary Table VII in the Belle paper [21], we have addressed two other similar strategies:

- Instead of fitting the A, B and C spectra *as such*, we choose to use each of them normalized to its integral over the fitting energy range ($<1.$ GeV); i.e we rather fit the A, B, C *lineshapes* within the global $\hbox {BHLS}_2$ framework. In this case, a second-degree $\delta P^\tau _\pi (s)$ is already sufficient and yields a fair global fit (95% probability). The corresponding results are displayed in the third data column; they are clearly satisfactory in both the annihilation channel and the $\tau$ sector. In this configuration, the Belle spectrum undergoes an individual $\chi ^2$ improvement by 10 units and comes out with a more reasonable $\chi ^2/N_\mathrm{points}= 1.37$.

Stated otherwise, once the $\tau$ spectra are normalized, $\hbox {BHLS}_2$ provides a fairly good simultaneous account of the A, B and C spectra and proves that the A, B and C *lineshapes* are quite consistent with each other without any need for some ad hoc trick. Moreover, there is no point in going beyond the second degree for $\delta P^\tau _\pi (s)$.

An obviously similar approach to the *lineshape* method just emphasized is to let the pion form factor $|F_\pi ^\tau (s)|^2$ be such that^15^$|F_\pi ^\tau (s=0)|^2 \ne 1$. This is inspired by the stand-alone fit performed by Belle and reported in Table VII of their [21].

In this study, a first fit has been performed across the full energy range of the Belle dipion spectrum using a Gounaris–Sakurai (GS) pion form factor $|F_\pi ^\tau (s)|^2$ – which fulfills $|F_\pi ^\tau (s=0)|^2 =1$; this fit is the matter of the leftmost data column of their Table VII and reports $\chi ^2/N_\mathrm{points}=80/62$. Belle also reports therein a second fit, having allowed for a mere rescaling

$$\begin{aligned} |F_\pi ^\tau (s)|^2 \rightarrow (1 + \lambda _\tau )|F_\pi ^\tau (s)|^2 \end{aligned}$$

of their GS parameterization; the corresponding results are reported in the rightmost data column of their Table VII with $\chi ^2/N_\mathrm{points}=65/62$. The noticeable 15-unit gain for the $\chi ^2$ value (a 4 $\sigma$ effect), resulting from a single additional floating parameter, stresses the relevance of what was just named $\lambda _\tau$. Let us perform likewise within the global $\hbox {BHLS}_2$ context.

- As the standard $\hbox {BHLS}_2$ [19] provides $|F_\pi ^\tau (s=0)|^2 = 1$, one performs as Belle using rescaling factors of the form $1+\lambda _\tau$. We have first performed global fits using a single $\tau$ spectrum (A, B, C in turn) and derived the following results:
  6 $$\begin{aligned} \left\{ \begin{array}{llll} \mathrm{Aleph:} &{} \lambda _\tau = (-7.63 \pm 0.66) \%, \\ &{} \chi ^2/N_\mathrm{points}=15/37, &{} \mathrm{Prob=} ~98 \%\\ \mathrm{Cleo:} &{} \lambda _\tau = (-3.12 \pm 0.66) \% , \\ &{} \chi ^2/N_\mathrm{points}=27/28,&{} \mathrm{Prob=} ~95 \%\\ \mathrm{Belle:} &{} \lambda _\tau = (-5.96 \pm 0.52) \% , \\ &{}\chi ^2/N_\mathrm{points}=23/19,&{} \mathrm{Prob=} ~92 \% \end{array} \right. \end{aligned}$$
  which, unexpectedly, indicate that B, as well as A and C, nicely accommodate a rescaling factor without degrading the description of the annihilation data. We also performed a global fit merging the three $\tau$ spectra each renormalized by a common (single) scale factor. We get:
  7 $$\begin{aligned}&\mathrm{A+B+C:}\;~\lambda _\tau = (-6.95 \pm 0.37) \% ,\nonumber \\&\chi ^2/N_\mathrm{points}=79/84, \mathrm{Prob} = 94\%; \end{aligned}$$
  more details can be found in the fourth data column of Table 1; the normalized residuals derived from this fit are displayed in the middle panel of Fig. 2. Comparing the fit results reported here, one observes a 13-unit gain compared to using a third-degree $\delta P^\tau (s)$, in line with the Belle fits – with exactly the same model parameter freedom as $\lambda _\tau$ comes replacing the coefficient dropped by reducing the degree of $\delta P^\tau (s)$ by one unit.

Such a *common* rescaling is certainly beyond experimental biases as, moreover, A, B and C have been collected with different detectors by independent teams. This is also much beyond the reported uncertainties on their respective $\tau ^- \rightarrow \pi ^- \pi ^0 \nu$ branching fractions which govern the absolute scale of the fitted $\tau$ spectra.

So, one reaches an outstanding fit quality by rescaling the three spectra *by the same amount*; this improvement of the $\tau$ sector is *not* obtained at the expense of degrading, even marginally, the account of the $e^+e^-$ annihilation data. Within the $\hbox {BHLS}_2$ context – and discarding the spacelike spectra – it is found that $\lambda _\tau = (-6.95 \pm 0.37) \%$, a non-negligible value. This amount is certainly related to intrinsic details of the Lagrangian model, noticeably the mass and width differences of the neutral and charged $\rho$ mesons which also contribute to the absolute scale.

Finally, the last data column in Table 1 shows that the forthcoming $\hbox {EBHLS}_2$ succeeds in producing a nice fit, very close to $\hbox {BHLS}_2$ ones corresponding to the information displayed by Eq. (7) and reported in the fourth data column of Table 1. The normalized residuals are displayed in the bottom panel of Fig. 2.

One can observe that the three sets of normalized residuals shown in Fig. 2 are almost identical and each is quite acceptable. Finally, we should stress that in all configurations, Table 1 exhibits a fair account of the A and C spectra; it is therefore noticeable, and even amazing, that a remarkable simultaneous fit of A, C and B can *also* be derived. Moreover, in both kinds of configurations (rescaling or not), the same, fair account of all the annihilation data is obtained.

### $\hbox {BHLS}_2$ global fits including the spacelike data

The fit properties and parameter values reported just above have been derived using the dipion spectra only in the timelike region for both the $\pi ^+ \pi ^-$ and $\pi ^\pm \pi ^0$ pairs. Moreover, it has been shown [19] that, within the $\hbox {BHLS}_2$ framework, the *same* analytic function describes fairly well the pion form factor in the spacelike *and* timelike energy regions. It is, therefore, desirable to enforce the impact of the analyticity requirement within the BHLS framework by requiring a simultaneous account of both energy regions.

**Table 2:** Global fit properties (including the spacelike data): $\chi ^2$ of the various sample groups; their numbers of data points are shown between parentheses. The subtraction polynomial $\delta P^\tau (s)$ is always second degree except when explicitly stated (second data column). The tag “spectra” stands for fitting the A, B and C $\tau$ dipion spectra as such; the tag “rescaled” covers the case when a common rescaling factor is applied to the three dipion $\tau$ spectra

Fitting framework (incl. spacelike)	$\hbox {BHLS}_2$ $\equiv$ ($\lambda _3=0$)			$\hbox {EBHLS}_2$ $\equiv$ ($\lambda _3$ free)	
	Spectra	Spectra (3rd deg.)	Rescaled (2nd deg.)	Spectra	Rescaled
NSK $\pi ^+\pi ^-$ (127)	138	134	137	138	136
KLOE $\pi ^+\pi ^-$ (135)	139	146	154	140	141
BESIII $\pi ^+\pi ^-$ (60)	48	47	47	48	48
Spacelike $\pi ^+\pi ^-$ (59)	62	65	77	62	61
$\tau$ (ABC) (84)	${ \times }$	92	88	81	77
$\tau$ (ALEPH) (37)	24	23	28	25	21
$\tau$ (CLEO) (28)	30	33	30	31	32
$\tau$ (Belle) (19)	${ \times }$	37	30	25	24
Fit Prob.	93%	89%	79%	91%	94%

Therefore, including the spacelike data [54, 55] in the set of samples submitted to the global fit appears a natural step. A priori, this should mostly affect $F_\pi ^e(s)$; however, as $F_\pi ^e(s)$ and $F_\pi ^\tau (s)$ are deeply interconnected within the $\hbox {BHLS}_2$ framework, extending the fit to the spacelike region can be of consequence for both form factors. This subsection reports on the global fit results derived when also including the spacelike data.

The first data column of Table 2 displays the fit information when fitting with $\hbox {BHLS}_2$ using a second-degree $\delta P^\tau (s)$ polynomial, *having discarded * the Belle spectrum. As evidenced by its reported probability (93%), the fit exhibits a nice account of each group of data samples as we always observe $\chi ^2/N_\mathrm{points} \simeq 1$. This proves that the need for a third-degree $\delta P^\tau (s)$ is caused solely by the Belle (B) spectrum.

The second data column in this table reports the same fit also including the Belle spectrum but with a third-degree $\delta P^\tau (s)$; it exactly corresponds to those already reported in the second data column of Table 1. The average $\chi ^2$ of the various sample groups are observed to be quite similar, including for the Belle data sample ($\chi ^2/N_\mathrm{points}=1.95$); for the spacelike data, it yields a favorable $\chi ^2/N_\mathrm{points}=1.10$. This case corresponds to the fit configurations previously used in [19]; with 89% probability, it is clearly a satisfactory solution, and one does not observe any degradation of the goodness of fit by having included the spacelike data.

The fit reported in the third data column of Table 2 is the exact analog of the one displayed in the fourth data column of Table 1, also taking into account the spacelike data. Here, also, $\delta P^\tau (s)$ carries the second degree. The best fit returns a global rescaling factor $1+\lambda _\tau$ with:

8 $$\begin{aligned}&\mathrm{A+B+C:}\,\, \lambda _\tau = (-4.42 \pm 0.40) \% , \nonumber \\&\chi ^2/N_\mathrm{points}=88/84,\mathrm{Prob} = 79\%. \end{aligned}$$

Comparing with Eq. (7), one observes a drop in probability produced by the inclusion of the spacelike data within the global fit procedure ($94\% \rightarrow 79\%$). Compared to having excluded the spacelike data, the effect is noticeable on the KLOE data ($\chi ^2/N_\mathrm{points}: 1.07 \rightarrow 1.14$) and for the $\tau$ data ($\chi ^2/N_\mathrm{points}: 0.94 \rightarrow 1.05$). On the other hand, having reduced the $\delta P^\tau (s)$ degree, one observes a clear degradation of the spacelike data account as $\chi ^2/N_\mathrm{points}: 1.10 \rightarrow 1.31$. Nevertheless, even if not the best reachable fit, as will be seen shortly, this configuration provides a quite reasonable picture.

The last two data columns in Table 2 refer to the extended $\hbox {BHLS}_2$ model fit results. In this case, we have examined the $\hbox {EBHLS}_2$ solution (fourth data column) and, for completeness, also performed the analysis with an additional rescaling of the $\tau$ spectra (fifth data column).

One clearly observes that the original spectra exhibit uniformly good properties in all channels, and the fit yields a 91% probability, as displayed in the fourth data column. Complemented with an additional common rescaling of the $\tau$ spectra, some marginal improvement is observed in the description of these as shown in the last data column of Table 2.

In contrast to the preceding subsection (no dealing with the spacelike data), using a second-degree $\delta P^\tau (s)$ and rescaling the $\tau$ spectra, while improving the $\tau$ sector, leads to a degraded account of the spacelike data ($\chi ^2/N_\mathrm{points}: 1.10 \rightarrow 1.31$) and a loss of the remarkable *prediction* of the LQCD pion form factor data [56] reported in [19]; this is illustrated by Fig. 1. Comparing this Figure with Fig. 8 of [19], derived by excluding the $\tau$ data from the fit, one observes good agreement with the $\hbox {EBHLS}_2(\lambda _3 \ne 0)$ solution.^16^ Finally, the last two data columns in Table 2 show that $\hbox {EBHLS}_2$ perfectly succeeds in recovering uniformly good $\chi ^2$’s with fit probabilities exceeding 90% and a fair account of all channels.

### Summary

Therefore, after including the $\tau$ data within the $\hbox {BHLS}_2$ minimization procedure, a fit using a third-degree $\delta P^\tau (s)$ succeeds, recalling the conclusions already reached in [19]; however, one has to accept an average $\chi ^2$ of 1.95 for Belle, whereas those for Aleph and Cleo are 0.62 and 1.18, respectively.

The fact that A, B and C carry a common lineshape may look hardly accidental. However, in contrast to Table 1, where evidence for solely a rescaling looks reasonable, Table 2 indicates that a mere rescaling is insufficient – not to mention that the $\hbox {EBHLS}_2$ prediction for the LQCD pion form factor data [56] is severely degraded compared to [19]. In this case, $\hbox {EBHLS}_2$ – complemented or not with a common rescaling of the $\tau$ spectra – performs nicely and fulfills all desirable analyticity requirements using a second-degree $\delta P^\tau (s)$ only, as should be preferred. The fits reported in Sects. 3.2 and 3.3 convincingly illustrate that the third degree looks somewhat ad hoc and can be avoided.

Therefore, we are led to complement the covariant derivative (CD) breaking introduced in [19] by an additional term (see Sect. 4) which breaks the kinetic sector of the HLS Lagrangian. This points toward a violation of CVC in the $\tau$ sector (at a $\simeq -2.5\%$ level for $|F_\pi ^\tau (s)|$). For completeness, we also examine the effect of a possible rescaling complementing the CVC breaking (generated by a nonzero $\lambda _3$); this is reported in the last data column of Table 2. This rescaling may correspond to a (higher order?) correction to the product $S_\mathrm{EW} G_\mathrm{EM}(s)$ (at a $\simeq -2\%$ level). However, this additional freedom does not produce a significant effect and is discarded from now on.

To conclude, the present analysis highlights the importance of having at one’s disposal a new high statistics $\tau$ dipion spectrum; it is, indeed, of importance to reach conclusions about the specific behavior exhibited by the Belle spectrum within global fits. The challenging properties exhibited by fitting the $\tau$ dipion spectra or, alternatively, their lineshapes may look amazing enough to call for a confirmation of the Belle spectrum properties. Indeed, it is not unlikely that the much higher statistics of the Belle spectrum (50 times those of Cleo [21]) allow a finer structure to show up, calling for a more refined description.

## Extending the $\hbox {BHLS}_2$ breaking scheme

The hidden local symmetry model [13] has been supplied with specific symmetry breaking mechanisms to provide the $\hbox {BHLS}_2$ framework [19] within which almost all $e^+e^-$ annihilation channels occurring up to the $\phi$ mass are encompassed. This allowed for a simultaneous fit of almost all collected data samples covering the non-anomalous decay channels ($\pi ^+ \pi ^-$, $K^+ K^-$, $K_L K_S$) and anomalous ones [12] ($\pi ^+ \pi ^- \pi ^0$, $\pi ^0 \gamma$, $\eta \gamma$). As clear from [19], one gets a fair description with superb goodness of fit.

As just emphasized in Sect. 3, the decay mode $\tau ^- \rightarrow \pi ^- \pi ^0 \nu _\tau$ is also a natural part of the successive HLS frameworks [14, 19, 41, 57, 58]. However, as illustrated by Table 2, the issue is whether one must consider, besides the ALEPH [22] and Cleo [23] spectra, those from Belle [21]. Our present goal is to define an extension of $\hbox {BHLS}_2$ which can naturally simultaneously encompass the A, C and B spectra and continuously recover the $\hbox {BHLS}_2$ framework in some smooth limit. Anticipatively, this extension has been named $\hbox {EBHLS}_2$.

$\hbox {BHLS}_2$ has been constructed by considering, besides the BKY mechanism [16, 17], the covariant derivative breaking and the primordial mixing procedures – see Sects. 4 and 8 of [19]. These essentially address the vector sector of the HLS model and rotations allow us to render the $\hbox {BHLS}_2$ Lagrangian canonical. This also lets the vector meson kinetic energy supplied by the Yang–Mills Lagrangian be canonical.

Regarding the pseudoscalar (PS) sector, the BKY mechanism [14, 16, 17] also contributes to break the symmetries of the HLS model. We should also emphasize that the mass breaking in the kaon sector is at the origin of the dynamical mixing of the vector mesons [57], which is the central piece of the various broken versions of the HLS model. Indeed, thanks to having different charged and neutral kaon loops, the ($\rho ^0$, $\omega$, $\phi$) mass matrix at one loop becomes non-diagonal and, thus, imposes another step in the vector field redefinition [14, 19]. This back-and-forth play between vector field redefinition and isospin symmetry breaking in the PS sector should be noted.

Besides the two mechanisms just listed, in order to account for the physics of the anomalous processes, the ’t Hooft determinant terms [20], more precisely its kinetic part, provide the needed nonet symmetry breaking in the PS sector. Moreover, higher-order and loop terms in chiral perturbation theory and QED corrections are expected to extend the breaking of the PS kinetic energy term beyond the singlet component. It is the purpose of the present section to extend the kinetic breaking^17^ to the full $(\pi ^0-\eta -{\eta ^\prime })$ system; one already knows from the preceding section that it provides a consistent picture of the $\tau$ sector as it renders consistent the account of the A, C and B spectra.

Once these symmetries have been broken, the PS kinetic energy term of the HLS Lagrangian is no longer diagonal, and a field redefinition is mandatory to restore its canonical form.^18^ This is performed in the two steps addressed right now.

### Diagonalization of the $\mathcal{L}_{A}$ PS kinetic energy piece

In the BHLS/$\hbox {BHLS}_2$ model, the pseudoscalar (PS) kinetic energy term is written as [14, 19]:

9 $$\begin{aligned} \displaystyle \mathcal{L}_{A,\mathrm{kin}} = \mathrm{Tr} \left[ \partial P_\mathrm{bare} X_A \partial P_\mathrm{bare} X_A\right] , \end{aligned}$$

where $X_A$ is the so-called BKY breaking matrix at work in the $\mathcal{L}_{A}$ sector of the $\hbox {BHLS}_2$ non-anomalous Lagrangian [19] ($\mathcal{L}= \mathcal{L}_A+ a \mathcal{L}_V$); combining the new breaking scheme defined in [17] and the extension proposed in [18], we write [14]:

10 $$\begin{aligned} \left\{ \begin{array}{ll} \displaystyle X_A=\mathrm{Diag} [q_A, y_A,z_A] \\ \displaystyle q_A=1+\frac{\Sigma _A+\Delta _A}{2},&{} \displaystyle y_A=1+\frac{\Sigma _A-\Delta _A}{2}. \end{array} \right. \end{aligned}$$

The departure from unity of the $(u,{\overline{u}})$ and $(d,{\overline{d}})$ entries ($q_A$ and $y_A$) of $X_A$, numerically small [14], are treated as $\mathcal{O} (\delta )$ perturbations^19^ in amplitude calculations whereas $z_A$ occurring as the $X_A$ $(s,{\overline{s}})$ entry is expected and treated as $\mathcal{O} (1)$; this entry can be also referred to as flavor breaking [35–37]. Assuming the pion decay constant $f_\pi$ occurring in the HLS-based Lagrangian models is the observed one, its renormalization is unnecessary and has been shown to imply [14] $\Sigma _A=0$. Therefore, phenomenologically, one is left with only two free parameters, $\Delta _A$ and $z_A$.

To restore the PS kinetic energy of the $\mathcal{L}_{A}$ piece of the BKY broken HLS Lagrangians to canonical form, a first field transform [31] is performed:

11 $$\begin{aligned} P_{R1} = X_A^{1/2} P_\mathrm{bare} X_A^{1/2}; \end{aligned}$$

$P_{R1}$ is the (first step) renormalized PS field matrix which brings $\mathcal{L}_{A,\mathrm{kin}}$ back into canonical form. One has:

12 $$\begin{aligned} \left( \begin{array}{l} \displaystyle \pi ^3_\mathrm{bare} \\ \displaystyle \eta ^0_\mathrm{bare} \\ \displaystyle \eta ^8_\mathrm{bare} \end{array} \right)= & {} \left( \begin{array}{ccc} 1 &{} \displaystyle -\frac{\Delta _A}{\sqrt{6}} &{} \displaystyle -\frac{\Delta _A}{2\sqrt{3}} \\ \displaystyle - \frac{\Delta _A}{\sqrt{6}} &{} \displaystyle B &{} \displaystyle A \\ \displaystyle -\frac{\Delta _A}{2\sqrt{3}} &{} \displaystyle A&{} \displaystyle C \end{array} \right) \left( \begin{array}{l} \displaystyle \pi ^3_{R1} \\ \displaystyle \eta ^0_{R1} \\ \displaystyle \eta ^8_{R1} \end{array} \right) \nonumber \\= & {} W \left( \begin{array}{l} \displaystyle \pi ^3_{R1} \\ \displaystyle \eta ^0_{R1} \\ \displaystyle \eta ^8_{R1} \end{array} \right) \end{aligned}$$

which defines the matrix *W*. We use the notation $\pi ^3$ to recall the specific Gell–Mann matrix to which the neutral pion is associated, and devote the notation $\pi ^0$ to the corresponding mass eigenstate. The $(\eta _0,\eta _8)$ entries of *W* in Eq. (12) are given by:

13 $$\begin{aligned} \displaystyle A= & {} \sqrt{2} \frac{z_A-1}{3z_A} ,\quad B= \displaystyle \frac{2 z_A+1}{3z_A} ,\nonumber \\ C= & {} \displaystyle \frac{z_A+2}{3z_A} , \displaystyle \left( BC-A^2 = \frac{1}{z_A}\right) \end{aligned}$$

which are used all along this study. For further use, Eq. (12) is re-expressed:

14 $$\begin{aligned} \displaystyle \mathcal{V}_\mathrm{bare}= & {} W \mathcal{V}_{R1}, ~~ \mathrm{with}~~\displaystyle \mathcal{V}_\mathrm{any}^t\nonumber \\= & {} ( \pi ^3_\mathrm{any},~\eta ^0_\mathrm{any},~~\eta ^8_\mathrm{any}) \quad (\mathrm{any} =\mathrm{bare},R_1). \end{aligned}$$

In terms of the *R*1 renormalized fields, $\mathcal{L}_{A,\mathrm{kin}}$ is thus canonical:

15 $$\begin{aligned} \mathcal{L}_{A,\mathrm{kin}} =\displaystyle \frac{1}{2} \left\{ \left[ \partial _\mu \pi ^3_{R1}\right] ^2 +\left[ \partial _\mu \eta ^0_{R1}\right] ^2 + \left[ \partial _\mu \eta ^8_{R1}\right] ^2 \right\} . \end{aligned}$$

The following expression of $X_A$ in the *U*(3) algebra canonical basis clearly exhibits the precise structure of the BKY breaking procedure:

16 $$\begin{aligned} X_A= I + \left\{ \Delta _A T_3 + \sqrt{\frac{2}{3}} \left[ z_A-1 \right] \left[ T_0 -\sqrt{2}~ T_8 \right] \right\} , \end{aligned}$$

where *I* is the unit matrix. $T_0=I/\sqrt{6}$ complements the usual Gell–Mann matrices normalized by Tr$[T_a T_b] =\delta _{a b} /2$.

Displayed in this way, the departure from unity of the $X_A$ breaking matrix exhibits its three expected contributions. $\Delta _A$, a purely isospin symmetry breaking (ISB) parameter, is associated with $T_3 =\mathrm{Diag} (1,-1,0)/2$, as it should be. In contrast, the effect of the flavor breaking amount $z_A - 1$ will be met several times below. Its origin is naturally shared between $T_0 =\mathrm{Diag} (1,1,1)/\sqrt{6}$ and $T_8 =\mathrm{Diag} (1,1,-2)/2\sqrt{3}$; both simultaneously vanish in the “no-BKY breaking” limit $z_A=1$. So, as expected, the BKY matrix $(s,{\overline{s}})$ entry, combines correlatedly SU(3) and nonet symmetry (NSB) breakings in the PS sector. Let us also note that [19] $z_A =[f_K/f_\pi ]^2 + \mathcal{O}(\delta )$, as will be noted below in the $\hbox {EBHLS}_2$ modified context – see Eq. (71).

### The kinetic breaking: a generalization of the ’t Hooft term

A more direct breaking of the *U*(3) symmetric PS field matrix to SU$(3)\times U(1)$ has also been found phenomenologically requested to successfully deal with the whole BHLS realm of experimental data [14, 19]. These are the so-called ’t Hooft determinant terms [20, 31–33]; limiting ourselves here to the kinetic energy term, we have been led to supplement the HLS kinetic energy piece by:

17 $$\begin{aligned} \displaystyle \mathcal{L}_\mathrm{'tHooft}= & {} \lambda \frac{f_\pi ^2}{12} \mathrm{Tr} \ln {\partial _\mu U} \times \mathrm{Tr} \ln {\partial ^\mu U^\dagger },\nonumber \\ U= & {} \xi _L^\dagger \xi _R= \exp {[2i P /f_\pi ]}, \end{aligned}$$

where *P* is the usual *U*(3) symmetric pseudoscalar bare field matrix [14, 19], and $f_\pi$ the (measured) charged pion decay constant. This relation is connected with $\det {\partial U}$ by the identity:

$$\begin{aligned} \ln {\mathrm{det}\partial _\mu U}= \ \mathrm{Tr} \ln {\partial _\mu U}. \end{aligned}$$

Expanding $\ln {\partial _\mu U}$ in Eq. (17), the leading order term is^20^:

18 $$\begin{aligned} \displaystyle \mathcal{L}_\mathrm{'tHooft}= \frac{\lambda }{2} \partial _\mu \eta ^0_\mathrm{bare} \partial ^\mu \eta ^0_\mathrm{bare} \end{aligned}$$

only involving the singlet PS bare field $\eta ^0_\mathrm{bare}$.

The ’t Hooft term tool, already used in the previous broken HLS versions, can be fruitfully generalized. Indeed, Eq. (17) can be interpreted as:

19 $$\begin{aligned} \displaystyle \mathcal{L}_\mathrm{'tHooft}= \frac{f_\pi ^2}{2} \mathrm{Tr} \ln { X_H \partial _\mu U} \times \mathrm{Tr} \ln {X_H \partial ^\mu U^\dagger } \end{aligned}$$

where^21^$$X_H=\sqrt{\lambda } T_0$$.

Equation (19) gives a hint that other well-chosen forms of the $X_H$ matrix may exhibit interesting properties. Indeed, it clearly permits us to define mechanisms not limited to only nonet symmetry breaking. This leads us to propose the following choice for $X_H$:

20 $$\begin{aligned} \displaystyle X_H = \lambda _0 T_0 +\lambda _3 T_3+\lambda _8 T_8 \end{aligned}$$

as it manifestly allows for a breaking of isospin symmetry and enriches the ability of the HLS model to cover the $(\pi ^0, ~\eta , ~\eta ^\prime )$ mixing properties. As will be seen shortly, it also leads to differentiate the pion pair couplings to $\gamma$ and $W^\pm$. With this choice, Eq. (19) becomes at leading order:

21 $$\begin{aligned} \displaystyle \mathcal{L}_\mathrm{'tHooft}= & {} \frac{1}{2} \left[ \lambda _3 \partial _\mu \pi ^3 +\lambda _0 \partial _\mu \eta ^0+\lambda _8 \partial _\mu \eta ^8 \right] \nonumber \\&\times \left[ \lambda _3 \partial ^\mu \pi ^3 +\lambda _0 \partial ^\mu \eta ^0+\lambda _8 \partial ^\mu \eta ^8 \right] . \end{aligned}$$

This form for $X_H$ is certainly not the only way to generalize the usual ’t Hooft term. For instance, among other possible choices, one could quote:

22 $$\begin{aligned} \displaystyle \mathcal{L}_\mathrm{'tHooft}= & {} \frac{f_\pi ^2}{2} \sum _{a=0,3,8} \mathrm{Tr} \ln { X_{H_a} \partial _\mu U} \times \mathrm{Tr} \ln {X_{H_a} \partial ^\mu U^\dagger }, \nonumber \\ \displaystyle X_{H_a}= & {} \lambda _a T_a, \end{aligned}$$

which turns out to drop the crossed terms in Eq. (21) – and in all expressions reported below. On the other hand, as will be seen in Sect. 16, it happens that a generalization such as Eq. (20) is necessary to allow $\hbox {BHLS}_2$ to fulfill expected properties of the axial currents in a nontrivial way.

In order to deal with kaons or charged pions, one could also define appropriate projectors $X_H$; however, this does not appear necessary, as the BKY breaking already produces the needed breaking effects [14, 19].

In the broken HLS frameworks previously defined, the (single) ’t Hooft breaking parameter $\lambda ~(=\lambda _0^2)$ was counted as $\mathcal{O}(\delta )$ when truncating the Lagrangian to its leading order terms in all the previously defined $\hbox {BHLS}_2$ breaking parameters [19]. Consistency, thus, implies counting all the $\lambda _i$ just introduced as $\mathcal{O}(\delta ^{1/2})$.

### The PS kinetic energy of the extended $\hbox {BHLS}_2$ Lagrangian

The full PS kinetic energy term of the broken HLS Lagrangians is provided by their $\mathcal{L}_{A}^\prime \equiv \mathcal{L}_{A} +\mathcal{L}_\mathrm{'t~Hooft}$ Lagrangian piece:

23 $$\begin{aligned} \displaystyle \mathcal{L}_\mathrm{kin}^\prime = \mathrm{Tr} \left[ \partial P_\mathrm{bare} X_A \partial P_\mathrm{bare} X_A\right] + 2\, \{ \mathrm{Tr} \left[ X_H \partial P_\mathrm{bare} \right] \}^2 . \end{aligned}$$

Performing the change of fields of Eq. (12) which diagonalizes $\mathcal{L}_{A,\mathrm{kin}}$ and using $X_H$ as given in Eq. (20), the full kinetic energy term $\mathcal{L}_\mathrm{kin}^\prime$ can be written:

24 $$\begin{aligned} \displaystyle \mathcal{L}_\mathrm{kin}^\prime= & {} \displaystyle \frac{1}{2} \left[ (1+\lambda _3^2) \partial _\mu \pi ^3_{R1} \partial ^\mu \pi ^3_{R1} +(1+{\widetilde{\lambda }}_0^2) \partial _\mu \eta ^0_{R1} \partial ^\mu \eta ^0_{R1}\right. \nonumber \\&+(1+{\widetilde{\lambda }}_8^2) \partial _\mu \eta ^8_{R1} \partial ^\mu \eta ^8_{R1}+ 2 {\widetilde{\lambda }}_0 {\widetilde{\lambda }}_8~ \eta ^0_{R1} \eta ^8_{R1} \nonumber \\&\displaystyle \left. +2\lambda _3{\widetilde{\lambda }}_0~\pi ^3_{R1}\eta ^0_{R1} + 2\lambda _3{\widetilde{\lambda }}_8~\pi ^3_{R1}\eta ^8_{R1} \right] , \end{aligned}$$

omitting the kaon and (charged) pion terms which are standard and displayed elsewhere [14, 19]. We have defined:

25 $$\begin{aligned} \begin{array}{ll} \displaystyle {\widetilde{\lambda }}_0= \lambda _0 B + \lambda _8 A,&\displaystyle {\widetilde{\lambda }}_8= \lambda _0 A + \lambda _8 C \end{array} \end{aligned}$$

where *A*, *B*, *C* are given by Eq. (13). One should note the intricate combination of the ’t Hooft breaking parameters with the BKY parameter $z_A$.

Defining the (co-)vector $\mathcal{V}^t_{R1} = (\pi ^3_{R1},~\eta ^0_{R1},~\eta ^8_{R1})$, $\mathcal{L}_\mathrm{kin}^\prime$ can be written:

26 $$\begin{aligned} \displaystyle \mathcal{L}_\mathrm{kin}^\prime = \frac{1}{2} ~\mathcal{V}_{R1}^t \! \cdot \! M \! \cdot \! \mathcal{V}_{R1}, \end{aligned}$$

*M* being the sum of the unit matrix and of a rank 1 we write:

27 $$\begin{aligned} \begin{array}{ll} \displaystyle M = 1+ a \cdot a^t ,&\mathrm{where} ~ a^t=(\lambda _3,~{\widetilde{\lambda }}_0,~{\widetilde{\lambda }}_8). \end{array} \end{aligned}$$

The second step renormalized fields $\mathcal{V}^t_{R} = (\pi ^3_{R},~\eta ^0_{R},~\eta ^8_{R})$ are defined by:

28 $$\begin{aligned} \displaystyle \mathcal{V}_{R} = \left[ 1+ \frac{1}{2}a \cdot a^t \right] \cdot \mathcal{V}_{R1} \end{aligned}$$

which brings the kinetic energy into canonical form:

29 $$\begin{aligned} \displaystyle \mathcal{L}_\mathrm{kin}^\prime = \frac{1}{2} ~\mathcal{V}_{R}^t \cdot \mathcal{V}_{R} + \mathcal{O}(\delta ^2). \end{aligned}$$

At the same order, one has:

30 $$\begin{aligned} \displaystyle \mathcal{V}_{R1} = \left[ 1- \frac{1}{2}a \cdot a^t \right] \cdot \mathcal{V}_{R} + \mathcal{O}(\delta ^2) \end{aligned}$$

and, finally, using Eqs. (13) and (14):

31 $$\begin{aligned} \displaystyle \mathcal{V}_\mathrm{bare} = W \cdot \left[ 1- \frac{1}{2}a \cdot a^t \right] \cdot \mathcal{V}_{R}+ \mathcal{O}(\delta ^2), \end{aligned}$$

where *W* is defined in Eq. (12).

## PS meson mass eigenstates: the physical PS field basis

The PS field *R* basis renders the kinetic energy term canonical; nevertheless, this *R* basis is not expected to diagonalize the PS mass term into its mass eigenstates ($\pi ^0,~\eta ,~{\eta ^\prime }$). Indeed, for instance, up to small perturbations, the $\eta ^0_R$ and $\eta ^8_R$ are almost pure singlet and octet field combinations, while the physically observed $\eta$ and ${\eta ^\prime }$ mass eigenstate fields are mixtures of these. In order to preserve the canonical structure of the PS kinetic energy one should consider the transformation from *R* fields to the physically observed mass eigenstates; as the PS mass term (not shown) is certainly a positive definite quadratic form, this transformation should be a pure rotation.

In the traditional approach, the physical $\eta$ and ${\eta ^\prime }$ fields are related to the singlet-octet states by the so-called one-angle transform:

32 $$\begin{aligned} \left( \begin{array}{l} \displaystyle \eta \\ \displaystyle \eta ^\prime \end{array} \right) = \left( \begin{array}{cc} \displaystyle \cos {\theta _P}&{}\displaystyle -\sin {\theta _P} \\ \displaystyle \sin {\theta _P}&{}\displaystyle \cos {\theta _P} \end{array} \right) \left( \begin{array}{l} \displaystyle \eta ^8_R \\ \displaystyle \eta ^0_R \end{array} \right) . \end{aligned}$$

However, extending to the mass eigenstate ($\pi ^0,~\eta ,~{\eta ^\prime }$) triplet, one expects a three-dimensional rotation and thus three angles. Adopting the Leutwyler parameterization [39], one has:

33 $$\begin{aligned}&\left( \begin{array}{l} \displaystyle \pi ^3_R \\ \displaystyle \eta ^8_R \\ \displaystyle \eta ^0_R \end{array} \right) = \left( \begin{array}{ccc} \displaystyle 1 &{} \displaystyle -\epsilon &{} \displaystyle -\epsilon ^\prime \\ \displaystyle \epsilon \cos {\theta _P}+\epsilon ^\prime \sin {\theta _P} &{} \displaystyle \cos {\theta _P} &{}\displaystyle \sin {\theta _P} \\ \displaystyle -\epsilon \sin {\theta _P}+\epsilon ^\prime \cos {\theta _P} &{} \displaystyle -\sin {\theta _P} &{}\displaystyle \cos {\theta _P} \end{array} \right) \nonumber \\&\quad \quad \quad \times \left( \begin{array}{l} \displaystyle \pi ^0 \\ \displaystyle \eta \\ \displaystyle {\eta ^\prime }\end{array} \right) \end{aligned}$$

to relate the *R* fields which diagonalize the kinetic energy to the physical (i.e. mass eigenstates) neutral PS fields. The three angles occurring there ($\epsilon$, $\epsilon ^\prime$ and even $\theta _P$) are assumed $\mathcal{O}(\delta )$ perturbations; nevertheless, it looks better to stick close to the one-angle picture by keeping the trigonometric functions of $\theta _P$, the so-called third mixing angle [31]; for clarity and for the sake of comparison with other works, $\theta _P$ is not treated as manifestly small. The Leutwyler rotation matrix can be factored out into a product of two rotation matrices:

34 $$\begin{aligned}&\left( \begin{array}{ccc} \displaystyle 1 &{} \displaystyle -\epsilon &{} \displaystyle -\epsilon ^\prime \\ \displaystyle \epsilon \cos {\theta _P}+\epsilon ^\prime \sin {\theta _P} &{} \displaystyle \cos {\theta _P} &{}\displaystyle \sin {\theta _P} \\ \displaystyle -\epsilon \sin {\theta _P}+\epsilon ^\prime \cos {\theta _P} &{} \displaystyle -\sin {\theta _P} &{}\displaystyle \cos {\theta _P} \end{array} \right) \nonumber \\&\quad = \left( \begin{array}{ccc} \displaystyle 1 &{} \displaystyle 0 &{} \displaystyle 0 \\ \displaystyle 0 &{} \displaystyle \cos {\theta _P} &{}\displaystyle \sin {\theta _P} \\ \displaystyle 0 &{} \displaystyle -\sin {\theta _P} &{}\displaystyle \cos {\theta _P} \end{array} \right) \left( \begin{array}{lll} \displaystyle 1 &{} \displaystyle -\epsilon &{} \displaystyle -\epsilon ^\prime \\ \displaystyle \epsilon &{} \displaystyle 1 &{}\displaystyle 0 \\ \displaystyle \epsilon ^\prime &{} \displaystyle 0 &{}\displaystyle 1 \end{array} \right) . \end{aligned}$$

Substantially, the second matrix in the right-hand side of Eq. (34) reflects isospin breaking effects. In the following, we name $M(\theta _P)$ and $M(\epsilon )$ the two matrices showing up there; they fulfill:

35 $$\begin{aligned} \displaystyle \left[ M(\theta _P) \cdot M(\epsilon ) \right] ^{-1}= {\widetilde{M}}(\epsilon ){\widetilde{M}}(\theta _P) \end{aligned}$$

up to terms of degree higher than 1 in $\delta$. This implies [39]:

36 $$\begin{aligned} \left( \begin{array}{l} \displaystyle \pi ^0\\ \displaystyle \eta \\ \displaystyle {\eta ^\prime }\end{array} \right)= & {} \left( \begin{array}{ccc} \displaystyle 1 &{}\displaystyle \epsilon \cos {\theta _P}+\epsilon ^\prime \sin {\theta _P} &{} \displaystyle -\epsilon \sin {\theta _P}+\epsilon ^\prime \cos {\theta _P} \\ \displaystyle -\epsilon &{} \displaystyle \cos {\theta _P} &{}\displaystyle -\sin {\theta _P} \\ \displaystyle -\epsilon ^\prime &{}\sin {\theta _P} &{} \cos {\theta _P} \end{array} \right) \nonumber \\&\times \left( \begin{array}{l} \displaystyle \pi ^3_R \\ \displaystyle \eta ^8_R \\ \displaystyle \eta ^0_R \end{array} \right) . \end{aligned}$$

As for their perturbative order, $\epsilon$ and $\epsilon ^\prime$ are treated as $\mathcal{O}(\delta )$. Equations (36) and (31) allow us to define the (linear) relationship between the physical $\pi ^0$, $\eta$ and ${\eta ^\prime }$ states and their bare partners occurring in the original HLS Lagrangians.

## Extended $\hbox {BHLS}_2$: the non-anomalous Lagrangian

The non-anomalous $\hbox {EBHLS}_2$ Lagrangian in the present approach can also be written:

37 $$\begin{aligned} \displaystyle \mathcal{L}_\mathrm{HLS} = \mathcal{L}_{A}+a \mathcal{L}_{V} +\mathcal{L}_{p^4}, \end{aligned}$$

as in [19]. As in this Reference, one splits up the first two terms in a more appropriate way:

38 $$\begin{aligned} \displaystyle \mathcal{L}_{A}+a \mathcal{L}_{V} = \mathcal{L}_\mathrm{VMD}+\mathcal{L}_{\tau }. \end{aligned}$$

$\mathcal{L}_\mathrm{VMD}$ essentially addresses the physics of the $e^+e^-$ annihilations to charged pions and to kaons pairs; within the present breaking scheme, it remains strictly identical to those displayed in Appendix A.1 of [19]. The $\mathcal{L}_{p^4}$ is also unchanged, as it does not address PS meson interactions; it is identical to those displayed in Appendix A.3 of [19]. Both pieces have not to be discussed any further, and their expressions will not be reproduced here to avoid lengthy repetition.

All modifications induced by the generalized ’t Hooft kinetic breaking mechanism are concentrated in the $\mathcal{L}_{\tau }$ piece and are displayed right now. Using^22^ ($m^2=a g^2 f_\pi ^2$):

39 $$\begin{aligned} \displaystyle m_{\rho ^\pm }^2=m^2\left[ 1+ \Sigma _V \right] ,\quad f_{\rho W} = a g f_\pi ^2 \left[ 1+ \Sigma _V \right] , \end{aligned}$$

the expression of $\mathcal{L}_{\tau }$ in terms of bare PS fields is given, at lowest order in the breaking parameters, by:

40 $$\begin{aligned} \mathcal{L}_{\tau }= & {} \displaystyle - \frac{i V_{ud}g_2}{2} W^+ \cdot \left[ (1 -\frac{a (1+ \Sigma _V )}{2} ) \pi ^- {\mathop {\partial }\limits ^{\leftrightarrow }}\pi ^3_b\right. \nonumber \\&\quad \left. +\frac{1}{\sqrt{2}} [1 -\frac{a}{2z_A}(1+ \Sigma _V )] K^0 {\mathop {\partial }\limits ^{\leftrightarrow }}K^- \right] \nonumber \\&\quad \displaystyle + m_{\rho ^\pm }^2 \rho ^+ \cdot \rho ^- -\frac{g_2V_{ud}}{2} f_{\rho W} W^+ \cdot \rho ^- \nonumber \\&\quad + \frac{i a g }{2} (1+ \Sigma _V) \rho ^- \cdot \left[ \pi ^+ {\mathop {\partial }\limits ^{\leftrightarrow }}\pi ^3_b +\frac{1}{z_A\sqrt{2}}{\overline{K}}^0 {\mathop {\partial }\limits ^{\leftrightarrow }}K^+ \right] \nonumber \\&\quad \displaystyle + \frac{f_\pi ^2 g_2^2}{4} \left\{ \left[ \displaystyle [(1+\frac{\Delta _A}{2}) z_A +a z_V (1+ \frac{\Sigma _V }{2})] |V_{us}|^2 \right. \right. \nonumber \\&\quad \left. \left. + [1+a(1+ \Sigma _V )] |V_{ud}|^2\right] \right\} W^+ \cdot W^- \nonumber \\&\quad \displaystyle +\frac{1}{9} a e^2 f_\pi ^2 \left( 5+5\Sigma _V+z_V\right) A^2, \end{aligned}$$

where we have limited ourselves to displaying only the terms relevant for our purpose. The (classical) photon and *W* mass terms [13, 17] are not considered and are given only for completeness. However, it is worth recalling that the photon mass term does not prevent the photon pole from residing at $s=0$ as required [62], at leading order. The interaction part of $\mathcal{L}_{\tau }$ can be split into several pieces. Discarding couplings of the form $W K \eta _8$, we can write:

41 $$\begin{aligned} \mathcal{L}_{\tau } = \displaystyle \mathcal{L}_{\tau ,K} +\mathcal{L}_{\tau ,K}^\dagger +\mathcal{L}_{\tau ,\pi } +\mathcal{L}_{\tau ,\pi }^\dagger + m_{\rho ^\pm }^2 \rho ^+ \cdot \rho ^- \end{aligned}$$

with:

42 $$\begin{aligned} \displaystyle \mathcal{L}_{\tau ,K}= & {} -\frac{i}{2\sqrt{2}} \left\{ g_2 V_{ud} \left[ 1 -\frac{a}{2z_A}(1+ \Sigma _V ) \right] W^+ \right. \nonumber \\&\left. +\frac{a g }{z_A} (1+ \Sigma _V)\rho ^+ \right\} \cdot ~ K^0 {\mathop {\partial }\limits ^{\leftrightarrow }}K^- \end{aligned}$$

and:

43 $$\begin{aligned} \displaystyle \mathcal{L}_{\tau ,\pi }= & {} \displaystyle - \frac{i}{2} \left\{ g_2 V_{ud} \left[ 1 -\frac{a}{2}(1+ \Sigma _V ) \right] W^{+} + a g (1+ \Sigma _V)\rho ^+ \right\} \nonumber \\&\cdot \pi ^- {\mathop {\partial }\limits ^{\leftrightarrow }}\pi ^3_b -\frac{g_2V_{ud}}{2} f_{\rho W} W^+ \! \cdot \!\rho ^- \end{aligned}$$

where the subscript *b* indicates that the $\pi ^0$ field is bare. Equation (31) provides the relationship between bare and renormalized states, in particular:

44 $$\begin{aligned} \pi ^3_b= & {} \displaystyle \left\{ 1 -\frac{\lambda _3^2}{2} \right\} \pi ^3_R - \left\{ \frac{1}{\sqrt{6}} \Delta _A + \frac{\lambda _3 {\widetilde{\lambda }}_0}{2} \right\} \eta ^0_R \nonumber \\&- \left\{ \frac{1}{2\sqrt{3}} \Delta _A + \frac{\lambda _3 {\widetilde{\lambda }}_8}{2} \right\} \eta ^8_R . \end{aligned}$$

The occurrence of the $\lambda _3$ parameter generates a decoupling of the $W \pi ^\pm \pi ^0$ and $A \pi ^+\pi ^-$ interaction intensities. Also using Eq. (36), Eqs. (43) and (44) give at first non-leading order:

45 $$\begin{aligned} \left\{ \begin{aligned} \displaystyle \mathcal{L}_{\pi ^0 \pi ^\pm }&= \displaystyle - \frac{i}{2} \left\{ g_2 V_{ud} \left[ 1 -\frac{a}{2}(1+ \Sigma _V ) \right] W^+ \right. \\&\quad \left. + a g (1+ \Sigma _V)\rho ^+ \right\} \left\{ 1 -\frac{\lambda _3^2}{2} \right\} \cdot \! \pi ^- {\mathop {\partial }\limits ^{\leftrightarrow }}\pi ^0 ,\\ \displaystyle \mathcal{L}_{\eta \pi ^\pm } =&\displaystyle + \frac{i}{2} \left\{ g_2 V_{ud} \left[ 1 -\frac{a}{2}(1+ \Sigma _V ) \right] W^+ \right. \\&\quad \left. + a g (1+ \Sigma _V)\rho ^+ \right\} \\&\quad \displaystyle \times \left[ \left\{ \frac{1}{\sqrt{6}} \Delta _A + \frac{\lambda _3 {\widetilde{\lambda }}_0}{2} \right\} \cos {\theta _P} \right. \\&\quad \left. -\left\{ \frac{1}{2\sqrt{3}} \Delta _A + \frac{\lambda _3 {\widetilde{\lambda }}_8}{2} \right\} \sin {\theta _P} +\epsilon \right] \cdot \! \pi ^- {\mathop {\partial }\limits ^{\leftrightarrow }}\eta ,\\ \displaystyle \mathcal{L}_{{\eta ^\prime }\pi ^\pm }&= \displaystyle + \frac{i}{2} \left\{ g_2 V_{ud} \left[ 1 -\frac{a}{2}(1+ \Sigma _V ) \right] W^+ \right. \\&\quad \left. + a g (1+ \Sigma _V)\rho ^+ \right\} \\&\quad \displaystyle \times \left[ \left\{ \frac{1}{2\sqrt{3}} \Delta _A + \frac{\lambda _3 {\widetilde{\lambda }}_8}{2} \right\} \cos {\theta _P} +\left\{ \frac{1}{\sqrt{6}} \Delta _A \right. \right. \\&\quad \left. \left. + \frac{\lambda _3 {\widetilde{\lambda }}_0}{2} \right\} \sin {\theta _P} +\epsilon ^\prime \right] \cdot \! \pi ^- {\mathop {\partial }\limits ^{\leftrightarrow }}{\eta ^\prime }, \end{aligned}\right. \end{aligned}$$

in terms of physical neutral PS fields, and then:

46 $$\begin{aligned} \mathcal{L}_{\tau ,\pi }= & {} \mathcal{L}_{\pi ^0 \pi ^\pm }+\mathcal{L}_{\eta \pi ^\pm }+\mathcal{L}_{{\eta ^\prime }\pi ^\pm } -\frac{g_2V_{ud}}{2} f_{\rho W} W^+ \! \cdot \!\rho ^- \nonumber \\&+{\mathrm h.c.} + m_{\rho ^\pm }^2 \rho ^+ \cdot \rho ^- . \end{aligned}$$

Once more, the rest of the $\mathcal{L}_A+ a \mathcal{L}_V$ is unchanged compared to their $\hbox {BHLS}_2$ expressions [19].

Regarding the pion form factor in the $\tau$ decay, the changes versus Sect. 11.1 in [19] and the present $\hbox {EBHLS}_2$ are very limited:

47 $$\begin{aligned} \left\{ \begin{array}{lll} W^\mp \pi ^\pm \pi ^0 ~~\mathrm{coupling:}&{} \displaystyle \left[ 1 -\frac{a}{2}(1+ \Sigma _V ) \right] \\ &{}\Longrightarrow \displaystyle \left[ 1 -\frac{a}{2}(1+ \Sigma _V ) \right] \left\{ 1 -\frac{\lambda _3^2}{2} \right\} ,\\ \rho ^\mp \pi ^\pm \pi ^0 ~~\mathrm{coupling:}&{} \displaystyle ag (1+ \Sigma _V )\\ &{} \Longrightarrow \displaystyle ~ag (1+ \Sigma _V ) \left\{ 1 -\frac{\lambda _3^2}{2} \right\} . \end{array} \right. \end{aligned}$$

This implies a global rescaling of the $\hbox {BHLS}_2$ pion form factor $F_\pi ^\tau (s)$ by $1-\lambda _3^2/2$; it also implies that the $\pi ^0 \pi ^\pm$ loop acquires a factor of $[1-\lambda _3^2]$. The $\hbox {BHLS}_2$ $W^\pm -\rho ^\mp$ transition amplitude $F_\rho ^\tau (s)$ and the $\rho ^\pm$ propagator $[D_\rho (s)]^{-1}$ are changed correspondingly (see Sect. 11.1 in [19]). On the other hand, $F_\pi ^e(s)$ remains identical to its $\hbox {BHLS}_2$ expression, as well as both kaon form factors.

## Extended $\hbox {BHLS}_2$: the anomalous Lagrangian pieces

If only the $\mathcal{L}_\tau$ part of the non-anomalous $\hbox {BHLS}_2$ Lagrangian is affected by the kinetic breaking presented above, all the anomalous FKTUY pieces [12, 13] are concerned.

The Lagrangian pieces of relevance for the phenomenology we address are, on the one hand:

48 $$\begin{aligned} \left\{ \begin{array}{lll} \displaystyle \mathcal{L}_{AAP}= -\frac{3 \alpha _{em}}{\pi f_\pi }(1-c_4) ~\epsilon ^{\mu \nu \alpha \beta } \partial _\mu A_\nu \partial _\alpha A_\beta \mathrm {Tr} \left[ Q^2 P \right] ,\\ \displaystyle \mathcal{L}_{VVP}= -\frac{3 g^2}{4 \pi ^2 f_\pi }~c_3 ~\epsilon ^{\mu \nu \alpha \beta } \mathrm {Tr} \left[ \partial _\mu V_\nu \partial _\alpha V_\beta P \right] , \end{array} \right. \end{aligned}$$

where *Q* is the usual quark charge matrix and *A*, *V* and *P* respectively denote the electromagnetic field, the vector field matrix and the U(3) symmetric bare pseudoscalar field matrix as defined in [14] regarding their normalization. As we did not find any important improvement by assuming $c_3 \ne c_4$, the difference of these has been set to zero; consequently, the $\mathcal{L}_{AVP}$ Lagrangian piece [13] drops out.

On the other hand, the following pieces should also be considered:

49 $$\begin{aligned} \left\{ \begin{array}{lll} &{}\displaystyle \mathcal{L}_{APPP}=-i\frac{3 e}{3 \pi ^2 f_\pi ^3} &{}\left[ 1-\frac{3}{4}(c_1-c_2+c_4)\right] \\ &{} &{}~\epsilon ^{\mu \nu \alpha \beta } A_\mu \mathrm {Tr} \left[ Q ~\partial _\nu P \partial _\alpha P \partial _\beta P\right] , \\ &{} \displaystyle \mathcal{L}_{VPPP}= -i\frac{3 g}{4 \pi ^2 f_\pi ^3} &{} \left[ c_1-c_2-c_3\right] \displaystyle ~\epsilon ^{\mu \nu \alpha \beta } \mathrm {Tr} \\ &{} &{} \left[ V_\mu \partial _\nu P \partial _\alpha P \partial _\beta P\right] . \end{array} \right. \end{aligned}$$

These involve, besides $c_3$ and $c_4$, a third parameter $c_1-c_2$ which is also not fixed within the HLS framework [13] and should be derived from the minimization procedure. For easier reading of the text, we have found it worth pushing them into Appendices A and B.

Regarding the pseudoscalar fields, the Lagrangian pieces listed in Appendices A and B are expressed in terms of the physically observed $\pi ^0,~\eta ,~{\eta ^\prime }$ whereas, for simplicity, the vector mesons are expressed in terms of their ideal combinations: $\rho ^0_I,~\omega _I$ and $\phi _I$. The procedures to derive the couplings to the physically observed $\rho ^0,~\omega$ and $\phi$ and to construct the cross sections for the $e^+e^- \rightarrow (\pi ^0/\eta ) \gamma$ annihilations are given in full detail in Sect. 12 of [19]. Nevertheless, we have found it worthwhile to construct the amplitude and the cross section for the $e^+e^- \rightarrow \pi ^0 \pi ^+ \pi ^-$ annihilations in the extended $\hbox {BHLS}_2$ framework; this information^23^ is provided in Appendix C.

## Update of the 3$\pi$ annihilation channel

The BESIII Collaboration has recently published the Born cross section spectrum [24] for the $e^+e^- \rightarrow \pi ^+ \pi ^- \pi ^0$ annihilation over the $0.7 \div 3.0$ GeV energy range collected in the ISR mode. This new data sample complements the spectra collected at the VEPP-2M Collider by CMD2 [43, 63–65] and SND [66, 67] covering the $\omega$ and $\phi$ peak regions. Besides, the only data on the 3$\pi$ cross section stretching over the intermediate region were collected much earlier [68] by the former neutral detector (ND). As the measurement by BaBar [69] only covers the $\sqrt{s} > 1.05$ GeV region, it is of no concern for physics studies up to the $\phi$ signal. On the other hand, previous independent analyses [19, 70] indicate that the CMD2 spectrum [65] returns an average $\chi ^2$ per point, much above 2 units, which led to discard it from global approaches.

The BESIII sample [24] is the first three-pion sample to encompass the whole range of validity of the HLS model, providing a doubling of the number of candidate data points and, additionally, the first cross-check of the cross section behavior in the energy region between the narrow $\omega$ and $\phi$ signals.

We first examine how it fits within the global HLS framework in isolation (i.e. as a single representative of the 3$\pi$ annihilation channel) and determine its consistency with the already analyzed data samples covering the *other* annihilation channels, namely $\pi \pi$, $(\pi ^0/\eta ) \gamma$ and both $K {\overline{K}}$ modes. A second step is devoted to consistency studies between the BESIII spectrum and those previously collected in the same 3$\pi$ channel by the ND [68], CMD2 [43, 63, 64] and SND [66, 67] detectors.

### The BESIII 3$\pi$ data sample in isolation within $\hbox {EBHLS}_2$

The fit procedure already developed within the previous BHLS frameworks [14, 19] – and closely followed here – relies on a global $\chi ^2$ minimization. In order to include the BESIII sample within the $\hbox {EBHLS}_2$ framework,^24^ one should first define its contribution to the global $\chi ^2$. This requires us to define the error covariance matrix appropriately merging the statistical and systematic uncertainties provided by the BESIII Collaboration together with its spectrum, and paying special care to the normalization uncertainty treatment. This should be done by closely following the information provided together with its spectrum by the Collaboration.^25^

For definiteness, the data point of the BESIII sample [24] at the energy squared $s_i$ is:

$$\begin{aligned} m_i \pm \sigma _{\mathrm{stat},i} \pm \sigma _{\mathrm{syst},i}, \end{aligned}$$

using obvious notations; it is useful to define $\sigma (s_i)=\sigma _{\mathrm{stat},i}/m_i$, the $i{\mathrm{th}}$ experimental fractional systematic error. Then, the elements of the full covariance matrix *W* associated with the BESIII spectrum are written as:

50 $$\begin{aligned} W_{ij}=V_{ij} + \sigma (s_i) \sigma (s_j) A_i A_j , \end{aligned}$$

where the indices run over the number of data points ($i,j= 1, \cdots N$). *V* is the (diagonal) matrix of the squared statistical errors ($\sigma _{stat,i}^2$), and $\sigma (s_i)$ is the reported fractional systematic error at the data point of energy (squared) $s_i$, defined as just above. The systematic errors are considered point-to-point correlated and reflecting a (global) normalization uncertainty.

At the start of the fit iterative procedure, the natural choice for *A* is the vector of measurements itself ($A_i=m_i$); in the iterations afterwards, it is highly recommended [73–75] to replace the measurements by the fitting model function *M* ($A_i=M(s_i) \equiv M_i$) derived at the previous iteration step in order to avoid the occurrence of biases. Then, the experiment contribution to the global $\chi ^2$ is written as:

51 $$\begin{aligned} \chi ^2 = (m - M)_i W^{-1}_{ij} (m - M)_j . \end{aligned}$$

Moreover, a normalization correction naturally follows from the global scale uncertainty. It is a *derived* quantity of the minimization procedure. Defining the vector *B* ($B_i=\sigma (s_i) M_i$), this correction is given by [19, 75]:

52 $$\begin{aligned} \displaystyle \mu = \frac{B_i V^{-1}_{ij} (m - M)_j }{1+B_i V^{-1}_{ij} B_j}. \end{aligned}$$

For the purpose of graphical representation, one could either perform the replacement $m_i \rightarrow m_i - \mu \sigma (s_i) M_i$, *or* apply the correction to the model function $M_i \rightarrow [1+ \mu \sigma (s_i)] M_i$. When graphically comparing several spectra, the former option should clearly be preferred, as indeed, even if not submitted to the fit, other parent data samples can be fruitfully represented in the same plot by performing the change^26^:

$$\begin{aligned} m^\prime _i \rightarrow m^\prime _i - \mu \sigma ^\prime _i(s^\prime _i) M(s^\prime _i), \end{aligned}$$

using the BESIII fit function *M*(*s*). Such a plot is obviously a relevant visual piece of information.

A global fit involving all data covering the $\pi \pi$, $(\pi ^0/\eta ) \gamma$ and both $K {\overline{K}}$ channels and *only* the BESIII spectrum to cover the 3$\pi$ annihilation final state has been performed and returns, for the BESIII sample,^27^$\chi ^2/N= 170/128$.

**Fig. 3:**
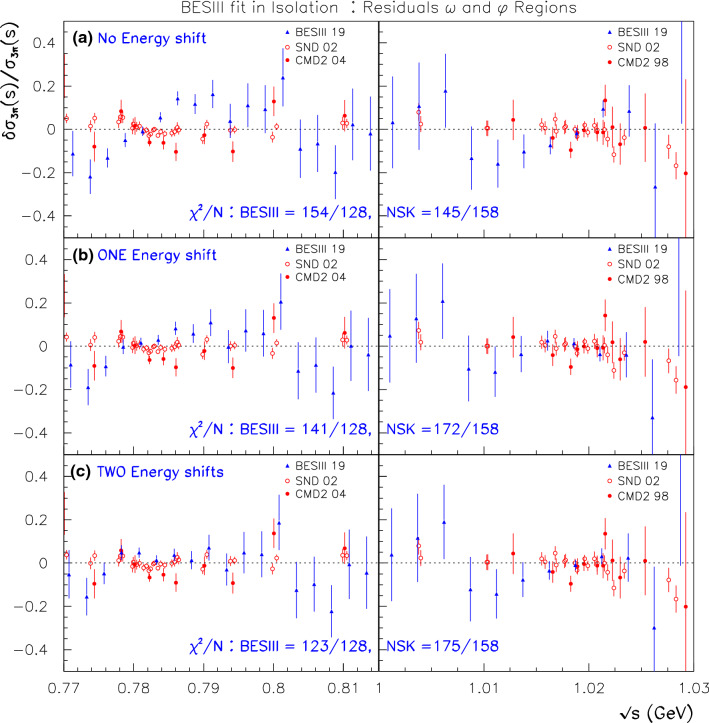
Normalized residuals of $\hbox {EBHLS}_2$ fits to the BESIII $3 \pi$ data in isolation under three different configurations: no energy shift (top panels), one global energy shift (middle panels) and two energy shifts (bottom panels). The normalized residuals are defined as $\delta \sigma (s)/\sigma (s)$, where $\delta \sigma (s_i) = m^\prime _i- M(s_i)$ – see the text for the definitions. The partial $\chi ^2/N$’s are displayed. Also shown, the NSK $\chi ^2/N$’s distance of the CMD2 and SND data to the best fit solutions derived from fitting BESIII data in isolation in each configuration

The top panels in Fig. 3 display the distribution of the BESIII normalized residuals $\delta \sigma (s)/\sigma (s)$ corrected as noted just above. In the $\omega$ region, at least, the normalized residual distribution is clearly energy-dependent. The normalized (pseudo-)residuals of the *unfitted* data samples displayed,^28^ namely those from [42, 66] in the $\omega$ region and from [63, 67] in the $\phi$ region, likewise corrected for the normalization uncertainty, are, instead, satisfactory,^29^ despite being unfitted. The fit process allows us to compute the (global) $\chi ^2$ distance of the NSK samples to the (BESIII) fit function and returns $\chi ^2/N=180/158$, a reasonable value for unfitted data.

However, the behavior of the BESIII residuals may indicate a mismatch between the $\omega$ and $\phi$ pole positions in the BESIII sample compared to the other ($\simeq 50$) data samples involved in the (global) fit; indeed, the narrow $\omega$ and $\phi$ signals are already present in the $(\pi ^0/\eta ) \gamma$ and $K {\overline{K}}$ channels, and therefore, as when dealing with the CMD3 and BaBar dikaon samples in [19], a mass recalibration (shift) could be necessary to avoid mismatches with the pole positions for $\omega$ and $\phi$ in the other data samples. We thus have refitted the BESIII data, by allowing for such a mass shift to recalibrate the BESIII energies and match our reference energy scale.^30^ So, we define:

$$\begin{aligned} E_\mathrm{BESIII} = E_\mathrm{NSK} + \delta E_\mathrm{BESIII} \end{aligned}$$

and let $\delta E_\mathrm{BESIII}$ vary within the fit procedure. The fit returns $\delta E_\mathrm{BESIII}= (-286.09 \pm 44.19)$ keV with $\chi ^2/N_\mathrm{BESIII}= 141/128$, and thus the (noticeable) gain of 29 units should be attributed to only allowing for a nonzero $\delta E_\mathrm{BESIII}$. The corresponding normalized residuals, displayed in the middle row of Fig. 3, are clearly much improved, whereas the $\chi ^2$ distance of the NSK 3$\pi$ data sample to the BESIII fit function stays the same.

Owing to the sharp improvement produced by this mass shift, it was tempting to check whether the energy (re-)calibration could be somewhat different at the $\omega$ and the $\phi$ masses. For this purpose, it is appropriate to redefine the fitting algorithm by stating:

53 $$\begin{aligned} \displaystyle E_\mathrm{BESIII} = E_\mathrm{NSK} + \left\{ \begin{array}{lll} \delta E_\mathrm{BESIII}^\omega , &{} E_\mathrm{BESIII} < E_\mathrm{mid} \\ \delta E_\mathrm{BESIII}^\phi , &{} E_\mathrm{BESIII} > E_\mathrm{mid} \end{array} \right. \end{aligned}$$

where $E_\mathrm{mid}$ should be chosen appropriately, i.e. significantly outside the $\omega$ and $\phi$ peak energy intervals. As obvious from the bottom panel of Fig. 5, the 3$\pi$ cross section in the intermediate energy region is almost flat and indicates that the choice for $E_\mathrm{mid}$ is far from critical; we chose $E_\mathrm{mid}=0.93$ GeV.

The corresponding global fit has been performed and returns $\chi ^2/N_\mathrm{BESIII}= 123/128$, with an additional gain of 18 $\chi ^2$ units, to be added to the previous 29-unit gain. The recalibration constants versus $E_\mathrm{NSK}$ are:

$$\begin{aligned}&\Big \{\delta E_\mathrm{BESIII}^\omega =(-518.92 \pm 72.04)~\mathrm{keV}, \\&\quad \delta E_\mathrm{BESIII}^\phi =(-118.58 \pm 58.72)~\mathrm{keV} \Big \}. \end{aligned}$$

After this recalibration has been applied, the BESIII normalized residuals, shown in the panels of the bottom row in Fig. 3, are observed to be flat, as well as their NSK partners also displayed.

We should note that $\delta E_\mathrm{BESIII}^\omega$ is in striking correspondence with the central value for the energy shift reported by BESIII [24] compared to their Monte Carlo ($-0.53 \pm 0.25$ MeV) and is found highly significant (about $7.5 \sigma$). $\delta E_\mathrm{BESIII}^\phi$ is also consistent with this number but quite significantly different from $\delta E_\mathrm{BESIII}^\omega$. Actually, comparing the three rightmost panels in Fig. 3, one observes that the main gain of decorrelating the energy calibration at the $\omega$ and $\phi$ peaks widely improves the former energy region; the latter looks almost insensitive, as reflected by the fact that the nonzero $\delta E_\mathrm{BESIII}^\phi$ is only a $2\sigma$ effect.

So, once two energy recalibrations have been performed, the description of the BESIII sample is quite satisfactory and, fitted with the other annihilation channels, the $\chi ^2$ probability is comfortable (91.6%).

At first sight, the differing energy shifts just reported may look surprising as, for ISR spectra, the energy calibration is very precisely fixed by the energy at which the accelerator is running at meson factories. However, such energy shifts could be related to unaccounted effects of the secondary photon emission expected to affect the resonances showing up at lower energies. In the case of the BESIII spectrum, this concerns the $\phi$ and $\omega$ regions, where photon radiation effects are enhanced by the resonances, causing shifts between the physical resonance parameters and their observed partners.^31^ This topic is specifically addressed in Appendix D, where it is shown – and illustrated by Table 14 therein – that the expected shifts produced by secondary ISR photons are in striking correspondence with the fitted $\delta E_\mathrm{BESIII}^\omega$ and $\delta E_\mathrm{BESIII}^\phi$.

### Exploratory $\hbox {EBHLS}_2$ global fits including the BESIII 3$\pi$ sample

**Fig. 4:**
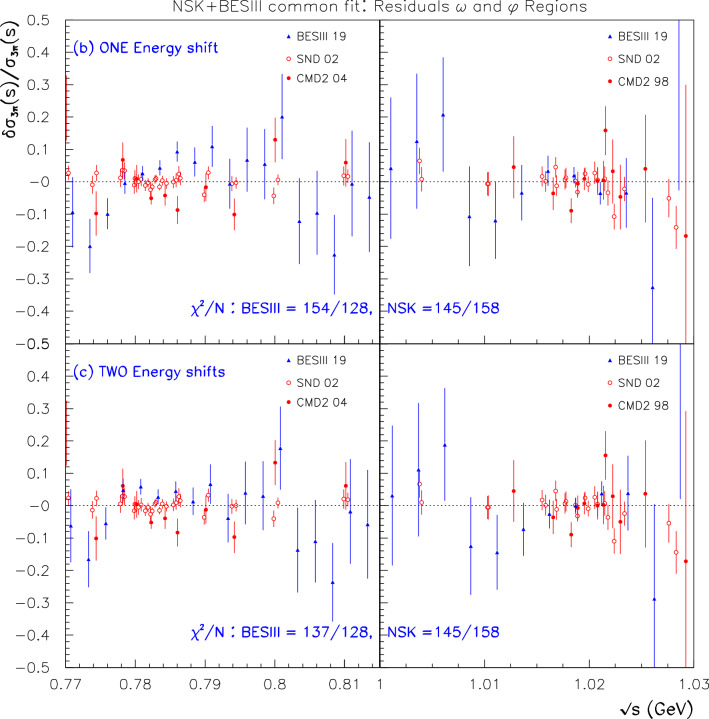
Normalized residuals of $\hbox {EBHLS}_2$ fits to the BESIII, CMD2 and SND $3 \pi$ data under two different configurations: top panels correspond to a (global) fit with only one energy shift for the BESIII spectrum; the bottom ones are derived allowing different $\delta E_\mathrm{BESIII}^\omega$ and $\delta E_\mathrm{BESIII}^\phi$. The partial $\chi ^2/N$ are displayed for the BESIII sample on the one hand, and for the CMD2 and SND ones (NSK) on the other hand

Having proved that the BESIII 3$\pi$ data sample suitably fits the global $\hbox {EBHLS}_2$ framework, we perform the analysis by merging the BESIII and the parent CMD2 and SND data samples within a common fit procedure. For completeness, we have first performed a global fit allowing for a single energy calibration constant. The fit returns $\chi ^2/N=1284/1365$ and 84.7 % probability. The $\chi ^2/N$ values for BESIII (154/128) and NSK (145/158) are also reasonable; however, the normalized residuals for BESIII shown in the top panels of Fig. 4 – especially the leftmost panel – still exhibit a structured behavior.

Therefore, we have allowed for two independent energy shifts $\delta E_\mathrm{BESIII}^\omega$ and $\delta E_\mathrm{BESIII}^\phi$ within the iterative fit procedure. Convergence is reached with $\chi ^2/N=1267/1365$ and 91.2% probability. The $\chi ^2/N$ value for BESIII (137/128) is improved by 17 units, whereas it is unchanged for NSK (147/158); thus the improvement of the total $\chi ^2$ only proceeds from the 17-unit reduction of the BESIII partial $\chi ^2$. The bottom panels in Fig. 4 are indeed observed flat in the $\omega$ and $\phi$ regions (this last distribution is still less sensitive to the fit quality improvement). The energy recalibration constants of the BESIII data with regard to the NSK energy scale are:

$$\begin{aligned} \{\delta E_\mathrm{BESIII}^\omega= & {} (-486.11 \pm 71.51)~\mathrm{keV},\\ \delta E_\mathrm{BESIII}^\phi= & {} (-135.31 \pm 59.16)~\mathrm{keV} \}, \end{aligned}$$

in fair accord with those derived in the global fit performed discarding the NSK 3$\pi$ data. Compared to its fit in isolation, the BESIII data $\chi ^2$ is degraded by $137 - 123 = 14$ units, while (see Table 4 third data column) the NSK data $\chi ^2$ is degraded by $147 - 135 = 12$ units compared to the fit performed discarding the BESIII data sample, the rest being unchanged. Regarding the average per data point, the degradation is of the order 0.1 $\chi ^2$-unit for both the NSK and BESIII samples, a quite insignificant change. So, one can conclude that the full set of consistent data samples can welcome the BESIII sample [24], once the energy shifts $\delta E_\mathrm{BESIII}^\omega$ and $\delta E_\mathrm{BESIII}^\phi$ are applied.

**Fig. 5:**
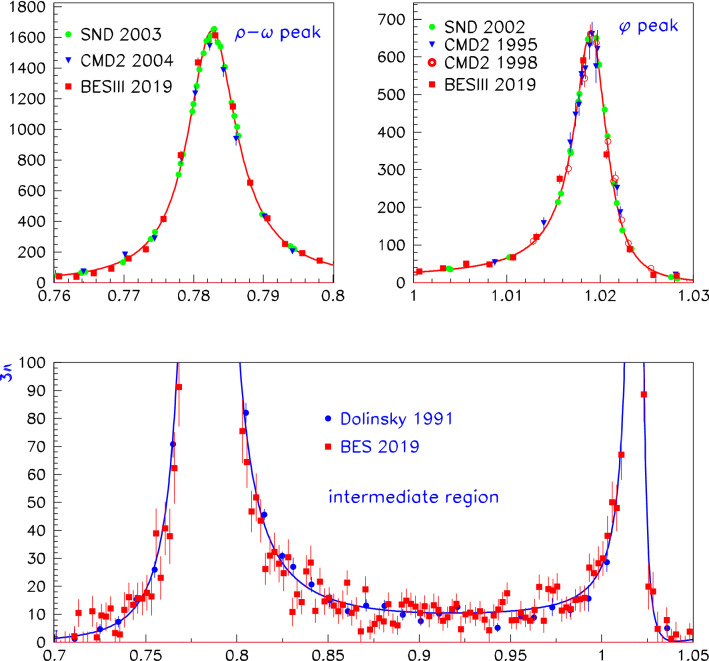
The global $\hbox {EBHSL}_2$ fit with the $\pi ^+ \pi ^-\pi ^0$ spectra, corrected for their normalization uncertainty; only statistical errors are shown. The top panels display the data and fit in the $\omega$ and $\phi$ mass intervals; the bottom panel focuses on the intermediate energy region. The energy recalibration has been applied to the BESIII data

Figure 5 displays the fit function and data in the 3$\pi$ channel. All data are normalization-corrected as emphasized above, and additionally, the energy shifts induced by having different $\delta E_\mathrm{BESIII}^\omega$ and $\delta E_\mathrm{BESIII}^\phi$ calibration constants are applied to the BESIII data sample; one should also note the nice matching of the ND and BESIII data in the intermediate region. Additional fit details of this new global fit are given in the third data column of Table 4.

## Revisiting the $2 \pi$ annihilation channel

A fair understanding of the dipion annihilation channel, which provides by far the largest contribution ($\simeq 75\%$) to the muon HVP, is an important issue. Fortunately, the $e^+ e^- \rightarrow \pi ^+ \pi ^-$ cross section is also the most important channel encompassed within the BHLS [14, 75] and $\hbox {BHLS}_2$ [19] frameworks developed previously. All the existing dipion data samples were examined within the context of these two variants of the HLS model. As some of them exhibit strong tensions [14] with significant effects on the derived physics quantities, it looks worthwhile to revisit this issue when a new measurement arises, at least to check whether the consistency pattern previously favored deserves reexamination.

Besides the data samples formerly collected and gathered in [76], an important place should be devoted to the data from CMD-2 [43, 44, 77] and SND [45] collected in scan mode on the VEPP-2M collider at Novosibirsk; these CMD2 and SND samples are collectively referred to below as NSK. These were followed by higher statistics samples, namely the KLOE08 spectrum [28] collected at Da$\Phi$ne and those collected by BaBar [78] at PEP-II, both using the initial-state radiation (ISR) method [79]. Slightly later, the KLOE Collaboration produced two more ISR data samples, KLOE10 [46] and KLOE12 [47], the latter being tightly related to KLOE08 (see Fig. 1 in [80]). In this reference, the KLOE-2 Collaboration has also published a dipion spectrum derived by combining the KLOE08, KLOE10 and KLOE12 spectra; this combined spectrum is referred to below as KLOE85, thus named according to its number of data points.

Two more data samples, also collected in the ISR mode, were appended to this list by BESIII [26] – with recently improved statistical errors [27] – and a CLEO-c group [48]. Finally, the SND collaboration has just published a data sample [25] collected in scan mode on the new VEPP-2000 facility at Novosibirsk; this spectrum, seemingly still preliminary, is referred to below as SND20. Another high statistics data sample, also collected in scan mode, is expected from the CMD3 Collaboration [81].

Important tension between some of these samples – namely KLOE08 and BaBar – and all others have already been identified [2, 75]; the occurrence of the new data sample from SND (and its comparison with NSK, KLOE and BaBar [25]) allows us to reexamine this consistency issue and provides the opportunity to remind the reader how it is dealt with within global frameworks.

### The sample analysis method: a brief reminder

The broken HLS modelings previously developed, especially $\hbox {BHLS}_2$ as well as its present extension, aim at providing frameworks which encompass a large part of the low-energy physics, the realm of the non-perturbative regime of QCD, and extend up to the $\phi$ mass region; they have rendered possible fair simultaneous accounts of the six major $e^+ e^-$ annihilation channels ($\pi ^+ \pi ^-$, $\pi ^+ \pi ^- \pi ^0$, $K^+ K^-$, $K_L K_S$, $(\pi ^0/\eta ) \gamma$) up to 1.05 GeV/c; slightly modified ($\hbox {EBHLS}_2$), this framework also now provides a satisfactory understanding of the A, B and C dipion spectra from the decay of the $\tau$ lepton.

As already noted several times, the Lagrangians which substantiate the various broken HLS models emphasize a property expected from QCD: the different annihilation channels should be correlated via their common underlying QCD background; this is reflected within our effective Lagrangians by the fact that all their model parameters are simultaneously involved in the amplitudes for any of the accessible physics processes they encompass. A straightforward example is represented by *g*, the universal vector coupling, and this property is, more generally, exhibited by the expressions for the various amplitudes derived from within the various broken HLS Lagrangians.

Most of the Lagrangian parameters are not known ab initio and are derived from the data via a global fit involving all channels and, possibly, all available data samples. For this purpose, the provided data samples and associated information (data points, statistical errors, systematics, correlated or not) are supposed reasonably^32^ well estimated. With this at hand, one can construct a motivated global $\chi ^2$ and derive the Lagrangian parameters through a minimization procedure like minuit.

Among the various kinds of uncertainties reported by the different experiments, special care should be devoted to the global normalization uncertainties – which can be energy-dependent, as already dealt with in Sect. 8.1. Actually, as for energy scale recalibrations (see also Sect. 8.1), it looks obvious that the most appropriate normalization of a given sample can only be determined by comparing with several other independent spectra covering the same physics channel. Even more, a global treatment of these provides the best normalization of each sample versus all the others by a kind of bootstrap mechanism.

Actually, a global fit, when possible, appears to be the best tool to determine the most appropriate normalization of each spectrum in accord with its reported uncertainties, including its normalization uncertainties; this is noted in detail in [19, 75] and above in Sect. 8. The goodness of the corresponding fit indicates the confidence one can devote to the normalization corrections.

The probability of the best fit reflects the quality of the experimental information and the relative consistency of the various data samples involved in the procedure within the model framework; we now have three significantly different HLS frameworks at hand which have been shown in [19] and just above to lead to a consistent picture.

### Samples covering the $\pi ^+ \pi ^-$ channel: a few properties

Let us first consider the data samples already identified as not exhibiting significant tension among them within the $\hbox {BHLS}_2$ frameworks, the previous one [19] or the present one; this defines a reference set of data samples, named $\mathcal{H}_R$. This covers the $3\pi$ data samples already considered in Sect. 8 and all the existing data samples covering the $\pi ^0 \gamma$ and $\eta \gamma$ decay channels. Regarding the dikaon spectra, we refer the reader to our analysis in [19], where the tensions between the CMD3 spectra [71, 72] and the others from SND, BaBar and (corrected [19]) CMD2 led us to discard them from the analysis.^33^ As for $\tau$ dipion spectra, it was shown in Sect. 4 that the residual tension observed in the account for Belle compared to Aleph and Cleo can be absorbed. The reference set $\mathcal{H}_R$ also includes the spacelike pion [54, 55] and kaon form factor spectra [82, 83] which are satisfactorily understood within the $\hbox {BHLS}_2$ frameworks [19].

For the purpose of reexamining sample tensions, it seems appropriate to also include in $\mathcal{H}_R$, the pion form factor spectra collected by BESIII [26, 27] and Cleo-c [48]. Indeed, anticipating somewhat our fit results, it has been observed that, alone or together with either of the NSK, KLOE, BaBar samples, or with any combination of these, they get the same individual sample $\chi ^2$’s, with fluctuations not exceeding 1.5 units for each of them. Fitting the $\mathcal{H}_R$ sample set thus defined within the present framework returns $\chi ^2/N = 926/1021$ and a 94% probability; in this fit, the BESIII and Cleo-c samples yield

$$\begin{aligned} {[}\chi ^2/N]_\mathrm{BESIII} = 49/60,\quad [\chi ^2/N]_\mathrm{Cleo-c}=27/35. \end{aligned}$$

One can consider the probability of the global fit (here 94%) as a faithful tag of mutual consistency of the (more than 50) samples included in $\mathcal{H}_R$ which fairly fit the broken HLS framework.

**Table 3:** Properties of the global fits performed with the present upgraded $\hbox {BHLS}_2$ model using the $\mathcal{H}_R$ sample collection with one among the NSK, KLOE and BaBar samples and with pairs of these. The table is organized such that the first line displays the value for $\chi ^2/N_\mathrm{NSK}$ returned by fitting the three configurations ($\mathcal{H}_R$ + NSK), ($\mathcal{H}_R$ + NSK + KLOE), ($\mathcal{H}_R$ + NSK + BaBar); the corresponding fit probabilities are shown within square brackets. The second and third lines display the similar information for KLOE and BaBar. The number of data points in each of NSK, KLOE and BaBar is shown in the first column for convenience

$\chi ^2/N_{\mathrm{pts}}$ [Prob]	+ NSK	+ KLOE	+ BaBar
NSK $\pi ^+\pi ^-$ (127)	129/127 [95.3%]	142/127 [91.2%]	138/127 [51.7%]
KLOE $\pi ^+\pi ^-$ (135)	136/135 [91.2%]	132/135 [95.2%]	148/135 [31.9%]
BaBar $\pi ^+\pi ^-$ (270)	328/270 [51.7%]	354/270 [31.9%]	326/270 [62.9%]

We have made two kinds of global fits:

- (i) Fits involving $\mathcal{H}_R$ and each of NSK, KLOE^34^ and BaBar in turn; the diagonal in Table 3 reports the main results, namely the value returned for $\chi ^2/N$ of resp. NSK, KLOE, BaBar and the probability of the global fit at the corresponding table entry.
- (ii) Fits involving $\mathcal{H}_R$ and the pairwise combinations (NSK, KLOE), (NSK, BaBar) and (KLOE, BaBar) in turn; the main fit results are reported in the non-diagonal entries of Table 3. In order to simplify the comparison of each of the NSK, KLOE, BaBar accounts provided by these “pairwise” fits, we have organized the non-diagonal entries in a specific manner: the entry (NSK, KLOE) provides $[\chi ^2/N]_\mathrm{NSK}$ and the global fit probability, whereas the entry (KLOE, NSK) provides $[\chi ^2/N]_\mathrm{KLOE}$ and the (same) fit probability. The same rule applies *mutatis mutandis* to the other pairwise fits: (NSK, BaBar) and (KLOE, BaBar) together with $\mathcal{H}_R$.

Relying on Table 3, one clearly observes that the single-mode fits for NSK and KLOE are fairly good and in nice accord with the results returned by the corresponding pairwise fit. The pattern is somewhat different when BaBar is involved.

In order to be complete, let us briefly summarize the fit results obtained within the present framework concerning KLOE08 and the KLOE85 sample derived by the KLOE2 Collaboration from their combination of the KLOE08, KLOE10 and KLOE12 spectra [80].

- Regarding KLOE08: The global fit for $\mathcal{H}_R$ + KLOE08 returns $\chi ^2/N_\mathrm{KLOE08}=95/60$ and a 74.7% global fit probability. With an average $<\chi ^2> \simeq 1.5$, one does not confidently consider the results derived from the fit to this combination compared to KLOE10 + KLOE12.
- Regarding KLOE85: The fit for $\mathcal{H}_R$ + KLOE85 returns $\chi ^2/N_\mathrm{KLOE85}=83/85$ (global fit probability 94.7%) which clearly indicates that the KLOE08 issue is reasonably well dealt with in the KLOE85 combination [80]. We have also performed the pairwise fit $\mathcal{H}_R$ + KLOE85 + NSK. In this case, we get:
  $$\begin{aligned}{}[\chi ^2/N]_\mathrm{NSK} = 160/127,\quad [\chi ^2/N]_\mathrm{KLOE85}=93/85, \end{aligned}$$
  with an 80.7% probability. This fit is obviously reasonable^35^ but less satisfactory than $\mathcal{H}_R$ + NSK + KLOE, as the tension between KLOE85 and NSK is large, much larger than when using $\mathcal{H}_R$ +NSK + KLOE as displayed in Table 3.

### The case for the 2020 SND dipion sample: fits in isolation

**Fig. 6:**
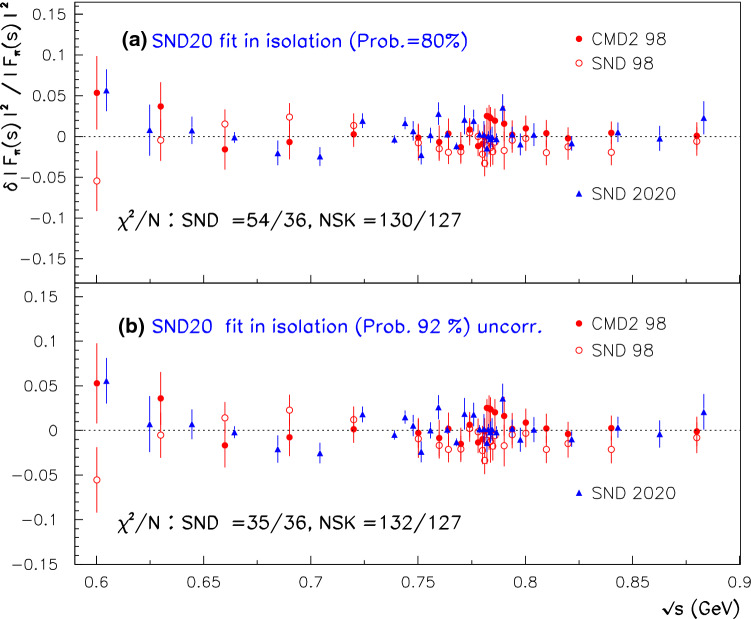
Fit of the SND20 data [25] in isolation within the $\hbox {BHLS}_2$ framework. The top panel displays the results corresponding to a fit where SND20 systematics are fully point-to-point correlated, whereas the bottom panel is obtained by treating the SND20 systematics as fully uncorrelated. The NSK spectra are displayed but not fitted. See text for further explanation

In order to analyze the new data sample recently provided by the SND Collaboration [25], the treatment of the reported systematic errors has been performed as emphasized above for the $3\pi$ data from BESIII (see Sect. 8.1), as the systematics are expected to be fully point-to-point correlated.^36^ For the present analysis, we have first performed global fits^37^ where the single representative for the $e^+ e^- \rightarrow \pi ^+ \pi ^-$ annihilation channel is SND20, the new SND data sample [25]; the spacelike pion form factor data [54, 55] have also been discarded from the fits in isolation. Figure 6 summarizes our results.

The top panel in Fig. 6 indicates that, in single mode, the best fit returns a reasonable probability. However, this comes with a large average $<\chi ^2>_{SND20}=54/36=1.5$ (to be compared with the diagonal in Table 3). Nevertheless, amazingly, the SND20 form factor derived from this global fit provides a fairly good account of the NSK (CMD2 and SND) data *not submitted to the fit* as one yields $<\chi ^2>_\mathrm{NSK}=130/127=1.02$, much better than SND20 itself. The NSK (pseudo-)residual spectrum is consistent with flatness, and additionally, the ratio 130/127 indicates that there is no significant energy calibration mismatch between the NSK samples and SND20 – this may have shown up in the $\rho ^0-\omega$ drop-off region.

The bottom panel in Fig. 6 displays results derived by assuming the SND20 systematics fully uncorrelated (i.e. the non-diagonal elements of the error covariance matrix are dropped out). The global fit is successful and returns a 92% probability. The gain for SND20 is noticeable as $<\chi ^2>_\mathrm{SND20}=35/36=0.97$, and clearly, the (alternative) pion form factor derived by fitting only the SND20 data (in this manner) within the global framework is almost unchanged; this is the way the $\chi ^2$ distance of the NSK samples to this alternative fit form factor can be understood: $<\chi ^2>_\mathrm{NSK}=132/127=1.04$. Moreover, once again, the NSK (pseudo)residual distributions are as flat as (and almost identical to) those displayed in the top panel of Fig. 6.

We got substantially the same results and conclusions by enlarging the SND20 pion form factor statistical errors^38^ by 0.04 and keeping unchanged the systematics – i.e. treated as point-to-point correlated; this also points towards the interest in having, besides information on correlations, information on the accuracy of the uncertainties [2]. So, the way the SND20 uncertainties should be understood deserves clarification.

**Fig. 7:**
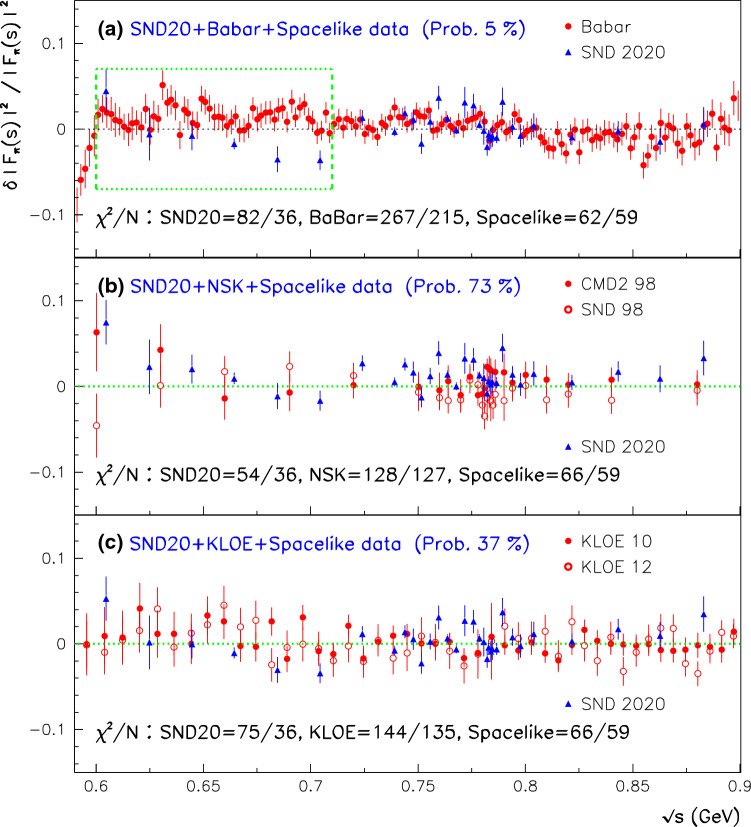
Fits of the SND20 dipion data [25] together with the spacelike data [54, 55]. The top panel shows the fit residuals when the timelike dipion channel is covered by the SND20 and BaBar [78] samples; similarly, the middle panel displays the fit residuals when covering the timelike dipion channel by the SND20 [25] and NSK [43–45, 77] spectra; the bottom panel reports likewise the case when the timelike dipion channel is covered by the SND20 and KLOE [46, 47] samples. All reported systematics are treated as point-to-point correlated

### The case for the 2020 SND dipion sample: pairwise fits

An interesting topic addressed in [25] is the consistency of SND20 with NSK (e.g. CMD2 [43, 44, 77] and SND-98 [45]), KLOE ( KLOE10 [46] and KLOE12 [47]) and BaBar [78]. For this purpose, it looks worthwhile to perform global fits by including pairwise combination to cover the $\pi ^+ \pi ^-$ annihilation channel.^39^ This allows us to observe the tension between the partners in the pair and to get a probability which emphasizes their global consistency. Our main fit results are collected in Fig. 7.

The middle panel in Fig. 7 shows the case for the global fit with the (SND20 + NSK) combination. As could be expected, this confirms the fit of SND20 in isolation reported in the top panel of Fig. 6: $<\chi _\mathrm{NSK}>$ is negligibly improved whereas $<\chi _\mathrm{SND20}>$ is unchanged; the large value for $<\chi _\mathrm{SND20}>=1.5$ is responsible for the global fit probability reduction compared to fits with NSK alone (or combined with KLOE), as can be seen in Table 3. With this proviso, $\hbox {BHLS}_2$ confirms the statement that SND20 and NSK are consistent [25] with a 73% probability.

**Fig. 8:**
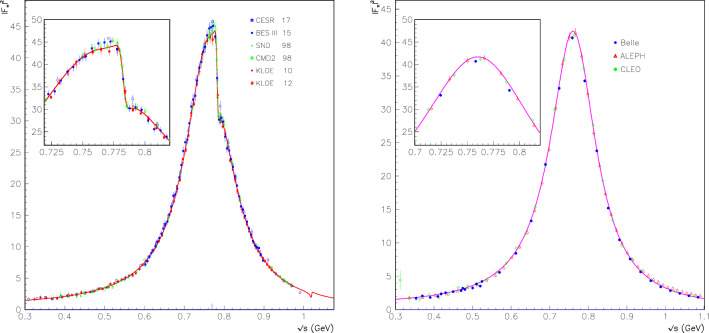
$\hbox {BHLS}_2$ fit to the $\pi \pi$ data, the upgraded BS solution: the left-hand panel shows the pion form factor squared in the $e^+e^-$ annihilation, and the right-hand one displays the same spectrum in the $\tau$ decay. The fitted regions extend up to $s=1.0$ $\hbox {GeV}^2$

In order to address the consistency topic about SND20 and BaBar already studied in [25], we have run our global fit procedure with the (SND20 + BaBar) combination. To stay as close as possible to the study reported in [25], we have found it worthwhile to exclude from the fit the part of the BaBar spectrum with $\sqrt{s} \in [0.60,~0.71]$. The results are displayed in the top panel of Fig. 7 and show some resemblance between SND20 and BaBar (normalized) residuals outside the BaBar excluded region (delimited by the green rectangle). Nevertheless, the fit probability is poor, and its $<\chi _\mathrm{SND20}>=82/36=2.3$ indicates a significant tension compared to the fit in isolation ($<\chi _\mathrm{SND20}>=1.5$)

Finally, the bottom panel in Fig. 7, reports the main fit results obtained by fitting the (SND20 + KLOE) combination. One can note the results compared to the fits in isolation: $<\,\chi _\mathrm{SND20}>=2.1$ (versus $<\chi _\mathrm{SND20}>=1.5$) while $<\chi _\mathrm{KLOE}>=144/135=1.1$ (versus $<\chi _\mathrm{KLOE}>=0.98$) and a 37% probability. Here also, one can observe that the residual distributions for SND20 are not really at variance with those for KLOE along the whole energy range.

So, on the whole, the SND20 spectrum [25] does not help in clarifying the consistency issue raised by the existing dipion spectra, and presently, SND20 does not bring in more support, or less support, to the choice performed following our previous analyses (see Tables 3 and 4) and illustrated by Fig. 8.

## Overview of the $\hbox {EBHLS}_2$ fits

**Table 4:** Global fit properties of the $\hbox {EBHLS}_2$ fits ; second line in the table title indicates the running conditions regarding the data samples submitted to fit or the running of the BS or RS variants when $\lambda _3 \ne 0$. The number of data points involved is given between parentheses in the first column. The last lines display the global $\chi ^2/N_{\mathrm{pts}}$ and probability of each fit

$\hbox {EBHLS}_2$	BS ($\lambda _3=0$)		($\lambda _3 \ne 0$)	
	Excl. $\tau$	Incl. $\tau$	BS	RS
NSK $\pi ^+ \pi ^-$ (127)	136	134	138	136
KLOE $\pi ^+ \pi ^-$ (135)	141	146	139	139
BESIII $\pi ^+ \pi ^-$ (60)	48	47	49	48
Spacelike $\pi ^+ \pi ^-$ (59)	62	67	62	60
$\tau$ (ABC) (84)	$\times$	93	82	80
$\pi ^0 \gamma$ (112)	89	88	88	87
$\eta \gamma$ (182)	120	120	124	124
NSK $\pi ^+ \pi ^-\pi ^0$ (158)	142	146	147	147
BESIII $\pi ^+ \pi ^-\pi ^0$ (128)	138	138	137	137
NSK $K_L K_S$ (92)	103	104	103	104
NSK $K^+ K^-$ (49)	41	42	39	39
BaBar $K^+ K^-$ (27)	41	42	41	41
Spacelike $K^+ K^-$ (25)	18	19	17	18
Decays (8)	5	4	9	9
$\chi ^2/N_{\mathrm{pts}}$	1179/1280	1285/1365	1269/1365	1262/1365
Probability	93.3%	83.1 %	90.0 %	91.5 %

Some of the general properties of the global fits performed in the $\hbox {EBHLS}_2$ framework have already been emphasized in Sects. 3, 8 and 9, with special emphasis on the $\tau$ decay dipion spectra, the $e^+ e^- \rightarrow \pi ^+ \pi ^- \pi ^0$ annihilation channel and the crucial $e^+ e^- \rightarrow \pi ^+ \pi ^-$ one, respectively. The general features of our fitting algorithm concept are detailed in Sect. 15 of our [19], for instance. Let us, for convenience, recall the gross features of our global fit method:

- The contribution of each data sample to the global $\chi ^2$ to be minimized is constructed using solely the uncertainties *exactly as they are provided by each experiment without any external input*. Additionally, this kind of input may influence the numerical outcome of the fits in an uncontrolled way. On the other hand, if not already performed by the relevant experiments, the reported uncorrelated systematics are merged appropriately with the statistical error covariance matrix.
- The correlated systematic errors – possibly *s*-dependent – are treated with special care [75] as emphasized in Sect. 8.1 in order to avoid the so-called Peelle pertinent puzzle [84] which generally results in biasing the evaluation of physics quantities based on $\chi ^2$ minimization procedures [85, 86]. Moreover, an iterative procedure is used which has been proved to avoid biases [75]. In the case of a global $\chi ^2$ minimization, it should be stressed that the absolute scale of each experiment is derived in full consistency with those of all the other experiments (or data samples), especially – but not only – with those collected by other groups in the same physics channel.^40^ Several examples can be found in [19], where it is shown that the derived scale corrections compare quite well with the corresponding experimental expectations.
- When merging the different data samples which cover the same energy range, their different energy calibrations may exhibit some mismatch; this issue was previously encountered in our [19] with the energy calibration of the dikaon spectra from CMD-3 [71, 72] and BaBar [40] versus those of the corresponding samples from CMD2 and SND; this issue happened again herein when dealing with the BESIII 3$\pi$ sample [24] and is solved accordingly (see Sect. 8 above). It should be noted that, for signals as narrow as the $\omega$ and $\phi$ mesons, global fit techniques are certainly the best suited to match the energy scales of various spectra that are otherwise poorly consistent.
- In order to *confidently* rely on global fit outputs to evaluate physics quantities, one should discard data samples which exhibit noticeable inconsistencies with the rest of the benchmark samples. Our requirement to identify such samples has generally been to get an average $\chi ^2$ per point smaller than $\simeq 1.5$.

Compared to the fits reported in [19] and as already noted in Sect. 2, we have here released the constraint (Eq. (2)) on the product $\epsilon \epsilon ^\prime$ and also let the mixing angle $\theta _P$ float freely. Moreover, as several preliminary fits typically return:

54 $$\begin{aligned} \begin{array}{lll} \displaystyle \Delta _A=[0.55 \pm 4.59] \times 10^{-2},\quad \mathrm{and}~~~\\ \displaystyle \lambda _8=[2.18 \pm 4.18] \times 10^{-2}, \end{array} \end{aligned}$$

imposing $\Delta _A=\lambda _8=0$ looks worthwhile; indeed, Eq. (54) clearly shows that the physics presently addressed in the $\hbox {EBHLS}_2$ framework does not exhibit a significant sensitivity to these parameters when left free. These constraints will be reexamined^41^ in the context where the $[\pi ^0,\eta ,{\eta ^\prime }]$ mixing is also addressed.

Table 4 reports the fit results under four configurations; the energy scale corrections for the BaBar dikaons [40] and the BESIII samples [24] are floating parameters. The first two data columns, actually, update the $\hbox {BHLS}_2$ fit results derived for the BS variant given in [19]; for the fit performed including the $\tau$ spectra, the polynomial $\delta P^\tau (s)$ here is third degree.

For the $\hbox {EBHLS}_2$ fits reported in the last two data columns, $\delta P^\tau (s)$ is second degree.^42^ The third data column displays the $\chi ^2$ contributions of various groups of data samples to the global $\chi ^2$ using the BS configuration. For completeness, the last data column reports the $\hbox {EBHLS}_2$ fit results obtained under its RS configuration [19].

One should note that, substantially, these pure $\hbox {EBHLS}_2$ fits and the $\hbox {BHLS}_2$ fit excluding the $\tau$ spectra (first data column) exhibit similar and favorable $\chi ^2$ averages per point for all groups of data samples with the sole exception of the individual decay modes, which is doubled. In the $\hbox {EBHLS}_2$ BS or RS configurations, the single mode which significantly departs from $\chi ^2 \le (1.0 \div 1.2)$, is $\eta \rightarrow \gamma \gamma$, which returns 4, e.g. a $2 \sigma$ difference with the review of particle properties [49]. On the other hand, one may consider, in view of Table 4, that the BS variant of $\hbox {EBHLS}_2$ does not need to be improved by the primordial mixing mechanism introduced in [19] to construct the RS variant of $\hbox {BHLS}_2$.

The numerical values of the model parameters of the $\hbox {EBHLS}_2$/$\hbox {BHLS}_2$ framework will be examined and discussed in a wider context involving the treatment of the $[\pi ^0,\eta ,{\eta ^\prime }]$ mixing properties as well in Sect. 18.

## Evaluation of $a_\mu$, the muon HVP

As the previous BHLS releases [14, 19], $\hbox {EBHLS}_2$ encompasses the bulk of the low-energy $e^+ e^- \rightarrow \mathrm{hadrons}$ annihilations up to, and including, the $\phi$ mass region. Therefore, suitably taking into account the various kinds of uncertainties reported by the different experiments affecting the spectra they collected, a *fully* global fit is expected to lead to precise evaluations of the contributions to $a_\mu ^{HVP-LO}$ from the energy region $\sqrt{s} \le 1.05$ GeV. Within this approach, the specific contribution of the hadronic channel $H_i$ is obtained by means of the cross section $\sigma (H_i,s) \equiv \sigma (e^+ e^- \rightarrow H_i, s)$ with parameter values derived from the global fit performed within the $\hbox {EBHLS}_2$ framework:

55 $$\begin{aligned}&\displaystyle a_\mu (H_i) =\frac{1}{4 \pi ^3} \int _{s_{H_i}}^{s_{cut}} ds ~K(s) ~\sigma (H_i,s), \nonumber \\&H_i=\left\{ \pi ^+\pi ^-, \pi ^0 \gamma , \eta \gamma , \pi ^+\pi ^- \pi ^0, K^+ K^-, K_L K_S \right\} . \end{aligned}$$

*K*(*s*) [87, 88] is the usual kernel which enhances the weight of the threshold regions compared to the higher-energy regions of the $$H_i$$ spectrum; $$s_{H_i}$$ is the threshold of the $$ H_i$$ hadronic channel and $$\sqrt{s_\mathrm{cut}}=1.05$$ GeV is the validity limit, common to the different HLS frameworks.

### Remarks on the $\sqrt{s} \le 1.05$ GeV contribution to $a_\mu (\pi \pi )$

We find it of special concern to substantiate what supports the choices performed in our global fit approach in connection with the muon HVP outcome. For this purpose, the analysis of the $\pi ^+ \pi ^-$ channel properties within the (E)$\hbox {BHLS}_2$ framework is of special relevance. Global fits have been performed involving the data samples collected for all the HLS final states except for $\pi \pi$ and complemented in turn by each of the various KLOE samples already discussed in [19] to feed solely the $\pi ^+ \pi ^-$ channel.

**Table 5:** The $\pi ^+ \pi ^-$ contribution to the HVP-LO in the range [0.35,  0.85] $\hbox {GeV}^2$ in units of $10^{-10}$. The direct integration evaluations are taken from Fig. 6 in [80]. The (E)$\hbox {BHLS}_2$ evaluations are derived by fits as sketched in the text; the last data column displays relevant pieces of the fit information

$\pi ^+ \pi ^-$ Data sample	Direct integration [80]	$\hbox {BHLS}_2$	$\chi ^2_{\pi ^+ \pi ^-}/N_{\pi ^+ \pi ^-}$ (Prob.)
KLOE10	$376.0 \pm 3.4$	$375.04 \pm 2.35$	69/75 (78%)
KLOE12	$377.4 \pm 2.6$	$376.74 \pm 1.59$	59/60 (80%)
KLOE85	$377.5 \pm 2.2$	$377.17 \pm 0.89$	95/85 (65%)
KLOE08	$378.9 \pm 3.2$	$373.78 \pm 1.84$	130/60 (14%)

Table 5 – reprinted from Table 3 in [2] – clearly shows that the (E)$\hbox {BHLS}_2$ central values obviously correspond closely with those derived by directly integrating the data [80], except for KLOE08, which correlatedly exhibits a poor global fit probability; this substantiates the reason that one may prefer discarding poorly fitted data samples to avoid biases, possibly large. However, one should also note that its effect within the KLOE85 combination is much softer, as the KLOE85 fit probability (65%) remains comparable to those for KLOE10 and KLOE12, which are both higher and almost identical (78% and 80 %).

Table 5 also shows the important reduction of the uncertainties induced by the non-$\pi ^+ \pi ^-$ channels involved in the reported global fits; this reduction is, of course, amplified when including the other accepted $\pi ^+ \pi ^-$ samples in the fit procedure, as will be seen shortly. On the other hand, when the fit probability is poor, the values returned by the fits for the uncertainty and the central value should be handled with care.

Table 6 shows a breakdown of the contributions to $a_\mu (\pi \pi )$ from different energy intervals. The top lines display the results derived by other groups, namely CHS [89], DHMZ [90] and KNT [91], while the bottom lines show the $\hbox {EBHLS}_2$ outcome from fits performed under the various indicated configurations. The favored configuration, which corresponds to a good account of all the channels encompassed within the $\hbox {EBHLS}_2$ framework, is tagged “KLOE+X.” Nevertheless, in order to really compare the global fit method with [89–91], it is worth relying on the same set of experimental data. To this end, we have also run our code including the BaBar data sample within the set of $\pi ^+ \pi ^-$ fitted spectra so that the sample contents are similar in all the approaches discussed; nevertheless, in order to avoid the effects of energy calibration mismatch between the BaBar and KLOE spectra within the fit procedure, we have removed the BaBar $\rho ^0-\omega$ drop-off region from the fit. The corresponding results are given in Table 6 under the tag “KLOE+BaBar+X.”

**Table 6:** Breakdown of $10^{10} \times a_\mu [\pi \pi ]$ by energy intervals. The displayed data for CHS18, DHMZ19 and KNT19 are extracted from Table 6 in [2]. The $\hbox {EBHLS}_2$ fits are reported using BaBar and KLOE10/12, and the latter only together with the NSK, BESIII and Cleo-c dipion spectra, globally referred to as X. The data collected in the 1980s [76] are also part of X

$\sqrt{s}$ Interval (GeV)	$\sqrt{s} \le 0.6$	$0.6 \le \sqrt{s} \le 0.9$	$0.9 \le \sqrt{s} \le 1.0$	$\sqrt{s} \le 1.0$
CHS18 [89]	$110.1 \pm 0.9$	$369.6 \pm 1.7$	$15.3\pm 0.1$	$495.0\pm 2.6$
DHMZ19 [90]	$110.4\pm 0.4 \pm 0.5$	$371.5 \pm 1.5 \pm 2.3$	$15.5 \pm 0.1 \pm 0.2$	$497.4 \pm 1.8 \pm 3.1$
KNT19 [91]	$108.7 \pm 0.9$	$369.8 \pm 1.3$	$15.3 \pm 0.1$	$493.8 \pm 1.9$
KLOE + BaBar + X	$\chi ^2/N_{pts}$: BaBar = 1.45, KLOE = 1.15, NSK = 1.10			
Prob = 11.4%	$108.83\pm 0.09$	$369.06 \pm 0.62$	$15.36 \pm 0.38$	$493.19 \pm 0.73$
KLOE +X	$\chi ^2/N_{pts}$: KLOE = 1.03, NSK = 1.09			
Prob = 90.0% (incl. $\tau$)	$107.79\pm 0.12$	$366.76 \pm 0.73$	$15.16 \pm 0.42$	$489.70 \pm 0.84$
Prob = 93.3% (excl. $\tau$)	$107.67\pm 0.13$	$367.21 \pm 0.84$	$15.17 \pm 0.48$	$490.05 \pm 0.98$

Regarding the reported central values for $a_\mu [\pi \pi ]$, it is clear that CHS18, DHMZ19, KNT19 and the evaluation derived from the KLOE+BaBar+X fit are similar; nevertheless, we should point out the higher similarity of the KNT19 and $\hbox {EBHLS}_2$ (KLOE + BaBar + X) evaluations. Indeed, the difference between their central values are 0.1, 0.7 and 0.2 for the $\sqrt{s} \le 0.6$ GeV, 0.6 GeV $\le \sqrt{s} \le 0.9$ GeV and 0.9 GeV $\le \sqrt{s} \le 1.0$ GeV energy intervals, respectively. One may infer that this fair agreement is mostly due to the similar treatments of the correlated systematics in the BHLS approaches [75] and in the KNT dealings [91].

Because in global approaches the data collected in the non-$\pi ^+ \pi ^-$ channels are equivalent to having at our disposal an additional statistic in the $\pi ^+ \pi ^-$ channel, one expects smaller errors for the (E)$\hbox {BHLS}_2$ evaluations of $a_\mu [\pi \pi ]$; this is indeed what is observed for the $\sqrt{s} \le 0.6$ GeV and 0.6 GeV $\le \sqrt{s} \le 0.9$ GeV contributions to $a_\mu [\pi \pi ]$ but, surprisingly, not for the 0.9 GeV $\le \sqrt{s} \le 1.0$ GeV interval. Nevertheless, integrated up to 1.0 GeV, the contribution to $a_\mu [\pi \pi ]$ exhibits an uncertainty improved by a factor of $\simeq 2.5$ compared to the other approaches reported in Table 6.

This comparison proves that the observed central value differences between $\hbox {BHLS}_2$ and the others – especially KNT – are mostly due to having discarded BaBar (and KLOE08) and only marginally to the global fit method. Finally, the last two lines of Table 6 show the effect of including the $\tau$ data. The use of these generates an additional (modest) improvement of the uncertainties, as could be expected, and a marginal shift. The comfortable probabilities reached by the $\hbox {EBHLS}_2$(KLOE+X) fits should also be noted. As discussed in Sect. 10, they are reached without resorting to error information beyond what is provided by the various experiments such as error inflation factors, for instance.

As noted several times, the validity range of the HLS approaches to $e^+ e^-$ annihilations extends up to $\simeq 1.05$ GeV, thus including the $\phi$ mass region. However, the [1.0, 1.05] GeV energy interval of the dipion spectrum is poorly known; indeed, apart from the BaBar spectrum^43^ [29], the most recent information about this spectrum piece follows from the old SND results [92] which underlie the RPP [49] entries for the $\phi \rightarrow \pi \pi$ decay.

As clear from Fig. 8, in this mass region the spectrum is widely dominated by the tail of the $\rho$ resonance with, in addition, a tiny effect due to the narrow $\phi$ signal. A direct numerical estimate derived from the scarce data collected around the $\phi$ mass gives $a_\mu (\pi \pi , [1.0,1.05]~\mathrm{GeV})=[3.35 \pm 0.04] \times 10^{-10}$. On the other hand, relying on the RPP [49] information, $\hbox {EBHLS}_2$ returns:

$$\begin{aligned} a_\mu (\pi \pi , [1.0,1.05]~\mathrm{GeV})=[3.07 \pm 0.11] \times 10^{-10}; \end{aligned}$$

replacing within the data set fitted via $\hbox {EBHLS}_2$ the RPP $\phi \rightarrow \pi \pi$ datum by the BaBar [1.0,1.05] GeV spectrum piece returns $a_\mu (\pi \pi , [1.0,1.05]~\mathrm{GeV})=[3.10 \pm 0.10] \times 10^{-10}$. Therefore, some (mild) systematics affect this mass region, as the cross section lineshape is not really well defined (see Fig. 1 in [15]).

### Contribution to the muon HVP of the energy region $\le 1.05$ GeV

The sum $a_\mu (HLS) = \sum _i a_\mu (H_i)$ of the quantities defined by Eq. (55) represents about 83% of the total muon HVP; it can be computed with fair precision using the $\hbox {EBHLS}_2$ fit information to construct the relevant cross sections; these are derived by sampling the model parameters using the parameter central values and the error covariance matrix returned by the minuit minimization procedure. Sampling out the model parameters allows us to compute a large number of estimates for the different $a_\mu (H_i)$ and for $a_\mu$(HLS), with their average values defining our reconstructed central values and their r.m.s. giving their standard deviations.

The fitted cross sections are also used to estimate the FSR contributions for the $\pi ^+ \pi ^-$, $\pi ^+ \pi ^-\pi ^0$ and $K^+ K^-$ final states and the Coulomb interaction effect, which is significant for the $K^+ K^-$ final state, as the kaons are slow in the $\phi$ energy region.

The HLS model functions describe VP amputated data; accordingly, all the data submitted to our global fits are amputated from their photon VP factor. Uncertainties related to VP amputation and FSR estimates are included below as separate systematics.

**Table 7:** $$\hbox {EBHLS}_2$$ contributions to $$10^{10} \times a_\mu ^\mathrm{HVP-LO}$$ integrated up to 1.05 GeV, including FSR and Coulomb interaction among the (slow) kaons involved in the $$K^+ K^- $$ final state. The running conditions are indicated at the top of the Table; BS and RS stand for the so-called basic and reference variants, respectively, defined in [19]. The last column displays the evaluation through a direct integration of the data

$\hbox {EBHLS}_2$	BS ($\lambda _3=0$)		($\lambda _3 \ne 0$)		Data direct integration
	Excl. $\tau$	Incl. $\tau$	BS	RS	
$\pi ^+ \pi ^-$	$493.12 \pm 0.98$	$492.77 \pm 0.85$	$492.77\pm 0.86$	$493.00\pm 0.90$	$496.26\pm 3.46$
$\pi ^0 \gamma$	$4.41 \pm 0.02$	$4.40 \pm 0.02$	$4.41 \pm 0.02$	$4.41 \pm 0.02$	$4.58 \pm 0.08$
$\eta \gamma$	$0.64 \pm 0.01$	$0.65 \pm 0.01$	$0.65 \pm 0.01$	$0.65 \pm 0.01$	$0.55 \pm 0.06$
$\pi ^+ \pi ^- \pi ^0$	$44.40\pm 0.32$	$44.41 \pm 0.32$	$44.45 \pm 0.32$	$44.41 \pm 0.30$	$44.80 \pm 1.72$
$$K^+ K^- $$	$$18.20 \pm 0.10$$	$$18.17 \pm 0.09$$	$$18.20\pm 0.09$$	$$18.29\pm 0.11$$	$$18.98 \pm 0.28$$
$K_L K_S$	$11.67 \pm 0.06$	$11.67 \pm 0.06$	$11.66 \pm 0.06$	$11.60 \pm 0.06$	$12.61 \pm 0.27$
HLS Sum	$572.44 \pm 1.08$	$572.06 \pm 0.95$	$572.14\pm 0.95$	$573.07 \pm 1.00$	$577.77 \pm 3.89$
$\chi ^2/N_{\mathrm{pts}}$	1179/1280	1285/1365	1269/1365	1262/1365	$\times$
Probability	93.3%	83.1 %	90.0 %	91.5 %	$\times$

Regarding the FSR correction of the $\pi ^+\pi ^-\pi ^0$ channel, we assume that the FSR correction of the 2$\pi$ channel applies to the 2$\pi$ subsystem of the 3$\pi$ final state as well. Thus we take $\sigma _{3\pi \gamma }(s)\approx \sigma _{3\pi }(s)[\frac{\alpha }{\pi }\,\eta (s')]$ as an estimate, assuming that the invariant mass square $s'$ of the charged $\pi ^+\pi ^-$ subsystem may be approximately identified as $s'\approx s$. This is justified because the main contribution comes from the $\rho ^0$ enhanced intermediate state $(\gamma \rho ^0 \pi ^0)$, *i.e.* the resonance enhancement happens at about the same $s\sim M_\rho ^2$ in both the $2\pi$ and the $3\pi$ channels (see also [93]). One then obtains a FSR contribution $0.17 \times 10^{-10}$ to which a 5% error is assigned. The same approximation is accepted by the BESIII Collaboration, and their recent 3$\pi$ spectrum [24] already includes the FSR correction computed this way.

Table 7 collects the results derived from $\hbox {EBHLS}_2$ fits performed under various conditions. The largest difference between the central values for the HLS sums does not exceed $0.4\times 10^{-10}$ and reflects the effect of using or not the $\tau$ dipion spectra – together with slightly improved uncertainties ($\simeq$ 10%) in the former option. The second data column collects the results derived by assuming $\lambda _3\equiv 0$ and $\delta P^\tau (s)$ third degree (i.e. the previous $\hbox {BHLS}_2$ framework); the third data column information is derived by letting $\lambda _3$ be free and fixing the $\delta P^\tau (s)$ degree to 2. Despite their different probabilities, their HVPs differ by only $6 \times 10^{-12}$ and their uncertainties as well. Comparing the third and fourth data columns also shows that the gain achieved by using the primordial mixing mechanism [19] is, by now, negligible.

Therefore, it looks consistent to choose as final evaluation of the BHLS channel contribution to $a^{\mathrm{HVP-LO}}_\mu$ up to 1.05 GeV:

$$\begin{aligned} a_\mu ^{\mathrm{HVP-LO}} (HLS) =[572.14 \pm 0.95] \times 10^{-10} \end{aligned}$$

up to additional systematics considered just below.

On the other hand, the last data column in Table 7 displays the results derived by a direct integration of the annihilation data; in this approach, the normalization of each of the combined spectra is the nominal one, and all uncertainties (correlated or not) are combined to provide its weight in the combined spectrum. This brings us back to the discussion presented in the previous subsection: It is not surprising to observe the data shifting compared to expectations and their uncertainties enlarged by the correlated contributions. This effect is the largest for the $\pi \pi$ contribution but represents only a $\simeq 1 \sigma _\mathrm{exp}$ effect. The difference for the dikaon contributions is rather due to taking the CMD3 data into account in the direct integration, whereas they are absent from the set of data samples submitted to the $\hbox {EBHLS}_2$ fit procedure (see [19]); their effect is, nevertheless, taken into account as systematics.

### Systematics in the HLS contribution of the muon HVP

Section 11.1 has illustrated, specifically on the $\pi \pi$ channel, that a possible hint for a significant bias induced by the global fit method itself is tiny. Indeed, Table 5 shows that, as long as the fit probabilities are good, the values for $a_\mu (\pi \pi )$ derived from the fit are very close to the KLOE Collaboration’s own evaluations [80]. More precisely, the $\hbox {EBHLS}_2$ fit estimates are distant by only $0.28 \sigma _\mathrm{exp.}$,$0.25 \sigma _\mathrm{exp.}$ and $0.15 \sigma _\mathrm{exp.}$ from the KLOE direct integration evaluations for the KLOE10 (78% prob.), KLOE12 (80% prob.) and the KLOE85 combined data sets (65% prob.), respectively. The example of KLOE08 is, however, also interesting: Indeed, even if the fit probability is poor (14%) – and for this reason excluded from our reference set of data samples – the fit differs from the KLOE direct integration of this spectrum by only $1.6 \sigma _\mathrm{exp.}$.

On the other hand, the set of accepted data samples being similar, Table 6 also indicates that the way the normalization uncertainty is dealt with accounts for the bulk of the differences between the various approaches. The (similar) choices made by KNT [91] and us [75] appear to be the best-grounded ones and lead to consistent central values. The better precision reached within the broken HLS frameworks mostly proceeds from the global fit tool they allow, which numerically correlates the various annihilation channels as if the statistics in each channel were larger than nominal. We should also note the marvelous agreement (still valid) between the $\hbox {BHLS}_2$ prediction and the Lattice QCD form factor spectra [56] emphasized in [19] (see Fig. 8 therein) and in Fig. 1 above.

Therefore, once a canonical treatment of the various kinds of systematic uncertainties reported by the various groups, together with their spectra, is applied, one may consider that these are already absorbed in the uncertainties derived from the minuit minimization procedure.

However, additional sources of uncertainty can be invoked. Until $\hbox {EBHLS}_2$ is experimentally strengthened by new high statistics dipion spectra to be collected in the $\tau$ decay, one may consider that the difference between using or not using $\tau$ data contributes a systematic uncertainty which can increase $a_\mu ^{\mathrm{HVP-LO}}$ by at most $0.32 \times 10^{-10}$ (see Table 7). On the other hand, it is worthwhile to anticipate the treatment of the $[\pi ^0,\eta ,{\eta ^\prime }]$ mixing properties addressed in this paper from Sect. 12 onwards. This will emphasize the relevance of the kinetic breaking mechanism defined in Sect. 4 and lead us to consider a possible shift of $a_\mu ^{\mathrm{HVP-LO}}$ by $\pm 0.3 \times 10^{-10}$ (see Sect. 19).

The poor knowledge of the dipion spectrum in the $\phi$ mass region has been emphasized. Here also, considering the numbers given in Sect. 11.1, the central value for $a_\mu ^\mathrm{HVP-LO}$ might undergo a shift of $+0.28 \times 10^{-10}$. In [19], assuming their systematics are uncorrelated, fits related to the CMD3 dikaon data [71, 72] are reasonably good; therefore, leaving them outside our reference sample set may result in missing $+0.54 \times 10^{-10}$ when evaluating $a_\mu (K{\overline{K}})$.

The still preliminary SND dipion data [25] examined above have been submitted to our standard global fit by inclusion in the set of accepted dipion spectra. The fit returns, with a probability of 66.2%,

$$\begin{aligned} a_\mu ({\pi \pi }) = 493.26 \pm 0.81 \quad \mathrm{and}~~~ a_\mu ^{\mathrm{HVP-LO}}=572.60\pm 0.89, \end{aligned}$$

in units of $10^{-10}$. As the average $\chi ^2/N_\mathrm{points}$ for this SND sample is large ($\simeq 2$), we gave up including it inside the fitted sample set and preferred affecting the difference $+0.48 \times 10^{-10}$ to the systematics.

Other well-identified sources of systematics deserve to be addressed: (i) The uncertainty^44^ on the total photon VP ($\gamma V P$) has been estimated to $\pm 0.29 \times 10^{-10}$, and (ii) the FSR effect in the HLS energy range covers its contributions to the $\pi ^+\pi ^-$, $\pi ^+\pi ^-\pi ^0$ and $K^+K^-$ annihilation channels; its value amounts to $4.81 \times 10^{-10}$ over the whole non-perturbative region and may be conservatively attributed a 2% uncertainty.^45^ In the non-HLS range above 1.05 GeV, the contributions listed in Table 8 include FSR effects estimated via the quark parton model. The $n_f=$ 3-, 4- and 5-flavor range LO contributions are to be multiplied by the radiative correction factors $\frac{3\alpha }{4\pi }\,N_c\,\sum _{i=1}^{n_f} Q^2_i$, which yields $0.42 \times 10^{-10}$ as a total FSR effect, and one may assign a 10% uncertainty here. The uncertainty values just given actually affect the HVP over the whole energy range.

These possible additional sources of systematics rather play as shifts and, thus, should not be combined with the uncertainty returned by the fit. Summing up all these estimates, our final result can be completed with the most pessimistic systematic uncertainty^46^:

56 $$\begin{aligned} a_\mu ^{\mathrm{HVP-LO}} (\mathrm{HLS}, \sqrt{s} \le 1.05~\mathrm{GeV})= & {} 572.14 \pm [0.95]_\mathrm{fit} \nonumber \\&+ [^{+2.31}_{-0.69}]_\mathrm{syst.} \end{aligned}$$

in units of $10^{-10}$.

Finally, the tiny contribution generated by the “non-HLS” channels^47^ should be considered to fully complement the $[s_0=m_{\pi ^0}^2,s_\mathrm{cut}]$ energy interval contribution to the muon HVP; it has been re-estimated by direct integration of the (sparse) existing data to $=[1.21 \pm 0.17] \times 10^{-10}$.

### The muon HVP and anomalous magnetic moment

To finalize our HLS-based estimate of the muon HVP, our resulting Eq. (56), complemented for the non-HLS channel contribution below $\sqrt{s_\mathrm{cut}}=1.05$ GeV already given, should be supplied by the contributions from above this energy limit. This is displayed in the left-hand part of Table 8; the different contributions up to $\sqrt{s_\mathrm{cut}}=5.20$ GeV are derived by a numerical integration of the experimental data (annihilation spectra and *R*(*s*) ratio measurements) as for the $\Upsilon$ energy interval. This part carries a significant uncertainty. The rest, evaluated using perturbative QCD, is reported under the tag “pQCD” and exhibits high precision.

**Table 8:** The left-hand side displays the updated contributions to $a_\mu ^{\mathrm{HVP-LO}}$ from the various energy regions and includes the contribution of the non-HLS channels in the $\sqrt{s}\! <\! 1.05$ GeV region; only total errors are shown. The right-hand side provides the various contributions to $a_\mu$ in accord with Table 1 in [2] together with our own datum for $a_\mu ^{HVP-LO}$. The result for $\Delta a_\mu \!=\!a_\mu (\mathrm{exp})\!-\!a_\mu (\mathrm{th})$, based on the $\hbox {EBHLS}_2$ fit and the average of the BNL and FNAL measurement [1, 4], is also given; the effect of the systematic is discussed in the body of the text

Contribution from	Energy range	$10^{10} \times a_\mu ^{\mathrm{HVP-LO}}$	Contribution from	$10^{10} \times a_\mu$
Missing channels	$\sqrt{s} \le 1.05$	$1.21 \pm 0.17$	LO-HVP	$687.48 \pm 2.93 +\left[ ^{+2.31}_{-0.69} \right] _{\mathrm{syst}}$
$J/\psi$		$8.94\pm 0.59$	NLO HVP [91]	$-9.83 \pm 0.07$
$\Upsilon$		$0.11 \pm 0.01$	NNLO HVP [94]	$~1.24 \pm 0.01$
Hadronic	(1.05, 2.00)	$62.95 \pm 2.53$	LBL [2, 95]	$9.2 \pm 1.9$
Hadronic	(2.00, 3.20)	$21.63 \pm 0.93$	NLO-LBL [96]	$0.3 \pm 0.2$
Hadronic	(3.20, 3.60)	$3.81 \pm 0.07$	QED [97, 98]	$11 658 471.8931 \pm 0.0104$
Hadronic	(3.60, 5.20)	$7.59 \pm 0.07$	EW [99, 100]	$15.36\pm 0.11$
pQCD	(5.20, 9.46)	$6.27 \pm 0.01$	Total theor.	$11~659~175.33\pm 3.49 +\left[ ^{+1.62}_{-0.0} \right] _{\mathrm{syst}}$
Hadronic	(9.46, 11.50)	$0.87 \pm 0.05$	Exper. aver. [4]	$11 659 206.1 \pm 4.1$
pQCD	(11.50,$\infty$)	$1.96 \pm 0.00$	$10^{10} \times \Delta a_\mu$	$30.77 \pm 5.38 -\left[ ^{+2.31}_{-0.69} \right] _{\mathrm{syst}}$
Total	1.05 $\rightarrow \infty$ + missing chann.	$115.34 \pm 2.77$	Significance ($n \sigma$)	$5.72 \sigma$

Summing up the various components, our evaluation of the muon HVP integrated over the full energy range is:

57 $$\begin{aligned} a_\mu ^{\mathrm{HVP-LO}} = \left\{ 687.48 \pm [2.93]_\mathrm{fit} + [^{+2.31}_{-0.69}]_\mathrm{syst.} \right\} \times 10^{-10}. \end{aligned}$$

In order to derive the anomalous magnetic moment of the muon, its HVP should be complemented with the contributions other than the LO–VP: higher-order HVP effects, light-by-light, QED and electroweak inputs. For consistency with others, we have used for these the values given in Table 1 of [2]. This sums up to:

58 $$\begin{aligned} a_\mu ^{HLS} =11,659,175.33\pm 3.49 +\left[ ^{+2.31}_{-0.69} \right] _{\mathrm{syst}} \end{aligned}$$

in units of $10^{-10}$, which exhibits a $5.72 \sigma$ difference with the experimental average [4]. If taking into account the possible shift of the $a_\mu$ central value following from our systematics upper bound, the significance for $\Delta a_\mu =a_\mu ^{BNL}-a_\mu ^{HLS}$ can decrease to $5.31 \sigma$.

A final remark should be asserted: one may find amazing the jump in significance of $\Delta a_\mu$ compared to [19]; a mere comparison of the EBHLS2 numerical outcome with those of our previous work clearly shows that it is almost unchanged. The changes reported here are solely due to the 30% reduction of the uncertainty produced by averaging^48^ the FNAL [4] and BNL [1]measurements.

### A challenging value for $a_\mu ^{HVP-LO}$

In Sect. 9.2 , we have revisited the consistency topic of the various available dipion spectra. The most relevant fit properties of these are collected in Table 3. Comparing the $\chi ^2/N$ averages for NSK, KLOE and BaBar in global fits where each of them is used as single representative for the $\pi \pi$ channel and fits using their pairwise combinations permits several conclusions reflected by the fit probabilities displayed therein.^49^ Namely:

- The tension exhibited by the pairwise fit involving KLOE and NSK is marginal compared to the fits using each of them in isolation: the fit probabilities are quite similar.
- In the pairwise fit involving KLOE and BaBar, one observes a strong tension reflected by the drop in probability between the pairwise fit and those with KLOE and BaBar in isolation.
- The pairwise fit of the NSK and BaBar spectra also exhibits some tension between them, but at a softer level: if the drop in probability versus the NSK fit in isolation is large (a factor of $\simeq 2$), the corresponding drop in probability versus BaBar in isolation is small ($62.9 \% \rightarrow 51.7 \%$).

This motivates us to examine a global fit involving the NSK,^50^ BESIII [26, 27], Cleo-c [48] and BaBar spectra, the KLOE data samples being excluded. In contrast to the fits reported in Table 3, this special fit includes the three-pion spectra and involves 1500 data points; it converges at $\chi ^2_\mathrm{total}=1484$, yielding a 39.5 % probability. This fit is not as good as the standard one (see the subsection just above), which results in a $\simeq 90\%$ probability, but is reported in some detail here for completeness.

**Fig. 9:**
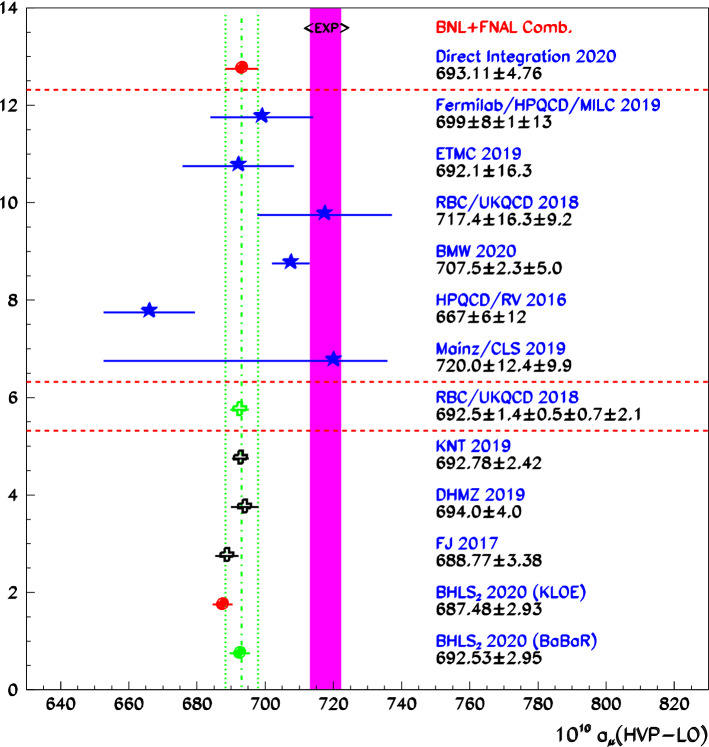
Recent evaluations of $10^{10} \times a_\mu ^{\mathrm{HVP-LO}}$: on top, the result derived by a direct integration of the data combined with perturbative QCD; the dotted vertical lines indicate the $\pm 1 \sigma$ interval. The LQCD data are followed by the results derived using dispersive methods from [90, 91, 101]. The two HVP-LO evaluations derived using $\hbox {EBHLS}_2$ fitting codes are given at the bottom (see text). The $\pm 1 \sigma$ interval corresponding to the BNL+FNAL average [4] is shown by the shaded area

For the region up to 1.05 GeV, one gets

59 $$\begin{aligned} a_\mu (\pi \pi )= & {} 497.83 \pm 0.90\quad \mathrm{and}\nonumber \\ a_\mu (\mathrm{HLS})= & {} 577.19\pm 1.00 \end{aligned}$$

in units of $10^{-10}$. The corresponding standard results can be found in Table 7, more precisely its third data column; the increases produced by BaBar (excluding KLOE) for these quantities are equal: $\delta a_\mu (\pi \pi )=\delta a_\mu (HLS)=5.07 \times 10^{-10}$. So the difference is fully carried by the $\pi \pi$ channel. The information displayed in the left-hand Table 8 allows us to derive the full HVP-LO:

60 $$\begin{aligned} a_\mu ^\mathrm{HVP-LO}(\mathrm{BaBar})= [692.53 \pm 2.95] \times 10^{-10} \end{aligned}$$

and can be affected by the same additional systematic uncertainty proposed above. Finally, the difference between $a_\mu ^\mathrm{HVP-LO}(\mathrm{BaBar})$ and the experimental average [4] drops to $\Delta a_\mu =a_\mu ^\mathrm{avrg}-a_\mu ^\mathrm{HVP-LO}(\mathrm{BaBar})= 23.65 \pm 5.38$ and exhibits a statistical significance of $4.78 \sigma$ not counting the systematic uncertainty effect. Taking it into account, the significance may drop to $4.35 \sigma$.

### The different muon HVP-LO evaluations

Figure 9 collects recent evaluations of the leading order muon HVP. A numerical integration of the annihilation and *R*(*s*) data, appropriately completed by perturbative QCD calculations (see Table 8), yields the entry displayed at the top of the figure. It is followed by some reference evaluations derived by LQCD collaborations, namely [3, 102–106]; the second of the RBC/UKQCD evaluations [104] relies on mixing LQCD and dispersive information. This datum is just followed by the HVP-LO dispersive evaluations from [90, 91, 101]. The bottom pair of data points are the $\hbox {EBHLS}_2$-based evaluations of the full HVP-LO derived in this study. It can be noted that the preferred $\hbox {EBHLS}_2$ evaluation (90% prob.), tagged by KLOE, is $5.30 \times 10^{-10}$ smaller than KNT19 [91] and $6.54 \times 10^{-10}$ smaller than DHMZ19 [90]. In contrast, the challenging evaluation (40% prob.), tagged by BaBar, differs only by $0.25 \times 10^{-10}$ and $1.47 \times 10^{-10}$ from KNT19 and DHMZ19, respectively.

The difference in our two evaluations ($5.05 \times 10^{-10}$) compared to their respective accuracies ($2.9 \times 10^{-10}$), makes us reluctant to propose a mixture of these or a common KLOE+BaBar fit evaluation. Nevertheless, it shows that model dependence is not the main source of disagreement between the various dispersive evaluations.

The difference between the most recent BMW evaluation [3] and those based on the various dispersion relation (DR) approaches ranges between $13 \times 10^{-10}$ and $20 \times 10^{-10}$; it appears difficult to fill such a gap with only $e^+ e^-$ annihilation data below $\simeq 1$ GeV, an experimentally well-explored energy region.

## The $\hbox {EBHLS}_2$ approach to the $[\pi ^0,\eta ,{\eta ^\prime }]$ system

The mixing properties of the $[\pi ^0,\eta ,{\eta ^\prime }]$ system underlie the physics of the light meson radiative decays as well as the amplitudes for the $e^+e^- \rightarrow P \gamma$ annihilations which are obviously tightly related. The other important data involved in this issue, namely the $P \gamma \gamma$ decays and the $V\gamma {\eta ^\prime }$ couplings, are also part of the $\hbox {EBHLS}_2$ scope.^51^

Phenomenological descriptions of the $[\pi ^0,\eta ,{\eta ^\prime }]$ system were first based on using *U*(3) symmetric $VP\gamma$ coupling expressions enriched by parameterizations of nonet symmetry breaking in both the pseudoscalar and vector sectors as done in [107], for instance. A first HLS-based model including its anomalous sectors [12] provided a unified framework which encompasses the $P \rightarrow \gamma \gamma$ decays and the radiative decays of the form $VP\gamma$ [108]. The effective Lagrangian approach, started long ago (see [60], for instance), has been pursued up to very recently (a comprehensive list of previous references can be found in [109]).

On the other hand, chiral perturbation theory and its extension (EChPT), originally formulated by Kaiser and Leutwyler [33, 34], allowed us to fully address the $[\pi ^0,\eta ,{\eta ^\prime }]$ mixing and gave rise to the singlet-octet basis description in terms of two angles and two decay constants. It was shown in [31] that this approach is naturally accommodated within a HLS framework with only one mixing angle provided that SU(3) and nonet symmetry breakings are also accounted for within its effective Lagrangian. Besides the singlet-octet basis formulation, another convenient formulation, known as quark flavor basis, has been proposed in [35, 36]; its properties and its relation with the singlet-octet formulation have been thoroughly reported in [37].

Finally, isospin symmetry breaking in the $[\pi ^0,\eta ,{\eta ^\prime }]$ system has also been considered and parameterized as $\eta$ and ${\eta ^\prime }$ admixtures inside the physically observed $\pi ^0$ meson [37, 39]. Additional isospin breaking effects have also been studied, generated by having different $u{\overline{u}}$ and $d{\overline{d}}$ decay constants [38].

It happens that the parameters which substantiate the singlet-octet [33, 34] and quark flavor basis parameterizations [35–37] can be accessed within the $\hbox {EBHLS}_2$ framework as reported above – and in [19]. Its Lagrangian leads to quite similar expressions and also includes isospin breaking contributions. Moreover, the global fits performed and already referred to in the previous sections allow for precise numerical determinations of the mixing parameters of the $[\pi ^0,\eta ,{\eta ^\prime }]$ system in both the singlet-octet and quark flavor bases.

In the following, we report on works performed in parallel with both the $\hbox {BHLS}_2$ framework previously defined [19] and its $\hbox {EBHLS}_2$ extension analyzed in the preceding sections. However, the detailed analysis of the $[\pi ^0,\eta ,{\eta ^\prime }]$ system exhibits properties which make relevant a further analysis related to the kinetic breaking introduced above to account for the $\tau$ dipion spectra, especially the Belle one, by far the most precise spectrum.

If the fate of $\hbox {EBHLS}_2$ versus $\hbox {BHLS}_2$ is tightly related to a forthcoming high statistics measurement of the dipion spectrum in the $\tau$ decay, the analysis of the $[\pi ^0,\eta ,{\eta ^\prime }]$ system nevertheless reveals constraints among the three terms of the kinetic breaking matrix $X_H$ which should be addressed and which, certainly, influence this picture.

## The axial currents from the $\hbox {EBHLS}_2$ effective Lagrangian

The main tool to address the $[\pi ^0,\eta ,{\eta ^\prime }]$ mixing topic is the axial currents which are derived from the pseudoscalar kinetic energy term Eq. (23) – restated here for convenience:

61 $$\begin{aligned} \displaystyle K = \mathrm{Tr} \left[ \partial P_\mathrm{bare} X_A \partial P_\mathrm{bare} X_A\right] + 2 ~ \{ \mathrm{Tr} \left[ X_H \partial P_\mathrm{bare} \right] \}^2 \end{aligned}$$

by the derivatives:

62 $$\begin{aligned} \begin{array}{ll} \displaystyle J_\mu ^a = f_\pi \frac{\partial K}{\partial (\partial _\mu P^a_\mathrm{bare})},&~ \left[ P_\mathrm{bare} =\sum _{a=0, \cdots 8} T_a P^a_\mathrm{bare} \right] , \end{array} \end{aligned}$$

with respect to the entries associated with each of the *U*(3) basis matrices normalized such that Tr$[T_aT_b]=\delta _{ab}/2$; the breaking matrix $X_A$ is given in Eq. (10) and $X_H$ can be found in Eq. (20). They are given by (summation over repeated indices is understood):

63 $$\begin{aligned} \displaystyle J_\mu ^a= & {} 2f_\pi \mathrm{Tr} \left[ T_a X_A T_b X_A\right] \partial P_{bare}^b + 4 ~ \mathrm{Tr} \left[ X_H \partial P_{bare} \right] \nonumber \\&\times \mathrm{Tr} \left[ X_H T_a \right] ( \delta _{a,0} + \delta _{a,3} + \delta _{a,8}) . \end{aligned}$$

The axial currents relevant for our purpose are:

64 $$\begin{aligned} \left\{ \begin{array}{lll} &{}J^{3}/f_\pi =\displaystyle \partial \pi ^3_b +\frac{\Delta _A }{\sqrt{3}} \left[ \sqrt{2} \partial \eta ^0_b+\partial \eta ^8_b\right] \\ &{}\qquad \qquad + \lambda _3 \left[ \lambda _3 \partial \pi ^3_b + \lambda _0 \partial \eta ^0_b+ \lambda _8 \partial \eta ^8_b \right] ,\\ &{}J^{0}/f_\pi =\displaystyle D \partial \eta ^0_b + G \partial \eta ^8_b + \sqrt{\frac{2}{3}} \Delta _A ~\partial \pi ^3_b \\ &{}\qquad \qquad + \lambda _0 \left[ \lambda _3 \partial \pi ^3_b + \lambda _0 \partial \eta ^0_b+ \lambda _8 \partial \eta ^8_b \right] ,\\ &{}J^{8}/f_\pi =\displaystyle G \partial \eta ^0_b + F \partial \eta ^8_b + \frac{\Delta _A}{\sqrt{3}} ~ \partial \pi ^3_b\\ &{}\qquad \qquad + \lambda _8 \left[ \lambda _3 \partial \pi ^3_b + \lambda _0 \partial \eta ^0_b+ \lambda _8 \partial \eta ^8_b \right] , \end{array} \right. \end{aligned}$$

where the subscript *b* stands for *bare * and:

65 $$\begin{aligned} \begin{array}{lll} \displaystyle D=\frac{z_A^2+2}{3} ,&\displaystyle F=\frac{2z_A^2+1}{3} ,&\displaystyle G=\frac{\sqrt{2}}{3} (1-z_A^2) , \end{array} \end{aligned}$$

in terms of $z_A$, the $(s,{\overline{s}})$ entry of the $X_A$ breaking matrix. Equation (31) and the definitions given in Sect. 4 allow us to express the axial currents at first order in breakings in terms of the renormalized *R*-fields – those which render canonical the PS kinetic energy term – by:

66 $$\begin{aligned} \left\{ \begin{array}{ll} \displaystyle J^3_\mu /f_\pi {=}&{} \left( 1+\frac{\lambda _3^2 }{2}\right) \partial _\mu \pi ^3_R + \left( \frac{\lambda _3 {\widetilde{\lambda }}_0}{2} + \frac{\Delta _A}{\sqrt{6}} \right) \partial _\mu \eta _R^0 \\ &{}\quad + \left( \frac{\lambda _3 {\widetilde{\lambda }}_8}{2} + \frac{\Delta _A}{2\sqrt{3}} \right) \partial _\mu \eta _R^8 , \\ \displaystyle J^0_\mu /f_\pi =&{} \left( \frac{\Delta _A}{\sqrt{6}}+ \frac{\lambda _0 \lambda _3}{2} \right) \partial _\mu \pi ^3_R + \left( z_A C +\frac{\lambda _0 {\widetilde{\lambda }}_0}{2} \right) \partial _\mu \eta _R^0 \\ &{}\quad + \left( - z_A A + \frac{\lambda _0 {\widetilde{\lambda }}_8}{2} \right) \partial _\mu \eta _R^8 ,\\ \displaystyle J^8_\mu /f_\pi =&{} \left( \frac{\Delta _A}{2\sqrt{3}}+ \frac{\lambda _3 \lambda _8}{2} \right) \partial _\mu \pi ^3_R + \left( - z_A A + \frac{\lambda _8 {\widetilde{\lambda }}_0}{2} \right) \partial _\mu \eta _R^0 \\ &{}\quad + \left( z_A B + \frac{\lambda _8{\widetilde{\lambda }}_8}{2} \right) \partial _\mu \eta _R^8 , \end{array} \right. \end{aligned}$$

${\widetilde{\lambda }}_0$ and ${\widetilde{\lambda }}_8$ having been given in Eqs. (25) in terms of *A*, *B*, *C*, $\lambda _0$ and $\lambda _8$. The following matrix elements are of purpose for the $\eta -{\eta ^\prime }$ mixing topic ($\partial _\mu \rightarrow iq_\mu$, outgoing momentum):

67 $$\begin{aligned} \left\{ \begin{array}{ll} \displaystyle<0|J^0_\mu |\eta _R^0>=&{} f_\pi \left\{ z_A C +\frac{\lambda _0 {\widetilde{\lambda }}_0}{2} \right\} iq_\mu (\eta _R^0) \\ &{} \equiv i f_0 ~ q_\mu (\eta _R^0) ,\\ \displaystyle<0|J^0_\mu |\eta _R^8> =&{} f_\pi \left\{ - z_A A + \frac{\lambda _0 {\widetilde{\lambda }}_8 }{2} \right\} iq_\mu (\eta _R^8) \\ &{}\equiv i b_0 ~q_\mu (\eta _R^8) ,\\ \displaystyle<0|J^8_\mu |\eta _R^8> =&{} f_\pi \left\{ z_A B + \frac{\lambda _8{\widetilde{\lambda }}_8}{2} \right\} iq_\mu (\eta _R^8) \\ &{} \equiv i f_8 ~ q_\mu (\eta _R^8) ,\\ \displaystyle <0|J^8_\mu |\eta _R^0> =&{} f_\pi \left\{ - z_A A + \frac{\lambda _8 {\widetilde{\lambda }}_0}{2} \right\} iq_\mu (\eta _R^0) \\ &{} \equiv i b_8 ~ q_\mu (\eta _R^0) , \end{array} \right. \end{aligned}$$

which define the decay constants $f_0$, $f_8$, $b_0$ and $b_8$. One observes that the kinetic breaking affects all these matrix elements; in order to connect with [31], one should identify the usual kinetic (’t Hooft) breaking term with $\lambda _0^2$.

Regarding the other fields, $\hbox {EBHLS}_2$ does not go beyond the BKY breaking [16, 17] as their *R* renormalized and physical states coincide. They relate to their bare partners by [14]:

68 $$\begin{aligned} \displaystyle \pi ^\pm _b= & {} \displaystyle \pi ^\pm _R,\quad \displaystyle K^{\pm }_b= \frac{1}{\sqrt{z_A}} \left[ 1-\frac{\Delta _A}{4}\right] K^{\pm }_R, \nonumber \\ \displaystyle K^{0}_b= & {} \displaystyle \frac{1}{\sqrt{z_A}} \left[ 1+\frac{\Delta _A}{4}\right] K^{0}_R . \end{aligned}$$

This transformation to *R* fields brings the corresponding part of the kinetic energy term into canonical form [14]. The corresponding axial currents are written as:

69 $$\begin{aligned} \displaystyle J^{\pi ^\pm }_\mu= & {} f_\pi \partial _\mu \pi ^\mp _R, \displaystyle J^{K^\pm }_\mu =\sqrt{z_A} \left[ 1+\frac{\Delta _A}{4}\right] f_\pi \partial _\mu K^\mp _R , \nonumber \\ \displaystyle J^{K^0}_\mu= & {} \sqrt{z_A} \left[ 1-\frac{\Delta _A}{4}\right] f_\pi \partial _\mu {\overline{K}}^0_R . \end{aligned}$$

Using also the expression for $J^{3}_\mu$ given just above, one can use the expectation values:

70 $$\begin{aligned} \displaystyle <0| J^P_\mu |P(q)>=i f_{P}~q_\mu ,\quad (P=~\pi ^\pm ,\pi ^0,K^\pm ,K_L/K_S) \end{aligned}$$

to derive:

71 $$\begin{aligned} \displaystyle f_{\pi ^0}= & {} \left[ 1+\frac{\lambda _3^2}{2} \right] f_\pi , \displaystyle f_{K^\pm } \equiv f_K =\sqrt{z_A} \left[ 1+\frac{\Delta _A}{4} \right] f_\pi , \nonumber \\ \displaystyle f_{K^0}= & {} \sqrt{z_A} \left[ 1-\frac{\Delta _A}{4}\right] f_\pi \end{aligned}$$

and then one gets $z_A=[f_K/f_\pi ]^2$ up to isospin breaking corrections. Moreover, it should also be noted that, once $\lambda _3$ is floating, $f_{\pi ^0}$ may differ from $f_\pi (\equiv f_{\pi ^+})$ by as much as $\simeq$ 2.5 %, in line with the remarks in [110]. This comes out of our fits which successfully involve, besides the $\tau$ dipion spectra, the $e^+ e^- \rightarrow (\pi ^0/\eta ) \gamma$ annihilation data and the widths of the anomalous decays $\{P \rightarrow \gamma \gamma , P\in (\pi ^0,\eta ,{\eta ^\prime })\}$.

## The $\eta -{\eta ^\prime }$ mixing: the octet-singlet basis parameterization

The axial current matrix elements in the two-angle scheme are written as:

72 $$\begin{aligned} <0|J^{0,8}_\mu |\eta /\eta ^\prime >= i F^{0,8}_{\eta /\eta ^\prime } q_\mu \end{aligned}$$

in terms of the physical $$\eta /\eta ^\prime $$ fields carrying a momentum $$q_\mu $$. As usual, one defines the $$ F^{0,8}$$ couplings and the $$\theta _{0,8}$$ mixing angles by [32–34]:

73 $$\begin{aligned} \left\{ \begin{array}{lll} \displaystyle F^{8}_{\eta }&{} =F^8 \cos {\theta _8} =&{} \displaystyle f_8 \cos {\theta _P} -b_8 \sin {\theta _P} ,\\ \displaystyle F^{8}_{\eta ^\prime }&{} =F^8 \sin {\theta _8} =&{} \displaystyle f_8 \sin {\theta _P} + b_8 \cos {\theta _P} ,\\ \displaystyle F^{0}_{\eta }&{} =- F^0 \sin {\theta _0} =&{} \displaystyle b_0 \cos {\theta _P} -f_0 \sin {\theta _P} ,\\ \displaystyle F^{0}_{\eta ^\prime }&{} =F^0 \cos {\theta _0} =&{} \displaystyle b_0 \sin {\theta _P} + f_0 \cos {\theta _P} , \end{array} \right. \end{aligned}$$

the *f*’s and *b*’s being those given by Eq. (67). The $\pi ^3_R$ components of the physical $\eta$ and ${\eta ^\prime }$ fields, providing contributions of order $\mathcal{O}(\delta ^2)$ in the matrix elements $<0|J^{0,8}_\mu |\eta /\eta ^\prime>$, are discarded. Using Eqs. (67) and (73) and the definitions for *A*, *B* and *C* given in Sect. 4.1, one derives:

74 $$\begin{aligned} \left\{ \begin{array}{ll} \displaystyle [F^0]^2= [F^{0}_{\eta }]^2 + [F^{0}_{\eta ^\prime }]^2 = f_0^2+b_0^2=\left[ \frac{z_A^2+2}{3}+ \lambda _0^2 \right] f_\pi ^2,\\ \displaystyle [F^8]^2= [F^{8}_{\eta }]^2 + [F^{8}_{\eta ^\prime }]^2 =f_8^2+b_8^2= \left[ \frac{2 z_A^2+1}{3}+ \lambda _8^2 \right] f_\pi ^2 . \end{array} \right. \end{aligned}$$

The no-BKY breaking limit is obtained by letting $z_A \simeq [f_K/f_\pi ]^2 \rightarrow 1$. The correspondence with others [33, 36, 37] becomes manifest, when using the following identities:

75 $$\begin{aligned} \left\{ \begin{array}{ll} \displaystyle \frac{z_A^2+ 2}{3}= \frac{2 z_A+1}{3}+ \frac{(z_A-1)^2}{3} \\ \displaystyle \frac{2 z_A^2+ 1}{3}= \frac{4 z_A-1}{3}+ \frac{2}{3}(z_A-1)^2 \end{array} \right. \end{aligned}$$

which provide:

76 $$\begin{aligned} \left\{ \begin{array}{ll} \displaystyle [F^0]^2= \left\{ \frac{2 z_A+1}{3}+ \left[ \lambda _0^2 +\frac{(z_A-1)^2}{3} \right] \right\} f_\pi ^2, \\ \displaystyle [F^8]^2=\left\{ \frac{4 z_A -1}{3}+ \left[ \lambda _8^2 +\frac{2}{3}(z_A-1)^2 \right] \right\} f_\pi ^2. \end{array} \right. \end{aligned}$$

If one assumes $$\lambda _8=0$$, these expressions coincide at leading order in breakings with their usual EChPT analogs [33, 37]; one should note the $$(z_A-1)^2$$-dependent terms, which, even if non-leading, are not negligible compared to the contributions provided by the kinetic (’t Hooft) breaking. Regarding the non-leading terms in the $$ [F^{0/8}]^2$$, we can anticipate our fit result analysis by mentioning that $$\lambda _0^2 \simeq 8 \times 10^{-2}$$ while the flavor breaking correction is governed by $$(z_A-1)^2 \simeq 9 \%$$.

At leading order in breakings, one also finds:

77 $$\begin{aligned} \displaystyle F_0 F_8 \sin {\left( \theta _0-\theta _8\right) }= \left[ \frac{\sqrt{2}}{3} (z_A^2-1) -\lambda _0\lambda _8 \right] f_\pi ^2, \end{aligned}$$

which vanishes when no breaking, leading to $\theta _8=\theta _0=\theta _P$. This expression can be rewritten:

78 $$\begin{aligned} \displaystyle F_0F_8 \sin {\left( \theta _0-\theta _8\right) }= & {} \frac{2\sqrt{2}}{3} (z_A-1) f_\pi ^2 \nonumber \\&+ \left[ \frac{\sqrt{2}}{3}(z_A-1)^2 -\lambda _0\lambda _8 \right] f_\pi ^2, \end{aligned}$$

which also coincides at leading in breaking with its EChPT analog [33, 37], as soon as $\lambda _8$ – which is not involved within EChPT – is dropped out.

The usual axial current matrix elements in the two-angle mixing scheme yield the following expressions in terms of the singlet-octet mixing angle $\theta _P$ and the BKY and kinetic breaking parameters:

79 $$\begin{aligned} \left\{ \begin{array}{ll} \displaystyle \tan {\theta _8}= \tan {\left[ \theta _P+\Psi _8\right] }, &{} \displaystyle \tan {\Psi _8}=\frac{b_8}{f_8}= - \frac{2 z_A A - \lambda _8 {\widetilde{\lambda }}_0}{2 z_A B + \lambda _8 {\widetilde{\lambda }}_8} ,\\ \displaystyle \tan {\theta _0}= \tan {\left[ \theta _P-\Psi _0 \right] }, &{} \displaystyle \tan {\Psi _0}=\frac{b_0}{f_0}= - \frac{2 z_A A - \lambda _0 {\widetilde{\lambda }}_8}{2 z_A C + \lambda _0 {\widetilde{\lambda }}_0} . \end{array} \right. \end{aligned}$$

Compared with [31], the expression for $\tan {\theta _8}$ is recovered in the limit $\lambda _8 \rightarrow 0$. Regarding $\tan {\theta _0}$, in the same limiting case, the leading order terms yield:

$$\begin{aligned} \tan {\Psi _0} = \frac{A}{C} \left[ 1 - \lambda _0^2 \frac{3(z_A+1)}{2 z_A (z_A+2)} \right] =\frac{A}{C} [1 - 0.80\lambda _0^2], \end{aligned}$$

which exhibits a behavior similar to the nonet symmetry breaking coefficient *x* defined in [31].

Equation (79) allows us to derive interesting expressions for the Kaiser–Leutwyler angles $\theta _0$ and $\theta _8$ in terms of the BHLS model parameters. Discarding terms of orders $\mathcal{O}[(z_A-1)^3]$ and $\mathcal{O}[(z_A-1)^2 \delta ]$ or higher^52^, one gets:

80 $$\begin{aligned} \left\{ \begin{array}{ll} \displaystyle \theta _8 +\theta _0 =&{} 2 \theta _P+ \frac{\sqrt{2}}{3} (z_A-1) \left[ \frac{(z_A-1)}{3} - \lambda _0^2+ \lambda _8^2 \right] , \\ \displaystyle \theta _8 -\theta _0 =&{} -\frac{2\sqrt{2}}{3} (z_A-1)+ \frac{\lambda _0\lambda _8}{z_A} + \frac{\sqrt{2}}{3} (z_A-1) \\ &{}\left[ (z_A-1) + \lambda _0^2+ \lambda _8^2 \right] . \end{array} \right. \end{aligned}$$

The expressions one can derive for $\theta _0$ and $\theta _8$ coincide with those in Eq. (84) in [37] at leading order. Let us anticipate the numerical information provided by our fits to indicate that $z_A-1 \simeq 0.3$, while the different squared $\lambda$ combinations stand at the few percent level at most; therefore, the breaking corrections affect both the sum and the difference in a significant way.

Equation (80) can be written:

81 $$\begin{aligned} \left\{ \begin{array}{ll} \displaystyle \theta _8 = \theta _P -\frac{\sqrt{2}}{3} (z_A-1) [1 -\lambda _8^2] + \frac{\lambda _0\lambda _8}{2 z_A} + \frac{2\sqrt{2}}{9} (z_A-1)^2 , \\ \displaystyle \theta _0 =\theta _P +\frac{\sqrt{2}}{3} (z_A-1) [1 -\lambda _0^2] -\frac{\lambda _0\lambda _8}{2 z_A} -\frac{\sqrt{2}}{9} (z_A-1)^2 . \end{array} \right. \end{aligned}$$

One may note the symmetry between the expressions, symmetry only spoiled by the term of order $\mathcal{O}[(z_A-1)^2]$. This shows that the departure from the one mixing angle scheme only reflects the breaking of the *SU*(3) and nonet (or kinetic) symmetries.

## The $\eta -{\eta ^\prime }$ mixing: the quark flavor basis parameterization

Besides the octet-singlet parameterization of the $\eta -{\eta ^\prime }$ system developed by [32–34] and referred to just above, another parameterization has been advocated by [35–37]; this challenging parameterization will be referred to as either quark flavor basis or FKS scheme. It seems to be worth analyzing how it shows up within the broken HLS framework. The axial currents relevant to determining how the FKS parameterization arises within $\hbox {EBHLS}_2$ are

82 $$\begin{aligned} \left\{ \begin{array}{ll} \displaystyle J_\mu ^q= \sqrt{\frac{2}{3}} J_\mu ^0 + \sqrt{\frac{1}{3}} J_\mu ^8 , \\ \displaystyle J_\mu ^s= \sqrt{\frac{1}{3}} J_\mu ^0 -\sqrt{\frac{2}{3}} J_\mu ^8 , \end{array} \right. \end{aligned}$$

in terms of the usual singlet and octet axial currents previously encountered. Using the results collected in Eq. (66), one can derive:

83 $$\begin{aligned} \left\{ \begin{array}{ll} \displaystyle &{}J^q_\mu /f_\pi = \left[ \frac{\Delta _A}{2}+ \frac{\lambda _q \lambda _3}{2} \right] \partial _\mu \pi ^3_R + \left[ \sqrt{\frac{2}{3}} + \frac{\lambda _q {\widetilde{\lambda }}_0}{2} \right] \partial _\mu \eta _R^0 \\ &{}\qquad \qquad \qquad + \left[ \sqrt{\frac{1}{3}} + \frac{\lambda _q {\widetilde{\lambda }}_8}{2} \right] \partial _\mu \eta _R^8,\\ &{}\displaystyle J^s_\mu /f_\pi = \frac{\lambda _s \lambda _3}{2} \partial _\mu \pi ^3_R + \left[ z_A \sqrt{\frac{1}{3}} + \frac{\lambda _s {\widetilde{\lambda }}_0}{2} \right] \partial _\mu \eta _R^0 \\ &{}\qquad \qquad \qquad - \left[ z_A \sqrt{\frac{2}{3}} - \frac{\lambda _s {\widetilde{\lambda }}_8}{2} \right] \partial _\mu \eta _R^8 , \end{array} \right. \end{aligned}$$

where ${\widetilde{\lambda }}_0$ and ${\widetilde{\lambda }}_8$ have been given in Eq. (25) and where one has defined:

84 $$\begin{aligned} \begin{array}{ll} \displaystyle \lambda _q = \sqrt{\frac{2}{3}} \lambda _0 + \sqrt{\frac{1}{3}} \lambda _8,&\displaystyle \lambda _s = \sqrt{\frac{1}{3}} \lambda _0 - \sqrt{\frac{2}{3}} \lambda _8, \end{array} \end{aligned}$$

in tight connection with the definitions (82). The decay constants relevant in the FKS formulation are:

85 $$\begin{aligned} \left\{ \begin{array}{ll} \displaystyle<0|J_\mu ^q|\eta /{\eta ^\prime }> =iq_\mu F^q_{\eta /{\eta ^\prime }},\\ \displaystyle <0|J_\mu ^s|\eta /{\eta ^\prime }> =iq_\mu F^s_{\eta /{\eta ^\prime }}, \end{array} \right. \end{aligned}$$

and the mixing angles are defined by [37]:

86 $$\begin{aligned} \left\{ \begin{array}{ll} \displaystyle F^q_{\eta } = F_q \cos {\phi _q},&{} F^q_{{\eta ^\prime }} = F_q \sin {\phi _q},\\ F^s_{\eta } = -F_s \sin {\phi _s},&{} F^s_{{\eta ^\prime }} = F_s \cos {\phi _s}. \end{array} \right. \end{aligned}$$

Using Eq. (84) and the definition of the renormalized PS fields in terms of their physical partners (see Eq. (33)), one can derive:

87 $$\begin{aligned} \left\{ \begin{array}{ll} \displaystyle F^q_{\eta }/f_\pi &{}= - \left[ \sqrt{\frac{2}{3}} + \frac{\lambda _q {\widetilde{\lambda }}_0}{2} \right] \sin {\theta _P} \\ &{}\quad + \left[ \sqrt{\frac{1}{3}} + \frac{\lambda _q {\widetilde{\lambda }}_8}{2} \right] \cos {\theta _P} ,\\ \displaystyle F^q_{{\eta ^\prime }}/f_\pi &{}= \left[ \sqrt{\frac{2}{3}} + \frac{\lambda _q {\widetilde{\lambda }}_0}{2} \right] \cos {\theta _P} \\ &{}\quad + \left[ \sqrt{\frac{1}{3}} + \frac{\lambda _q {\widetilde{\lambda }}_8}{2} \right] \sin {\theta _P} ,\\ \displaystyle F^s_{\eta }/f_\pi &{}= - \left[ z_A\sqrt{\frac{1}{3}} + \frac{\lambda _s {\widetilde{\lambda }}_0}{2} \right] \sin {\theta _P} \\ &{}\quad - \left[ z_A \sqrt{\frac{2}{3}} - \frac{\lambda _s {\widetilde{\lambda }}_8}{2} \right] \cos {\theta _P} ,\\ \displaystyle F^s_{{\eta ^\prime }}/f_\pi &{}= \left[ z_A\sqrt{\frac{1}{3}} + \frac{\lambda _s {\widetilde{\lambda }}_0}{2} \right] \cos {\theta _P} \\ &{}\quad - \left[ z_A \sqrt{\frac{2}{3}} - \frac{\lambda _s {\widetilde{\lambda }}_8}{2} \right] \sin {\theta _P}. \end{array} \right. \end{aligned}$$

From Eqs. (86) and (87), one derives:

88 $$\begin{aligned} \left\{ \begin{array}{ll} \displaystyle \tan {\phi _q}&{}= \tan {\left[ \theta _P+U_q\right] }, \displaystyle \tan {U_q}= \frac{\sqrt{\frac{2}{3}}+ \frac{\lambda _q {\widetilde{\lambda }}_0}{2}}{\sqrt{\frac{1}{3}}+ \frac{\lambda _q {\widetilde{\lambda }}_8}{2}} \\ &{}\simeq \sqrt{2} + \frac{3}{2z_A} \lambda _q\lambda _s, \\ \displaystyle \tan {\phi _s}&{}= \tan {\left[ \theta _P+U_s\right] }, \displaystyle \tan {U_s}= \frac{z_A \sqrt{\frac{2}{3}}- \frac{\lambda _s {\widetilde{\lambda }}_8}{2}}{z_A \sqrt{\frac{1}{3}}+ \frac{\lambda _s {\widetilde{\lambda }}_0}{2}}\\ &{} \simeq \sqrt{2} - \frac{3}{2 z_A} \lambda _q\lambda _s , \end{array} \right. \end{aligned}$$

up to terms of order $\mathcal{{O}}(\delta ^2)$. Using the definition of the FKS ideal mixing angle^53^ [37], $\theta _{FKS}= -\arctan {\sqrt{2}}= -54.7^\circ$. Equation (88) implies the following relationships:

89 $$\begin{aligned} \begin{array}{ll} \displaystyle [\phi _q -\phi _s]&{}= \frac{ \lambda _q \lambda _s}{z_A}+\mathcal{{O}}(\delta ^2), \\ \displaystyle [\phi _q +\phi _s]&{}= 2[\theta _P-\theta _{FKS}] +\mathcal{{O}}(\delta ^2) \end{array} \end{aligned}$$

which emphasizes the numerical closeness of the $\phi _q$ and $\phi _s$ FKS mixing angles. It is worthwhile to go on by deriving additional expressions which can be compared to their partners in [35–37]. We have:

90 $$\begin{aligned} \left\{ \begin{array}{ll} \displaystyle [F_q]^2 = [F^q_\eta ]^2 +[F^q_{\eta ^\prime }]^2 = f_\pi ^2 [ 1+\lambda _q^2] + \mathcal{{O}}(\delta ^2) , \\ \displaystyle [F_s]^2 = [F^s_\eta ]^2 +[F^s_{\eta ^\prime }]^2 = f_\pi ^2 [ z_A^2+\lambda _s^2] + \mathcal{{O}}(\delta ^2), \\ F^q_\eta F^s_\eta + F^q_{\eta ^\prime }F^s_{\eta ^\prime }=F_q F_s \sin {[\phi _q-\phi _s]} = f_\pi ^2 \lambda _q \lambda _s + \mathcal{{O}}(\delta ^2) , \end{array} \right. \end{aligned}$$

where $F_s$ and $F_q$ and the FKS mixing angles are given by Eq. (86) above. It is worth remarking that the second Eq. (90) can be rewritten:

$$\begin{aligned} \displaystyle [F_s]^2 =f_\pi ^2[z_A^2+\lambda _s^2] =f_\pi ^2[ 2 z_A -1 +\lambda _s^2 + (z_A-1)^2], \end{aligned}$$

where the last term is second order in SU(3) breaking but not numerically small compared to the $\lambda _0$ or $\lambda _s$ parameter squared values.

Then, using the definitions for our parameters (and canceling out $\lambda _8$), it is obvious that the quantities given by Eq. (90) coincide up to the $\mathcal{{O}}(\delta ^2)$ and $(z_A-1)^2$ terms expected with the corresponding FKS expressions.^54^ Moreover, it should be noted that Eqs. (88) and (89) exhibit the properties of the FKS mixing angles $\phi _q$ and $\phi _s$ emphasized in [37] for instance. In particular, the single-angle $\phi$ occurring in the FKS parameterization is $\phi =[\phi _q +\phi _s]/2$, which only depends on $\theta _P$, whereas the difference $\phi _q - \phi _s$ is a pure effect of the kinetic breaking mechanism defined in Sect. 4.2.

It should thus be noted that the nonvanishing character of $[\phi _q -\phi _s]$ is *not* an isospin breaking effect and that $\phi _q=\phi _s +\mathcal{O}(\delta ^2)$ rather implies either $\lambda _8=-\sqrt{2}\lambda _0$ or $\lambda _0=\sqrt{2}\lambda _8$.

## Further constraining $\hbox {EBHLS}_2$

It may be of interest to identify additional constraints which could apply to $\hbox {EBHLS}_2$ and highlight symmetry breaking effects not explicitly emphasized. In the FKS approach, an important ingredient is some properties of axial currents still not imposed to $\hbox {EBHLS}_2$, namely the diagonal character (at leading order) of the following matrix elements [38]:

91 $$\begin{aligned} \displaystyle <0|J^a_\mu | \eta _a(p)>= & {} ip_\mu f_a \delta _{ab}, \displaystyle |\eta _a(p)> =| a {\overline{a}}(p)>, \nonumber \\ \displaystyle J^a_\mu= & {} {\overline{a}} \gamma _\mu \gamma _5 a, \displaystyle \{a=u,d,s\}. \end{aligned}$$

which may appear to be natural constraints to be plugged into our model where one also works at order $\mathcal{O}(\delta )$. The axial currents relevant for this purpose can be readily derived from those displayed in Eqs. (64) and (82):

92 $$\begin{aligned} \left\{ \begin{array}{ll} \displaystyle J_\mu ^u&{}= \frac{1}{\sqrt{2}} \left[ J_\mu ^q + J_\mu ^3 \right] , \displaystyle J_\mu ^d= \frac{1}{\sqrt{2}} \left[ J_\mu ^q - J_\mu ^3 \right] ,\\ \displaystyle J_\mu ^s&{}= \sqrt{\frac{1}{3}} J_\mu ^0 -\sqrt{\frac{2}{3}} J_\mu ^8, \end{array} \right\} . \end{aligned}$$

As one can identify the leading order term in the Fock expansion of the various $|\eta _a>$ states with the following *bare* PS field combinations:

93 $$\begin{aligned} \left\{ \begin{array}{ll} \displaystyle |\eta _u> =&{} |u {\overline{u}}> = \frac{1}{\sqrt{2}} |\pi ^0_\mathrm{bare}> +\frac{1}{\sqrt{3}} |\eta ^0_\mathrm{bare}> \\ &{} +\frac{1}{\sqrt{6}} |\eta ^8_\mathrm{bare}> , \\ \displaystyle |\eta _d> =&{} |d {\overline{d}}> = - \frac{1}{\sqrt{2}} |\pi ^0_\mathrm{bare}> +\frac{1}{\sqrt{3}} |\eta ^0_\mathrm{bare}> \\ &{}+\frac{1}{\sqrt{6}} |\eta ^8_\mathrm{bare}> , \\ \displaystyle |\eta _s> =&{} |s {\overline{s}}> = ~~\frac{1}{\sqrt{3}} |\eta ^0_\mathrm{bare}> -\sqrt{\frac{2}{3}} |\eta ^8_\mathrm{bare}> , \end{array} \right. \end{aligned}$$

the conditions imposed by Eq. (91) can be accessed within $\hbox {EBHLS}_2$. One thus gets^55^:

94 $$\begin{aligned} \left\{ \begin{array}{ll}\displaystyle \frac{1}{2} \left[ f_u+f_d \right] &{}=\left[ 1 +\frac{\lambda _3^2+\lambda _q^2}{2} \right] f_\pi ,\\ \displaystyle \frac{1}{2} \left[ f_u-f_d \right] &{}= \left[ \lambda _3 \lambda _q +\Delta _A\right] f_\pi , \\ \displaystyle f_s&{}= \left[ z_A^2 + \lambda _s^2\right] f_\pi \end{array} \right\} . \end{aligned}$$

One should note that $(f_u+f_d)/2 \ne f_{\pi ^0}$ if $\lambda _q$ does not identically vanish,^56^ whatever $\lambda _3$ is; it should be emphasized that $\lambda _0$ is related to the so-called $\Lambda _1(\equiv \lambda _0^2)$ of EChPT (see [32–34, 37]). Moreover, the *z* parameter defined by Kroll [38] is:

95 $$\begin{aligned} \displaystyle z_\mathrm{Kroll}= \frac{f_u-f_d}{f_u+f_d}= \Delta _A+\lambda _3 \lambda _q +\mathcal{O}(\delta ^2), \end{aligned}$$

which exhibits the expected dependence upon the isospin breaking parameters of $\hbox {EBHLS}_2$ coming via the $X_A$ and $X_H$ matrices.

On the other hand, the $a\ne b$ matrix elements are (a factor $i f_\pi p_\mu$ being understood):

96 $$\begin{aligned} \left\{ \begin{aligned} \displaystyle<0|J^u_\mu | \eta _d (p)>&= \frac{1}{2} \left[ \lambda _q^2-\lambda _3^2 \right] , \\&\displaystyle<0|J^d_\mu | \eta _u (p)> \\&= \frac{1}{2} \left[ \lambda _q^2-\lambda _3^2 \right] ,\\ \displaystyle<0|J^u_\mu | \eta _s (p)>&= \frac{\lambda _s}{\sqrt{2}} \left[ \lambda _q+\lambda _3 \right] ,\\&\displaystyle<0|J^d_\mu | \eta _s (p)> \\&= \frac{\lambda _s}{\sqrt{2}} \left[ \lambda _q -\lambda _3 \right] ,\\ \displaystyle<0|J^s_\mu | \eta _u (p)> =&\frac{\lambda _s}{\sqrt{2}} \left[ \lambda _q+\lambda _3 \right] ,\\&\displaystyle <0|J^s_\mu | \eta _d (p)> \\&= \frac{\lambda _s}{\sqrt{2}} \left[ \lambda _q -\lambda _3 \right] ,\\ \end{aligned} \right. \end{aligned}$$

Three solutions^57^ allow us to exhaust the simultaneous vanishing of expressions (96); they are:

97 $$\begin{aligned} \left\{ \begin{array}{ll} \displaystyle { \mathrm Solutions} ~~A_\pm : &{} \lambda _s =0, \lambda _q= \pm \lambda _3 \\ &{}\displaystyle \Longrightarrow \lambda _0= \pm \sqrt{\frac{3}{2}} \lambda _3 = \sqrt{2} \lambda _8 , \\ { \mathrm Solution~} ~~B:&{} \displaystyle \lambda _s \ne 0, \lambda _q= \lambda _3 =0 \\ &{}\displaystyle \Longrightarrow \lambda _8= -\sqrt{2} \lambda _0, \lambda _3 =0 , \\ \end{array} \right. \end{aligned}$$

not counting the trivial solution $T \equiv \{ [\lambda _0= \lambda _8= \lambda _3 =0]\}$, already known to be unable to satisfactorily accommodate our set of reference data – this statement is also valid for solution B as shown in the first data column of Table 1 in connection with the account of the Belle dipion spectrum [21]. In contrast, both solutions $A_\pm$ are found to work well within our minimization procedure. For these solutions, the three parameters of the kinetic breaking mechanism are no longer free – as assumed in the preceding sections in line with the common belief – but become algebraically related to each other.

So, it follows from the developments just stated that imposing the Kroll conditions (91) is far from anecdotal; indeed, any of the solutions (97) which cancel out the matrix elements in Eq. (96) shows that a nonvanishing $\lambda _0$ (the usual kinetic ’t Hooft determinant term) is possible *if and only if* $\lambda _8$ is nonzero. This statement – valid if defining $X_H$ by Eq. (20) – also applies if one prefers^58^ defining $X_H$ by Eq. (22). It should also be stressed that only one of the previously defined solutions can be valid; it could, hopefully, be identified by confronting each solution with the data.

## The $\pi ^0-\eta -{\eta ^\prime }$ mixing: breaking of isospin symmetry

Another topic relevant for the $\pi ^0-\eta -{\eta ^\prime }$ mixing is the content of isospin zero mesons inside the physically observed $\pi ^0$ wave-function; accounts of this can be found in [37, 38] for instance. In standard ChPT approaches, the *physical* $\pi ^0$ is expressed in terms of the *bare* $\pi ^0_{bare}$ field with admixtures of the *physical* $\eta$ and ${\eta ^\prime }$ mesons:

98 $$\begin{aligned} \displaystyle |\pi ^0>=|\pi ^0_\mathrm{bare}> +\kappa |\eta> +\kappa ^\prime |{\eta ^\prime }> + \mathcal{O}(\delta ^2), \end{aligned}$$

the $\mathcal{O}(\delta )$ parameters $\kappa$ and $\kappa ^\prime$ depending on the light quark mass difference take respectively the values $\kappa _0$ and $\kappa _0^\prime$ defined by [37]:

99 $$\begin{aligned} \displaystyle \kappa _0= & {} \frac{1}{2} \cos {\phi } \frac{m^2_{dd} -m^2_{uu} }{M_\eta ^2 -M_\pi ^2},\quad \kappa _0^\prime = \frac{1}{2} \sin {\phi } \frac{m^2_{dd} -m^2_{uu} }{M_{\eta ^\prime }^2 -M_\pi ^2} , \end{aligned}$$

up to higher-order contributions.^59^ The quark mass term can be estimated by^60^$m^2_{dd} -m^2_{uu}= 2 [M_{K^0}^2- M_{K^\pm }^2-M_{\pi ^0}^2+M_{\pi ^\pm }^2] \simeq 1.03 \times 10^{-2}$ $\hbox {GeV}^2$, and $\phi$ is some approximate value derived from the $\phi _s$ and $\phi _q$ angles defined in Sect. 15. However, because $\phi _q -\phi _s \simeq \lambda _q \lambda _s +\mathcal{O}(\delta ^2)$, any solution providing the vanishing of Eq. (96) automatically provides $\phi _s=\phi _q +\mathcal{O}(\delta ^2)$.

On the other hand, Kroll has extended this formulation [38] in order to account for isospin breaking effects not generated by the light quark mass difference:

100 $$\begin{aligned} \begin{array}{ll} \displaystyle \kappa &{}= \cos {\phi } \left[ \frac{m^2_{dd} -m^2_{uu} }{2(M_\eta ^2 -M_\pi ^2)} +z_{Kroll} \right] ,\\ \displaystyle \kappa ^\prime &{}= \sin {\phi } \left[ \frac{m^2_{dd} -m^2_{uu} }{2(M_{\eta ^\prime }^2 -M_\pi ^2)}+z_{Kroll} \right] , \end{array} \end{aligned}$$

where $z_{Kroll}$ is expressed in terms of the $f_u$ and $f_d$ decay constants defined by Eq. (91) and expressed in the $\hbox {EBHLS}_2$ framework by Eq. (95).

In order to connect $\hbox {EBHLS}_2$ with the $\eta /{\eta ^\prime }$ fractions inside the physically observed $\pi ^0$ [38, 39], one needs the relation involving these and $\pi ^0_{bare}$. After some algebra, Eqs. (31) and (33) allow us to derive an expression similar to Eq. (98):

101 $$\begin{aligned} \displaystyle | \pi ^0> =\left[ 1+\frac{\lambda _3^2}{2} \right] |\pi ^0_{bare}> +\varepsilon |\eta> +\varepsilon ^\prime |{\eta ^\prime }> , \end{aligned}$$

where the rescaling of the $\pi ^0_\mathrm{bare}$ term is specific to the kinetic breaking $X_H$ introduced in the $\hbox {EBHLS}_2$ Lagrangian, and $\varepsilon$ and $\varepsilon ^\prime$ are given by:

102 $$\begin{aligned} \left\{ \begin{array}{lll} \displaystyle \varepsilon \!=\!\epsilon +\frac{\lambda _3 \lambda _q +\Delta _A}{2} \cos {\left( \frac{\phi _q+\phi _s}{2}\right) }\! -\!\frac{\lambda _3 \lambda _s}{2 z_A} \sin {\left( \frac{\phi _q+\phi _s}{2}\right) }, \\[3pt] \displaystyle \varepsilon ^\prime =\! \epsilon ^\prime + \frac{\lambda _3 \lambda _q\! +\! \Delta _A}{2} \sin {\left( \frac{\phi _q+\phi _s}{2}\right) } \!+\!\frac{\lambda _3 \lambda _s}{2 z_A} \cos {\left( \frac{\phi _q+\phi _s}{2}\right) } , \end{array} \right. \end{aligned}$$

up to terms of order $\mathcal{O}(\delta ^2)$, having used Eq. (89) and defined $\theta _{FKS}=-\arctan {\sqrt{2}}$.

As $(\phi _q+\phi _s)/2$ is certainly a quite motivated expression for the FKS parameter $\phi$ [37, 38], the similarity of Eqs. (100) and (102) is striking, and even more if imposing $\lambda _s=0$ – as requested by any of the $A_\pm$ solutions (see Eq. (97)) – which drops the last term in each of Eq. (102). The condition $\lambda _s=0$, indeed, implies:

103 $$\begin{aligned}&\left\{ \displaystyle \varepsilon = \epsilon +\frac{\lambda _3 \lambda _q + \Delta _A}{2} \cos {\phi } ,\right. \nonumber \\&\quad \left. \varepsilon ^\prime = \epsilon ^\prime + \frac{\lambda _3 \lambda _q + \Delta _A}{2} \sin {\phi } \right\} . \end{aligned}$$

with $\phi =\phi _q=\phi _s$ up to $\mathcal{O}(\delta ^2)$ terms. Switching off the BKY ($\Delta _A$) and kinetic breaking mechanisms turns out to set $f_u=f_d$, and then one expects to recover the results usual in this limit [37, 38]. Thus, the following identifications

104 $$\begin{aligned}&\left\{ \displaystyle \epsilon = \frac{1}{2} \cos {\phi } \frac{m^2_{dd} -m^2_{uu} }{M_\eta ^2 -M_\pi ^2}~~~ (=\kappa _0) , \right. \nonumber \\&\left. ~~\epsilon ^\prime = \frac{1}{2} \sin {\phi } \frac{m^2_{dd} -m^2_{uu} }{M_{\eta ^\prime }^2 -M_\pi ^2} ~~~(=\kappa _0^\prime ) \right\} \end{aligned}$$

appear motivated. However, because additional singlets – like a gluonium – may contribute to the $\eta -{\eta ^\prime }$ mixing, likely more inside the ${\eta ^\prime }$ meson than inside $\eta$, it is of concern to allow for a departure from the mere identification (104), especially for the ${\eta ^\prime }$ amplitude term $\epsilon ^\prime$. So, letting $\epsilon$ and $\epsilon ^\prime$ float independently provides a relevant piece of information.

$\kappa _0$ and $\kappa _0^\prime$ are a common way to express isospin breaking effects due to quark masses in the FKS picture; another way to proceed is proposed in [39], which was used in our previous works. This turns out to rely on quark masses, defined as:105and replace Eq. (104) by:

106 $$\begin{aligned} \begin{array}{lll} \displaystyle \{ \epsilon =\varpi _0,&~~\epsilon ^\prime =\varpi _0^\prime \}. \end{array} \end{aligned}$$

The angle $\delta _P$ occurring in these expressions, defined in Eq. (112), is given by $\delta _P=\theta _P -\theta _I$, where $\theta _I=\pi /2 -\theta _{FKS}$; in this approach, the floating parameter is no longer $m^2_{dd} -m^2_{uu}$, but $\varepsilon _0$. Using the light quark masses from FLAG 2016 [112], $\varepsilon _0$ is expected to be around $\simeq 1.22\times 10^{-2}$. One can anticipate fit results and state that fitting with Eqs. (104) or (105) yields similar fit properties.

Finally, we should mention that the $z_{Kroll}$ dependence in Eqs. (102) and (103) exhibits an unexpected difference compared to Eq. (100): $\hbox {EBHLS}_2$ finds a weight for $z_{Kroll}$ smaller by a factor of 2. Whether it is a specific feature of $\hbox {EBHLS}_2$ is an open question.

## The $\pi ^0-\eta -{\eta ^\prime }$ mixing: the $\hbox {EBHLS}_2$ analysis

In order to deal with the $\tau$ dipion spectra and the update of the muon HVP, it was found appropriate to release at most the constraints on the model parameters within the fit procedure; this also applies to the model parameters named $\epsilon$ and $\epsilon ^\prime$ which were left free to vary independently.

In order to compare with expectations, it is also worthwhile to consider the case when $\epsilon =\kappa _0$ and $\epsilon ^\prime =\kappa _0^\prime$ are imposed; this turns out to let the parameter $m^2_{dd} -m^2_{uu}$ float and derive $\epsilon$ and $\epsilon ^\prime$ by means of Eq. (104), which constrains $\epsilon$ and $\epsilon ^\prime$ to be like-sign. Instead, if $\epsilon$ and $\epsilon ^\prime$ are floating independently, Eq. (104) allows for separate determinations of $m^2_{dd} -m^2_{uu}$ from the fitted $\eta$ and ${\eta ^\prime }$ admixtures.

**Table 9:** Selected individual $\chi ^2/N_\mathrm{pts}$ values in $\hbox {EBHLS}_2$ fits versus the Kroll conditions (cf Eq. (91)). The first data column reports on the fit where the three $\lambda$’s vary independently; the others refer to the solutions defined in Eq. (97). The leftmost four data columns assume condition *C* (see text), whereas condition *C* has been relaxed in the last two column fits. The last lines display the global $\chi ^2/N_{\mathrm{pts}}$ and probability for each fit

$\hbox {EBHLS}_2$ (BS)	Sol. F	Sol. *T*/*B*	Sol. $A_+$	Sol. $A_-$	Sol. $A_+$	Sol. $A_-$
	$\epsilon =\kappa _0$ and $\epsilon ^\prime =\kappa _0^\prime$				$\epsilon ~~and ~~\epsilon ^\prime$ free	
NSK $\pi ^+ \pi ^-$ (127)	139	134	132	136	136	138
KLOE $\pi ^+ \pi ^-$ (135)	137	153	143	139	141	138
BESIII $\pi ^+ \pi ^-$ (60)	49	48	47	49	48	49
Spacelike $\pi ^+ \pi ^-$ (59)	61	64	61	59	60	59
$\tau$ (A+C) (66)	61	75	60	59	65	61
$\tau$ (B) (19)	28	53	32	27	35	28
$\pi ^0 \gamma$ (112)	86	98	86	94	86	92
$\eta \gamma$ (182)	123	132	131	120	125	120
NSK $\pi ^+ \pi ^-\pi ^0$ (158)	149	158	154	150	150	149
BESIII $\pi ^+ \pi ^-\pi ^0$ (128)	138	138	138	138	138	138
$P\gamma \gamma$ & ${\eta ^\prime }V \gamma$ (5)	5	8	8	9	4	7
$\chi ^2/N_{\mathrm{pts}}$	1280/1366	1375/1366	1309/1366	1289/1366	1292/1366	1286/1366
Probability	85.9%	23.3 %	70.6 %	82.5%	80.1 %	83.5%

Furthermore, in the fits reported from now on, the polynomial $\delta P^\tau (s)$ is always second degree and, for completeness, we allow the $\Delta _A$ isospin breaking parameter to float, even if it is not really significant – never more than $2\sigma$.

The PDG value for the ratio $f_K/f_\pi$ is included in the set of experimental data submitted to fit. The Belle dipion spectrum is included, and we refer the reader to Sect. 3 for the specific consequences this implies for the dipion spectra collected in the $\tau$ lepton decay.

To be as comprehensive as possible, several cases for the $[\lambda _0, \lambda _8,\lambda _3]$ triplet have been considered, namely the $A_\pm$ and *B* solutions defined in Eq. (97), as well as the so-called trivial solution $\{T \equiv [\lambda _0= \lambda _8= \lambda _3 =0]\}$; it has been found worthwhile to also consider the case when the three $\lambda$ parameters are left floating independently – referred to hereafter as solution F. Solution F is, actually, very similar to the fit conditions of the previous sections. The main fit properties are gathered in Table 9 and lead to the following:

- Regarding solution *B*, the best fit returns $\lambda _0= (-0.01 \pm 36.26) \times 10^{-2}$, which clearly exhibits convergence towards the trivial solution $\{T \equiv [\lambda _0= \lambda _8= \lambda _3 =0]\}$; therefore, there is no point in distinguishing solution *B* from the trivial solution *T*, which is the one actually reported.
- With a minimum total $\chi ^2$ larger by 60–95 units than the other solutions, solution *T*/*B* can be safely discarded.
- When assuming condition
  $$\begin{aligned} C \equiv \{\epsilon = \kappa _0, \epsilon ^\prime = \kappa _0^\prime \} \end{aligned}$$
  both solutions $A_\pm$ return good probabilities. Solution $A_-$ is, however, clearly favored even if $A_+$ exhibits a reasonable goodness of fit. Nevertheless, relaxing condition *C*, solutions $A_\pm$ exhibit practically the same fit probability. This indicates that condition *C* is not a real constraint for solution $A_-$, whose total $\chi ^2$ is almost unchanged ($\Delta \chi ^2=3$). In contrast, condition *C* exhibits a strong tension with solution $A_+$ and provides a strongly degraded fit as $\Delta \chi ^2=17$ for only one fewer parameter; however, $\epsilon$ and $\epsilon ^\prime$ become unlike signs when relaxing condition *C*, which is certainly inconsistent with Eq. (104) – or Eq. (105) – and with common expectations. One observes, nevertheless, that the decay information (at the bottom of Table 9) are better described^61^ by solution $A_+$ than solution $A_-$. It should be noted that most studies of the $[\pi ^0,\eta ,{\eta ^\prime }]$ mixing properties simply rely on the two-body decays with $P\gamma \gamma$ and $PV\gamma$ couplings.

- Replacing condition *C* by
  $$\begin{aligned} C^\prime \equiv \{\epsilon = \varpi _0, \epsilon ^\prime = \varpi _0^\prime \} \end{aligned}$$
  does not lead to substantial differences. Indeed, one gets $\chi ^2/N_{\mathrm{pts}}=1303/1366$ and 74.0% probability ($A_+$) or $\chi ^2/N_{\mathrm{pts}}=1286/1366$ and 83.8% probability ($A_-$), i.e., solution $A_-$ remains preferred to $A_+$ by the data. It should be noted that relaxing condition *C* (or $C^\prime$) leads to like-sign $\epsilon$ and $\epsilon ^\prime$ for solution $A_-$, but to unlike signs for solution $A_+$. As just noted, this could motivate the discarding of solution $A_+$.
- As could be expected, solution *F* is also good, benefiting from larger parameter freedom than $A_\pm$ whether submitted to condition *C* or not.

**Table 10:** Model parameter values from the fits performed within $\hbox {EBHLS}_2$ under the various configurations defined in the text. Angles are in degrees, $[m_{dd}^2- m_{uu}^2]$ in $\hbox {GeV}^{2}$. Values in boldface are not floating but derived from the floating parameters through equations given in the main text; their uncertainties are likewise derived and take into account the parameter covariance matrix

$\hbox {EBHLS}_2$ (BS)	Sol. F	Sol. *T*/*B*	Sol. $A_+$	Sol. $A_-$	Sol. $A_+$	Sol. $A_-$
	$\epsilon =\kappa _0$ and $\epsilon ^\prime =\kappa _0^\prime$				$\epsilon$ and $\epsilon ^\prime$ free	
*g*	$6.996\pm 0.002$	$7.052\pm 0.002$	$6.536 \pm 0.002$	$6.671\pm 0.001$	$7.069\pm 0.002$	$6.954\pm 0.002$
$a_{\mathrm{HLS}}$	$1.764\pm 0.001$	$1.646\pm 0.001$	$1.728 \pm 0.001$	$1.765\pm 0.001$	$1.752\pm 0.001$	$1.766\pm 0.001$
$(c_3+c_4)/2$	$0.756 \pm 0.004$	$0.739 \pm 0.003$	$0.769\pm 0.004$	$0.742 \pm 0.003$	$0.769\pm 0.004$	$0.742\pm 0.003$
$c_1-c_2$	$0.762 \pm 0.014$	$0.775\pm 0.012$	$0.766 \pm 0.012$	$0.823 \pm 0.013$	$0.676 \pm 0.013$	$0.809 \pm 0.013$
$10^2\times z_3$	$-0.332 \pm 0.004$	$-0.364 \pm 0.030$	$-0.372 \pm 0.004$	$-0.354 \pm 0.004$	$-0.345 \pm 0.004$	$-0.339 \pm 0.004$
$10^2\times [m_{dd}^2- m_{uu}^2]$	$2.65 \pm 0.25$	$3.78 \pm 0.13$	$2.49 \pm 0.15$	$3.01 \pm 0.14$	$\times$	$\times$
$10^2\times \epsilon$	$\mathbf{3.67 \pm 0.32}$	$\mathbf{5} .\mathbf{19}$	$\mathbf{3.48 \pm 0.20}$	$\mathbf{4.16 \pm 0.20}$	$2.28 \pm 0.30$	$3.62 \pm 0.30$
$10^2\times \epsilon ^\prime$	$\mathbf{0.93 \pm 0.09}$	$\mathbf{1} .\mathbf{34}$	$\mathbf{0.86 \pm 0.05}$	$\mathbf{1.05 \pm 0.05}$	$-1.20 \pm 0.30$	$0.17 \pm 0.27$
$\theta _P$	$-15.89\pm 0.34$	$-15.36 \pm 0.28$	$-16.63 \pm 0.30$	$-15.78 \pm 0.28$	$-16.63 \pm 0.30$	$-15.59 \pm 0.28$
$z_A$	$1.417\pm 0.004$	$1.411 \pm 0.004$	$1.429 \pm .004$	$1.405\pm 0.004$	$1.423\pm 0.004$	$1.406\pm 0.004$
$z_V$	$1.433 \pm 0.001$	$1.507 \pm 0.001$	$1.463 \pm 0.001$	$1.419 \pm 0.001$	$1.436 \pm 0.001$	$1.420 \pm 0.001$
$10^2\times \Delta _A$	$0.12\pm 5.09$	$10.93\pm 5.05$	$-8.71 \pm 5.16$	$12.65\pm 5.14$	$-6.82\pm 5.23$	$12.94\pm 4.91$
$\lambda _3$	$0.236 \pm 0.007$	$\mathbf{\equiv 0}$	$\mathbf{0.212 \pm 0.007}$	$\mathbf{-0.242 \pm 0.007}$	$\mathbf{0.197 \pm 008}$	$\mathbf{-0.233 \pm 0.007}$
$\lambda _0$	$0.152\pm 0.042$	$\mathbf{\equiv 0}$	$0.259 \pm 0.009$	$0.295\pm 0.009$	$0.241\pm 0.009$	$0.285\pm 0.009$
$\lambda _8$	$0.022\pm 0.023$	$\mathbf{\equiv 0}$	$\mathbf{0.183 \pm 0.006}$	$\mathbf{0.209\pm 0.006 }$	$\mathbf{0.170\pm 0.007 }$	$\mathbf{0.202\pm 0.006}$
$10^2 \times \xi _0$	$-7.237\pm 0.019$	$-5.130 \pm 0.030$	$-0.022 \pm 0.027$	$-3.114\pm 0.020$	$-7.809\pm 0.019$	$-6.838\pm 0.018$
$10^2 \times \xi _3$	$2.231 \pm 0.155$	$-3.598 \pm 0.072$	$3.155 \pm 0.129$	$3.034 \pm 0.148$	$0.599 \pm 0.136$	$1.496 \pm 0.150$
$\chi ^2/N_{\mathrm{pts}}$	1280/1366	1375/1366	1309/1366	1289/1366	1292/1366	1286/1366
Probability	85.9%	23.3 %	70.6%	82.5%	80.1%	83.5%

The model parameter values returned by the various fits are displayed in Table 10. One can observe that the specific HLS model parameters do not vary much depending on the solution examined; this is indeed so for *g*, $a (\equiv a_{HLS})$, $z_3$, $c_1-c_2$ and for^62^$(c_3+c_4)/2$. This is also observed for the BKY breaking parameters $z_A$ and $z_V$, whereas $\Delta _A$ is clearly not significant. In contrast, $\xi _0$ undergoes a surprisingly large change when going from $A_+$ to $A_-$. The value for $\xi _3$ strongly depends on whether condition *C* is required, but is similar for solutions $A_+$ and $A_-$ in the former case.

The parameter equivalent to the so-called $\Lambda _1$ [32, 33, 37] ($\Lambda _1 =\lambda _0^2$) is found in the range from 6.5% ($A_+$) to 8.5% ($A_-$). However, it should be stressed that, once assuming the Kroll conditions (91), it cannot come alone as reflected by Eq. (97) and determined by our fits. Their numerical values are marginally affected by condition *C* or by choosing $A_+$ or $A_-$ – up to the sign for $\lambda _3$.

Therefore, an important piece of information should be stressed: Because of the strict relation between $\lambda _3$ and $\lambda _0$ – and hence $\Lambda _1$ – the Kroll conditions (91) imply that the pion form factor in the $\tau$ decay fulfills $F^\tau _\pi (0)= 1-\lambda _3^2/2$ and, then, is no longer unity, as inferred at the beginning of the present study.

## Side results from fits

**Table 11:** Singlet-octet and quark flavor bases mixing parameter values derived from fits performed within $\hbox {EBHLS}_2$ under the *F* and $A_\pm$ configurations defined in the text. For the configuration *F*, $\phi =(\phi _q+\phi _s)/2$ whereas $\phi =\phi _q ~(=\phi _s)$ for the $A_\pm$ solutions. Correspondingly, the contributions of the HLS channels for $\sqrt{s}\le 1.05$ GeV to the HVP are also given in each case; they can be compared to Table 7. The main fit properties are noted at the bottom end of the Table

$\hbox {EBHLS}_2$ (BS)	Sol. F	Sol. $A_+$	Sol. $A_-$	Sol. $A_+$	Sol. $A_-$
	$\epsilon =\kappa _0$ and $\epsilon ^\prime =\kappa _0^\prime$			$\epsilon and ~~\epsilon ^\prime$ free	
$\theta _P$ (deg.)	$-15.89\pm 0.34$	$-16.63 \pm 0.30$	$-15.78 \pm 0.28$	$-16.63 \pm 0.30$	$-15.59 \pm 0.28$
$\theta _0$ (deg.)	$-6.35\pm 0.47$	$-8.04\pm 0.39$	$-8.05\pm 0.33$	$-7.95 \pm 0.39$	$-7.71 \pm 0.34$
$\theta _8$ (deg.)	$-24.55\pm 0.30$	$-24.44\pm 0.25$	$-22.83\pm 0.27$	$-24.50 \pm 0.25$	$-22.77 \pm 0.28$
$\theta _0-\theta _8$ (deg.)	$18.21\pm 0.24$	$16.45\pm 0.23$	$14.85\pm 0.24$	$16.59\pm 0.23$	$15.13\pm 0.24$
$F_0/f_\pi$	$1.166 \pm 0.006$	$1.190 \pm 0.003$	$1.190 \pm 0.003$	$1.184 \pm 0.003$	$1.187 \pm 0.003$
$F_8/f_\pi$	$1.293 \pm 0.003$	$1.315 \pm 0.003$	$1.302 \pm 0.003$	$1.309 \pm 0.003$	$1.302 \pm 0.003$
FKS $\phi$ (deg.)	$38.85 \pm 0.35$	$38.96 \pm 0.27$	$38.08 \pm 0.29$	$38.09 \pm 0.30$	$39.15 \pm 0.27$
$\phi _q-\phi _s$ (deg.)	$0.39 \pm 0.18$	$\equiv 0$	$\equiv 0$	$\equiv 0$	$\equiv 0$
$F_q/f_\pi$	$1.008 \pm 0.007$	$1.050 \pm 0.003$	$1.066 \pm 0.004$	$1.044 \pm 0.003$	$1.061 \pm 0.004$
$F_s/f_\pi$	$1.418 \pm 0.005$	$1.428 \pm 0.004$	$1.405 \pm 0.004$	$1.423 \pm 0.004$	$1.406 \pm 0.004$
$F_s/f_K$	$1.192 \pm 0.003$	$1.198 \pm 0.003$	$1.181 \pm 0.002$	$1.195 \pm 0.002$	$1.182 \pm 0.002$
$f_K/f_\pi$	$1.190 \pm 0.002$	$1.193 \pm 0.002$	$1.189 \pm 0.002$	$1.191 \pm 0.002$	$1.190 \pm 0.002$
$10^{10}\times a_\mu (HLS)$	$572.52 \pm 1.02$	$571.84 \pm 0.98$	$572.44 \pm 0.98$	$575.00 \pm 0.95$	$572.59 \pm 0.99$
$\chi ^2/N_{\mathrm{pts}}$	1280/1366	1309/1366	1289/1366	1292/1366	1286/1366
Probability	85.9%	70.6%	82.5%	80.1%	83.5%

Table 11 collects our main results, mostly related to the $\pi ^0-\eta -{\eta ^\prime }$ mixing parameter evaluations. However, it is worthwhile to include some topical pieces of information which deserve special emphasis.

- The various estimates for $f_K/f_\pi$ displayed in Table 11 nicely compare to LQCD determinations, namely [112] $1.195 \pm 0.005$ and [113] $1.1995 \pm 0.0044$.

**Table 12:** Mixing parameters in the singlet-octet and quark flavor bases from various sources. The $\hbox {EBHLS}_2$ evaluations displayed are the average values derived for solutions $A_+$ and $A_-$ assuming condition *C*, whereas the second uncertainty is half their difference; the original $A_+$ and $A_-$ are given in Table 11. The data derived by other groups are FKS 98 [36], EF 05 [114], EGMS 15b [115] and the LQCD results OU 17 [116]; the number within parentheses is from EMS 15 [117]. Angles are expressed in degrees

	$\hbox {EBHLS}_2$ avrg.	FKS 98	EF 05	EGMS 15b	OU 17
$\theta _0$	$-8.04\pm 0.39 \pm 0.00$	$-9.2 \pm 1.7$	$-2.4\pm 1.9$	$-6.9 \pm 2.4$	$\times$
$\theta _8$	$-23.64\pm 0.30 \pm 0.27$	$-21.2\pm 1.6$	$-23.8\pm 1.4$	$-21.2 \pm 1.9$	$\times$
$F_0/f_\pi$	$1.190 \pm 0.003 \pm 0.000$	$1.17 \pm 0.03$	$1.29 \pm 0.04$	$1.14 \pm 0.05$	$\times$
$F_8/f_\pi$	$1.309 \pm 0.003 \pm 0.007$	$1.26 \pm 0.04$	$1.51 \pm 0.05$	$1.27 \pm 0.02$	$\times$
$\phi$	$38.52 \pm 0.29 \pm 0.44$	$39.3 \pm 1.0$	$41.4 \pm 1.4$	($38.3 \pm 1.6$)	$39.8 \pm 2.2 \pm 2.4$
$F_q/f_\pi$	$1.058 \pm 0.004 \pm 0.008$	$1.07 \pm 0.02$	$1.09\pm 0.03$	$1.03 \pm 0.04$	$0.960 \pm 0.037 \pm 0.046$
$F_s/f_\pi$	$1.417 \pm 0.004 \pm 0.012$	$1.34 \pm 0.06$	$1.66 \pm 0.06$	$1.36 \pm 0.04$	$1.363 \pm 0.27 \pm 0.006$
$F_s/f_K$	$1.190 \pm 0.003 \pm 0.009$	$\times$	$\times$	$\times$	$1.143 \pm 0.023 \pm 0.005$

- The pion and kaon charge radii given in Table 5 of [19] remain unchanged within the $\hbox {EBHLS}_2$ framework; they were observed in fair accord with expectations.
- The values derived for the muon HVP contribution $a_\mu (HLS)$ of the (6) annihilation channels embodied inside the $\hbox {EBHLS}_2$ framework and integrated up to $\sqrt{s}= 1.05$ GeV are also shown and can be compared with the corresponding information in Table 7. The reference evaluation reported there from a fit using a least constrained $\hbox {EBHLS}_2$ variant was:
  $$\begin{aligned} a_\mu (HLS, \sqrt{s}= 1.05~\mathrm{GeV}) = [571.97\pm 0.95] \times 10^{-10}, \end{aligned}$$
  which – accidentally – coincides with the average value derived using $A_+$ and $A_-$ under condition *C*. In this case, the $\hbox {EBHLS}_2$ variants fulfilling the Kroll conditions (91) and condition *C* do not depart from the average estimate by more than $\simeq \pm 0.3 \times 10^{-10}$; this can be conservatively taken as the model uncertainty affecting our evaluation of $a_\mu (HLS)$ as, moreover, taking into account the mixing properties of the $\pi ^0-\eta -{\eta ^\prime }$ discussed in Sect. 17, it looks natural to impose condition C to $\hbox {EBHLS}_2$. Finally, as noted in the preceding Sect. 18, the closeness observed between solutions F and $A_-$ leads us to conclude that condition C is an intrinsic feature of solution $A_-$, a nice property not shared by $A_+$; this leads us to favor solution $A_-$ over solution $A_+$.

Releasing, for completeness, condition C exhibits interesting results concerning $\epsilon$ and $\epsilon ^\prime$. In this case, solution $A_-$ returns like-sign $\epsilon$ and $\epsilon ^\prime$ – as expected from Eq. (104)– whereas solution $A_+$ returns unlike sign values and a significant shift^63^ upward of $\Delta a_\mu (HLS) =574.83-571.97=2.86$ in units of $10^{-10}$. The unlike sign character of $\epsilon$ and $\epsilon ^\prime$, contradicting the expected properties of the $\pi ^0-\eta -{\eta ^\prime }$ mixing, also disfavors solution $A_+$ over solution $A_-$.

## Evaluations of the $\pi ^0-\eta -{\eta ^\prime }$ mixing parameters

Table 11 displays the parameter values derived by fitting our set of data within $\hbox {EBHLS}_2$ under the various solutions to Eq. (91). We have found it interesting to also produce the results derived assuming the $\lambda _i$ unconstrained (the so-called solution F). One can observe a fair stability of the usual mixing parameters, as the spread of values is very limited for each of them.

It is, of course, worth comparing our results with other determinations. For this purpose, we have selected a limited set of data and refer the reader to the corresponding papers to track back to former references; the comparison can be easily performed by looking at Table 12.

In order to facilitate the comparisons, the first data column in Table 12 displays the averages of the values derived using solutions $A_+$ and $A_-$ under condition *C* which can be found in Table 11; half their difference is given as an estimate of the systematic uncertainty and shown as the second error.

The agreement is clearly satisfactory with FKS 98 [36] – based on meson decays involving $P\gamma \gamma$ and $J/\psi$ decays to $\eta$ and ${\eta ^\prime }$. EF 05 [114] produces several parameter values depending on the information implemented. For instance, also using the $P \rightarrow \gamma \gamma$ and $J/\psi \rightarrow (\eta /{\eta ^\prime }) \gamma$ decays only, together with the ChPT prediction $F_8=1.28 f_\pi$, Escribano and Frère derive:

107 $$\begin{aligned}&\left\{ \theta _8 =(-22.2 \pm 1.8)^\circ , \displaystyle \theta _0 =(-8.7 \pm 2.1)^\circ , \right. \nonumber \\&\qquad \left. F_0 /f_\pi = 1.18 \pm 0.04 \right\} , \end{aligned}$$

in very good agreement with FKS 98 and $\hbox {EBHLS}_2$. Introducing, in addition, a parameterization^64^ of the coupling constants $(\eta /{\eta ^\prime })V\gamma$, where $V=\rho ^0,\omega ,\phi$, they can use the corresponding tabulated decays widths to produce the numbers displayed in the third data column of Table 12. As for the singlet-octet parameters, the comparison with others is not as satisfactory; nevertheless, the quark flavor scheme parameters compare reasonably well.

Analyzing the asymptotic behavior of the $\eta /{\eta ^\prime }$ meson transition form factors $F_{(\eta /{\eta ^\prime }) \gamma ^* \gamma } (Q^2)$ and using the Padé approximant method, EMS 15 [117] derive two solutions; that based on the asymptotics of $F_{\eta \gamma ^* \gamma }(Q^2)$ is in good accord with our results, and the value for the $\phi$ angle is displayed in Table 12. The solution based on the $F_{{\eta ^\prime }\gamma ^* \gamma }(Q^2)$ asymptotics, improved soon after, is given in Table 12 under the tag EGMS 15b [115]; their evaluations are in good accord with ours, as well as with those in FKS  98. On the other hand, they also obtain:

$$\begin{aligned} \phi _q = [39.6 \pm 2.3]^\circ ~~~\mathrm{and}~~~\phi _s = [40.8 \pm 1.8]^\circ , \end{aligned}$$

which are consistent with $\phi _q = \phi _s$ at a $1 \sigma$ level. Finally, the ETM Lattice QCD Collaboration has derived the numbers given in the last data column tagged OU 17 [116]. Our results are consistent with these LQCD evaluation at the $\simeq 1 \sigma$ level.

One more piece of information can be of interest which could mimic higher-order effects. Using solution *F*, which slightly violates the Kroll conditions, $\phi _q$ and $\phi _s$ become slightly different; they allow us to derive:

108 $$\begin{aligned} \displaystyle \frac{\phi _q-\phi _s}{\phi _q + \phi _s} =[0.50 \pm 0.24] \times 10^{-2}<< 1 \end{aligned}$$

as expected.

**Table 13:** Isospin breaking effects within $\hbox {EBHLS}_2$ using condition *C* to relate $\epsilon$ and $\epsilon ^\prime$. See text for definitions and notations. The entry for $[m_{dd}^2- m_{uu}^2]$ is in $\hbox {GeV}^2$. The main fit properties are noted at the bottom end of the Table

$\hbox {EBHLS}_2$ (BS)	Sol. F	Sol. $A_+$	Sol. $A_-$	Sol. $A_+$	Sol. $A_-$
	$\epsilon =\kappa _0$ and $\epsilon ^\prime =\kappa _0^\prime$			$\epsilon$ and $\epsilon ^\prime$ free	
$10^2\times [m_{dd}^2- m_{uu}^2]$	$2.65 \pm 0.25$	$2.49 \pm 0.15$	$3.01 \pm 0.14$	$\times$	$\times$
$10^2\times \epsilon$	$3.67 \pm 0.32$	$3.48 \pm 0.20$	$4.16 \pm 0.20$	$2.28 \pm 0.30$	$3.62 \pm 0.30$
$10^2\times \epsilon ^\prime$	$0.93 \pm 0.09$	$0.86 \pm 0.05$	$1.05 \pm 0.05$	$-1.20 \pm 0.30$	$0.17 \pm 0.27$
$10^2\times \varepsilon$	$4.92 \pm 0.37$	$5.80 \pm 0.31$	$1.24 \pm 0.32$	$4.33 \pm 0.34$	$0.95 \pm 0.36$
$10^2\times \varepsilon ^\prime$	$1.94 \pm 0.26$	$2.66 \pm 0.18$	$-1.30 \pm 0.23$	$0.40 \pm 0.28$	$-2.00 \pm 0.32$
$f_u/f_\pi$	$1.070 \pm 0.015$	$1.131 \pm 0.009$	$1.020 \pm 0.005$	$1.114 \pm 0.009$	$1.020 \pm 0.005$
$f_d/f_\pi$	$1.006 \pm 0.006$	$1.014 \pm 0.005$	$1.170 \pm 0.012$	$1.012 \pm 0.005$	$1.157 \pm 0.012$
$10^2\times z_{Kroll}$	$3.24 \pm 0.95$	$5.86 \pm 0.58$	$-7.49 \pm 0.72$	$5.13 \pm 0.58$	$-6.86 \pm 0.69$
$\chi ^2/N_{\mathrm{pts}}$	1280/1366	1309/1366	1289/1366	1292/1366	1286/1366
Probability	85.9%	70.6%	82.5%	80.1%	83.5%

## Isospin breaking effects in the $\pi ^0-\eta -{\eta ^\prime }$ system

Table 13 collects the main $\hbox {EBHLS}_2$ results related to isospin breaking effects in the $[\pi ^0,\eta ,{\eta ^\prime }]$ mixing. In contrast to Sect. 20, the parameter values returned by the different solutions may be very different and, then, averaging can often be misleading. On the other hand, to our knowledge, there are very few external evaluations of these parameters to compare with.

Regarding the $[m_{dd}^2- m_{uu}^2]$ evaluations, they are all much larger than the estimates based on meson masses we sketched above; whether this is due to higher-order corrections that are unaccounted for is unclear; in this case, one may expect the fit to take them effectively into account to accommodate the data. Related to this, fits performed using conditions $C^\prime$ ($\epsilon =\varpi _0$ and $\epsilon ^\prime =\varpi _0^\prime$) return the following piece of information:

109 $$\begin{aligned} \left\{ \begin{array}{lll} \displaystyle \mathrm{Solution} ~~ A_+:&{} \epsilon _0=[2.02 \pm 0.11]\times 10^{-2},~~ \\ &{}\displaystyle \mathrm{Prob.~~~} 74.0\% ,\\ \displaystyle \mathrm{Solution} ~~A_-: &{} \epsilon _0=[2.39 \pm 0.11]\times 10^{-2},~~\\ &{} \displaystyle \mathrm{Prob.~~~} 83.4\% , \end{array} \right. \end{aligned}$$

while the quark mass estimate expects $\epsilon _0 \simeq 1.2\times 10^{-2}$. Therefore, the picture looks somewhat confusing and may indicate that our evaluations for $[m_{dd}^2- m_{uu}^2]$ and $\epsilon _0$ absorb higher-order (or other kinds of) effects to accommodate the data.

The issue just raised obviously propagates to the evaluations for the $\eta$ and ${\eta ^\prime }$ fractions inside the physical $\pi ^0$. Here also, the values for $\epsilon$ and $\epsilon ^\prime$ are found to be much larger than expected. Related to this, Kroll [118] quoted an estimate for $\epsilon = [3.1 \pm 0.2]\times 10^{-2}$ coming from a ratio of^65^$\Psi (2S) \rightarrow J/\psi P$ decay widths, in line with our own findings.

Regarding $z_{Kroll}$, our $A_+$ and $A_-$ evaluations are consistent which each other up to the sign – which is the key feature of these solutions; its absolute magnitude is found in the $[6\div 8] \%$ range. Finally, $f_u$ and $f_d$ are found very close to $f_\pi$ when considering their uncertainty ranges.

Stated otherwise, the picture in the realm of isospin breaking effects involved in the $\pi ^0-\eta -{\eta ^\prime }$ system provided by phenomenology is somewhat confusing.

## Summary and conclusions

Three main topics have been addressed in the present paper: the treatment of $\tau$ dipion spectra, the update of the HVP-LO using global fit methods and the mixing properties of the $[\pi ^0,\eta ,{\eta ^\prime }]$ system showing up in the $\hbox {EBHLS}_2$ framework.

- Regarding the $\tau$ dipion spectra: In the previous version of the broken HLS model – named $\hbox {BHLS}_2$ [19] – the difficulty of the basic solution (BS) satisfactorily addressing the dipion spectrum collected by the Belle Collaboration [21] was noted; it was partly compensated by the primordial mixing (PM) of the vector fields, which led to the so-called reference solution (RS). However, the treatment of the Belle spectrum – which carries a statistics larger by a factor of $\simeq 50$ than Aleph [22] or Cleo [23] – deserves improvement. On the other hand, the analysis of the *lineshape* of the three $\tau$ dipion spectra clearly shows that there is no tension among them – as already noted in a previous study [41] – or with the other channels embodied inside our HLS framework, except for the spacelike spectra [54]. However, the present analysis clearly shows that the assumption which best fits the whole $\hbox {EBHLS}_2$ reference data set simultaneously – including the Belle spectrum – is slightly more involved than a mere rescaling. It is found that the kinetic breaking mechanism^66^ defined in Sect. 4 allows for a fair description of each of the Aleph, Belle and Cleo dipion spectra and, likewise, for the whole physics channels included inside the $\hbox {EBHLS}_2$ framework, in particular the pion form factor $F_\pi ^e(s)$ in the spacelike and timelike regions. On the other hand, the relevance of a kinetic breaking term – involving simultaneously components along the $T_0$, $T_3$ and $T_8$ basis matrices of the canonical Gell–Mann *U*(3) algebra – is also strengthened by considering properties related to the $[\pi ^0,\eta ,{\eta ^\prime }]$ system as it comes inside the $\hbox {EBHLS}_2$ framework. This led us to examine the consequences following from imposing conditions to matrix elements of the axial currents as expressed by Kroll [38]:
  $$\begin{aligned}<0|J_\mu ^q |[q^\prime \overline{q^\prime }](p)>=ip_\mu f_q \delta _{q q^\prime }, \{ [q {\overline{q}}], q=u,d,s\}\end{aligned}$$
  where the various $J_\mu ^q$ are the axial currents associated with the leading $[q {\overline{q}}]$ terms occurring in the Fock expansion of the $[\pi ^0,\eta _0,\eta _8]$ bare fields. Within the $\hbox {EBHLS}_2$ context, these conditions relate the mixing properties of the $[\pi ^0,\eta ,{\eta ^\prime }]$ system and the $\tau$ dipion spectrum because of the $\pi ^0$ meson. More precisely, it is proven in Sect. 16 that the solutions satisfying the Kroll conditions written just above generate nontrivial correlations between $F_\pi ^\tau (s=0)$ and the $\Lambda _1$ parameter traditionally included in EChPT to break the *U*(3) symmetry of the chiral Lagrangian [32, 33, 37] via a sole singlet term $\Lambda _1/2 \partial _\mu \eta _0\partial ^\mu \eta _0$. As a matter of fact, the Kroll conditions imposed on the $\hbox {EBHLS}_2$ Lagrangian relate the breaking of *U*(3) symmetry in the PS sector and the violation of CVC in the $\tau$ decay which explains the observed Belle spectrum; this CVC violation is invisible in the Aleph and Cleo spectra because of their lower statistics, but our fits illustrate that Aleph and Cleo absorb it quite naturally, as is obvious from Table 1. It is clear that this unexpected property deserves confirmation, and a forthcoming high statistics $\tau$ dipion spectrum is welcome to answer this question. On the other hand, the picture which emerges from $\hbox {EBHLS}_2$ indicates that using $\tau$ data to estimate the isospin breaking effects involved in $F_\pi ^e(s)$ is not straightforward outside a global fit context.

- The $\hbox {EBHLS}_2$ update of the muon HVP-LO raises several topics:
  1. Using the $\hbox {EBHLS}_2$ model, we examine the two recently published data samples of interest in the HLS energy range ($\le 1.05$ GeV). The BESIII $e^+ e^- \rightarrow \pi ^0\pi ^+ \pi ^-$ cross section [24] is important as it doubles the statistics covering this annihilation channel. Once the energies of this spectrum are appropriately^67^ recalibrated to match the energy scale of the ($> 50$) data samples already included in our standard sample set, it is shown that the $\hbox {EBHLS}_2$ framework leads to fairly good global fit properties. The SND Collaboration running on the new VEPP-2000 facility has produced a new spectrum [25] for the $e^+ e^- \rightarrow \pi ^+ \pi ^-$ cross section covering the HLS energy range which may allow us to readdress the KLOE–Babar controversy. Indeed, comparing the SND spectrum properties with those of the samples already belonging to our reference benchmark gives us the opportunity to re-emphasize our sample analysis method. Importantly, our approach is based on a few salient properties: (i) we stick to using in fits only the uncertainty information provided by each experiment and refrain from using any additional input such as error inflation factors; (ii) we treat canonically the normalization uncertainty [75]; (iii) preliminary fits allow us to identify the reference benchmark data samples by their satisfactory fit properties; the reference benchmark is found to include more than 90% of the available data samples covering all the channels addressed by $\hbox {EBHLS}_2$. Then, any newcomer sample is appended to the statistically consistent reference benchmark within a global fit: If its fit quality is satisfactory, it becomes part of the reference benchmark; otherwise, having detected inconsistencies between the newcomer and the reference benchmark samples, we discard the newcomer, in this way preserving the statistical consistency of the reference benchmark. The outcome can be summarized as follows: Naming $\mathcal{H}$ the set of all reference data samples except for the dipion spectra from KLOE and BaBar, it is shown that the most consistent combinations we can define are $\mathcal{H}_K =\{\mathcal{H} + \mathrm{KLOE}\}$ and $\mathcal{H}_B =\{\mathcal{H} + \mathrm{BaBar}\}$, the goodness of fit clearly favoring $\mathcal{H}_K$ compared to $\mathcal{H}_B$; moreover, the goodness of fit for each of $\mathcal{H}_K$ and $\mathcal{H}_B$ is much better than those for $\mathcal{H}_{KB} =\{\mathcal{H} + \mathrm{KLOE + BaBar} \}$. To deal with the muon HVP-LO issue, this observation has led us to perform separate analyses for $\mathcal{H}_K$ and $\mathcal{H}_B$ and avoid using $\mathcal{H}_{KB}$, which returns a poor probability and is found to produce significant biases compared to either $\mathcal{H}_K$ or $\mathcal{H}_B$. This is further discussed below. On the other hand, when a new data sample covering the $e^+ e^- \rightarrow \pi ^+ \pi ^-$ annihilation channel is published, the issue is always to re-examine whether it may or may not favor one of the $\mathcal{H}_K$ and $\mathcal{H}_B$ sample combinations. It has been found previously that the dipion spectra referred to above as NSK and Cleo-c do not substantially modify the fit picture of either of the $\mathcal{H}_K$ or $\mathcal{H}_B$ combinations; the BESIII sample – recently corrected [27] – is reported to rather favor $\mathcal{H}_K$ over $\mathcal{H}_B$, but nothing is really conclusive. The question is thus whether the SND spectrum [25] modifies the picture. The main results of our study are gathered^68^ in Fig. 7; the fit properties displayed therein indicate a better consistency with the $\mathcal{H}_K$ combination over the $\mathcal{H}_B$ one; however, there is still something unclear with the reported SND uncertainty information – or its dealing with – which, for now, leads us to use it only to estimate systematics. To conclude this topic, we should note that our reference set of data samples contains 1366 pieces of information. Besides the data samples covering the six annihilation channels already listed and the $\tau$ dipion spectra, one finds the partial widths for the $P \rightarrow \gamma \gamma$ decays and the $VP{\eta ^\prime }$ couplings not involved in the listed annihilation channels; the PDG value for the ratio of decay constants $f_K/f_\pi$ is also included. Stated otherwise, $\hbox {EBHLS}_2$ treats consistently the largest set of data and physics channels ever submitted to a unified description in the non-perturbative QCD region. Global fits have been performed under various conditions and return probabilities in the range of 80% to 90% for the $\mathcal{H}_K$ set combination and $\simeq 40\%$ for the $\mathcal{H}_B$ combination; the results based on $\mathcal{H}_K$ are discussed in Sect. 10, and their results gathered in Table 4, those based on the $\mathcal{H}_B$ combination, are the matter of Sect. 11.5.
  2. Regarding model dependence of the HLS estimates for the muon HVP-LO: In order to determine possible model dependence effects, the most appropriate approach is to compare the information derived from our fits with the corresponding information derived by others using so-called direct numerical integration methods – which are also far from being free of assumptions. Table 5 collects the numerical estimates for $a_\mu (\pi \pi )$ over the range $s \in [0.35,0.85]$ $\hbox {GeV}^2$ derived by the KLOE Collaboration itself [80] for the different data samples they published (KLOE08, KLOE10, KLOE12) and their combination (KLOE85). Including each of these samples as single representative of the $\pi \pi$ channel within the $\hbox {EBHLS}_2$ fitting procedure, one gets the numbers displayed in the second data column, with fit properties shown in the third data column. Except for KLOE08, which yields a poor goodness of fit, each “experimental” central value is distant from its $\hbox {EBHLS}_2$ analog by only a fraction of the relevant $\sigma _{exp}$; moreover, the gain in precision by performing global fitting is especially striking here, as the uncertainty of the fitted $a_\mu (\pi \pi )$ values is significantly smaller than their corresponding $\sigma _{exp}$’s. Comparing different methods of combining data is the subject of Table 6. This illustrates that, besides the selection of data samples, the way to deal with the reported normalization uncertainty is a much more significant source of bias than the choice of a model, even if ours, by correlating different channels with $\pi \pi$, allows for a much improved uncertainty for the $\pi \pi$ contribution – which is just the purpose for promoting global fit methods.
  3. Evaluations of the muon HVP: KLOE versus BaBar. The matter of Sect. 11.2 is to deal with various estimates for the HVP-LO derived from $\hbox {EBHLS}_2$ under various fit conditions. Table 7 displays specifically our results concerning the energy region up to 1.05 GeV and has to be completed with information given in Table 8 to derive the full HVP-LO. The content of Table 7 is associated with using for the fits what was named above in this section the $\{ \mathcal{H} + \mathrm{KLOE}\}$ sample set. Similarly, Sect. 11.5 provides the analog evaluation based on using the $\{ \mathcal{H} + \mathrm{BaBar} \}$ sample set. One gets for the muon HVP-LO:
    $$\begin{aligned} \left\{ \begin{array}{ll} \displaystyle \{ \mathcal{H} + \mathrm{KLOE}\} &{} \Longrightarrow a_\mu ^{HVP-LO} = 687.48 \pm 2.93_{fit} \\ &{} + \left[ ^{+2.31}_{-0.69} \right] _{syst}, ~~ 90 \% \mathrm{~~Prob.}\\ \displaystyle \{ \mathcal{H} + \mathrm{BaBar} \} &{} \Longrightarrow a_\mu ^{HVP-LO} = 692.53 \pm 2.95_{fit} \\ &{}+ \left[ ^{+2.31}_{-0.69} \right] _{syst}, ~~ 40 \% \mathrm{~~Prob.} \end{array} \right. \end{aligned}$$
    in units of $10^{-10}$. These are displayed together with other estimates in Fig. 9. One observes the strong effect of using $\{ \mathcal{H} + \mathrm{BaBar} \}$ preferably to $\{ \mathcal{H} + \mathrm{KLOE}\}$ despite the better goodness of fit of the latter set. We should note that the $\{ \mathcal{H} + \mathrm{BaBar} \}$ evaluation of the HVP-LO differs from the KNT19 evaluation [91] by only $0.42\times 10^{-10}$. However, taking into account the $5.47\times 10^{-10}$ difference between the BaBar and KLOE based evaluations, it may appear hazardous to perform any kind of combination of these. Nevertheless, it seems interesting to quote the results derived from a fit based solely on the $\mathcal{H}$ sample set; indeed, $\mathcal{H}$ only includes the NSK, Cleo-c and BESIII samples as representatives of the $\pi ^+ \pi ^-$ annihilation channel for which there is a commonly shared consensus. One thus gets
    $$\begin{aligned} \begin{array}{ll} \displaystyle \{ \mathcal{H} \}~~~ &{} \Longrightarrow a_\mu ^{HVP-LO} = 689.43 \pm 3.08_{fit} \\ &{}+ \left[ ^{+2.31}_{-0.69} \right] _{syst}, ~~ 91 \% \mathrm{~~Prob.} \end{array} \end{aligned}$$
    from a fit which also returns $\chi ^2/N_{pts}=1137/1231$. This evaluation, just midway between the $\{\mathcal{H} + \mathrm{KLOE}\}$ and $\{\mathcal{H} + \mathrm{BaBar}\}$ estimates, still benefits from a very good uncertainty and from a probability as good as those of the $\{\mathcal{H} + \mathrm{KLOE}\}$ fit. Compared to the average experimental value [4] for $a_\mu$ and taking into account the systematic uncertainties, we find for the difference $\Delta a_\mu =a_\mu ^{exp}-a_\mu ^{pheno.}$ a significance greater than $5.3 \sigma$ (KLOE) or $4.4 \sigma$ (BaBar). It is worth noting that the difference between these evaluations is *not* a model effect but a pure reflection of the tension between the BaBar and KLOE evaluations differing by $1.9\sigma _{fit}$ from each other. Regarding the hiatus between the LQCD evaluation [3] for the muon HVP-LO and any of the evaluations based on dispersive methods shown in Fig. 9, it looks uneasy yielding a missing $\delta a_\mu \simeq (10\div 20) \times 10^{-10}$ from annihilation data below $\simeq 1$ GeV.
- Regarding the $[\pi ^0,\eta ,{\eta ^\prime }]$ mixing properties: The $\hbox {EBHLS}_2$ Lagrangian provides a convenient framework for also examining the mixing properties of the $[\pi ^0,\eta ,{\eta ^\prime }]$ system. As this Lagrangian allows us to derive the various axial currents, it is possible to explicitly construct the parameterizations in the so-called octet-singlet and quark flavor bases. It is found that, at leading order in breakings, one recovers the known expressions – compare to [32, 37] for instance – somewhat generalized to also include the $\lambda _8$ and $\lambda _3$ terms. Related to this, it has been found worthwhile to examine in detail how the Kroll conditions noted at the beginning of this section can be fulfilled by the $\hbox {EBHLS}_2$ Lagrangian. It is found that two solutions – named $A_\pm$ – among the four possible ones lead to fair descriptions of our whole reference set of data. The $A_+$ and $A_-$ solutions return similar fit parameter values, and the $A_-$ solution is slightly favored compared^69^ to $A_+$. However, an unexpected aspect appears: the kinetic breaking term of the PS fields which is usually a determinant term leading to solely a PS singlet contribution $\partial _\mu \eta _0 \partial _\mu \eta _0$ cannot come alone and should be complemented by quadratic terms also involving the $\pi ^0$ and $\eta _8$ field derivatives. It thus follows that the Kroll conditions generate a violation of CVC in the dipion spectrum of the $\tau$ lepton decay, as already noted. One may expect that these conclusions are not specific to the broken HLS modelings. Using the fit results derived by running the $A_+$ and $A_-$ solutions to the Kroll conditions, the octet-singlet and quark flavor basis parameterization of the $[\pi ^0,\eta ,{\eta ^\prime }]$ mixing are computed (see Table 11) and compared with other available estimates (see Table 12). A good agreement is observed with the other estimates, although here also with better precision for the $\hbox {EBHLS}_2$ evaluations. The isospin breaking effects which can affect the $[\pi ^0,\eta ,{\eta ^\prime }]$ system [38] are also derived (see Table 13), but here there is little external information with which to make comparisons and form conclusions.

## Data Availability

This manuscript has no associated data or the data will not be deposited. [Authors’ comment: All original data we can provide are already shown in the text, Tables and Figures.]

## Appendix A: The *AAP* and *VVP* anomalous sectors

### A.1 The *AAP* Lagrangian

The *AAP* Lagrangian is given by:

110 $$\begin{aligned} \displaystyle \mathcal{L}_{AAP}= -\frac{3 \alpha _{em}}{\pi f_\pi }(1-c_4) ~\epsilon ^{\mu \nu \alpha \beta } \partial _\mu A_\nu \partial _\alpha A_\beta \mathrm {Tr} \left[ Q^2 P \right] , \end{aligned}$$

where *Q* is the quark charge matrix and *P* denotes the *U*(3) symmetric matrix of the bare pseudoscalar fields. Let us define:

111 $$\begin{aligned} \begin{array}{lll} \displaystyle \lambda _0^\prime &{}= \left[ \lambda _3 + \lambda _0 \sqrt{\frac{2}{3}} ~\frac{5 z_A^2+1}{3 z_A^2} \right] ,\\ \displaystyle \lambda _8^\prime &{}= \left[ \lambda _3 + \lambda _8 \frac{1}{\sqrt{3}} \frac{5 z_A^2-2}{3 z_A^2}\right] \end{array} \end{aligned}$$

and the angle $\delta _P$:

112 $$\begin{aligned} \left\{ \begin{array}{ll} \sin {\theta _P}= \displaystyle \frac{1}{\sqrt{3}} \left( \cos {\delta _P} + \sqrt{2} \sin {\delta _P} \right) \\ \cos {\theta _P}= \displaystyle \frac{1}{\sqrt{3}} \left( \sqrt{2} \cos {\delta _P} - \sin {\delta _P} \right) \end{array} \right. \end{aligned}$$

which measures the departure from ideal mixing ($\theta _I=\arctan {1/\sqrt{2}}\simeq 35^\circ$): $\delta _P =\theta _P - \theta _I$. Defining:

113 $$\begin{aligned} \left\{ \begin{array}{ll} \displaystyle g_{\pi ^0\gamma \gamma }=&{}\displaystyle ~~\frac{1}{6} \left\{ 1 - \frac{5}{6}\Delta _A + \frac{\lambda _3}{2} \left[ \lambda _3-\lambda _0^\prime -\lambda _8^\prime \right] \right\} \\ ~&{} +\displaystyle \frac{\epsilon }{6\sqrt{3}} \left\{ \frac{5z_A-2}{3z_A}\cos {\theta _P}-\sqrt{2}\frac{5z_A+1}{3z_A}\sin {\theta _P} \right\} \\ ~ &{} \displaystyle + \frac{\epsilon ^\prime }{6\sqrt{3}}\left\{ \frac{5z_A-2}{3z_A}\sin {\theta _P}+ \sqrt{2}\frac{5z_A+1}{3z_A}\cos {\theta _P}\right\} ,\\ \displaystyle g_{\eta \gamma \gamma }=&{}\displaystyle -\frac{\epsilon }{6} +\frac{\cos {\delta _P}}{36 z_A} \left\{ -2\sqrt{2} + \sqrt{3} \lambda _0 \lambda _0^\prime \right. \\ &{} \left. -\sqrt{6} \lambda _8 \lambda _8^\prime - \frac{5 z_A^2+4}{3 z_A^2} \lambda _0 \lambda _8 \right\} \\ ~&{}+\displaystyle \frac{\sin {\delta _P}}{36} \left\{ 3 \Delta _A-10 + \sqrt{6}\lambda _0 \lambda _0^\prime \right. \\ &{}\left. + \sqrt{3}\lambda _8 \lambda _8^\prime +\sqrt{2} \frac{10 z_A^2 -1}{3 z_A^2} \lambda _0 \lambda _8 \right\} , \\ \displaystyle g_{{\eta ^\prime }\gamma \gamma }=&{}\displaystyle -\frac{\epsilon ^\prime }{6} \displaystyle -\frac{\cos {\delta _P}}{36} \left\{ 3 \Delta _A-10 + \sqrt{6}\lambda _0 \lambda _0^\prime \right. \\ &{} \left. + \sqrt{3}\lambda _8 \lambda _8^\prime +\sqrt{2} \frac{10 z_A^2 -1}{3 z_A^2} \lambda _0 \lambda _8 \right\} \\ ~&{}\displaystyle + \frac{\sin {\delta _P}}{36 z_A} \left\{ -2\sqrt{2} + \sqrt{3} \lambda _0 \lambda _0^\prime -\sqrt{6} \lambda _8 \lambda _8^\prime \right. \\ &{} \left. - \frac{5 z_A^2+4}{3 z_A^2} \lambda _0 \lambda _8\right\} , \end{array} \right. \end{aligned}$$

the coupling constants of the physical pseudoscalar fields to a photon pair, $\pi ^0 \gamma \gamma$, $\eta \gamma \gamma$ and $\eta ^\prime \gamma \gamma$, are given by:

114 $$\begin{aligned} \displaystyle G_{P^0 \gamma \gamma } = -\frac{3 \alpha _{em}}{\pi f_\pi } (1-c_4) g_{P^0 \gamma \gamma } . \end{aligned}$$

### A.2 The *VVP* Lagrangian

The *VVP* Lagrangian is given by:

115 $$\begin{aligned} \displaystyle \mathcal{L}_{VVP}= & {} -\frac{3 g^2}{4 \pi ^2 f_\pi }~c_3 ~\epsilon ^{\mu \nu \alpha \beta } \mathrm {Tr} \left[ \partial _\mu V_\nu \partial _\alpha V_\beta P \right] , \nonumber \\ ~~~~ \displaystyle C= & {} -\frac{N_c g^2 c_3}{4 \pi ^2 f_\pi }. \end{aligned}$$

#### A.2.1 The $VV\pi$ Lagrangians

The $VV\pi$ Lagrangians relevant for our phenomenology are given by:

116 $$\begin{aligned} \displaystyle \mathcal{L}_{VVP}(\pi ^\pm )= & {} \displaystyle \frac{C}{2} \epsilon ^{\mu \nu \alpha \beta } \Biggl \{ \left[ \left( 1+\frac{2 \xi _0+\xi _8}{3} \right) \right. \partial _\mu \omega _\nu ^I \nonumber \\&\left. +\frac{\sqrt{2}}{3} (\xi _0-\xi _8) \partial _\mu \phi ^I_\nu \right] \nonumber \\&\times \left[ \partial _\alpha \rho ^+_\beta \pi ^- +\partial _\alpha \rho ^-_\beta \pi ^+ \right] \Biggr \} \end{aligned}$$

and:

117 $$\begin{aligned} \mathcal{L}_{VVP}(\pi ^0)= & {} \displaystyle \frac{C}{2} \epsilon ^{\mu \nu \alpha \beta } \Biggl \{ G_0 \partial _\mu \rho ^I_\nu \partial _\alpha \omega ^I_\beta +G_1\nonumber \\&\left[ 2 \partial _\mu \rho ^-_\nu \partial _\alpha \rho ^+_\beta + \partial _\mu \rho ^I_\nu \partial _\alpha \rho ^I_\beta + \partial _\mu \omega ^I_\nu \partial _\alpha \omega ^I_\beta \right] \Biggr .\nonumber \\ \displaystyle &+ G_2 \partial _\mu \Phi ^I_\nu \partial _\alpha \Phi ^I_\beta + G_3 \partial _\mu \rho ^I_\nu \partial _\alpha \Phi ^I_\beta \Biggr \}~\pi ^0 \end{aligned}$$

where:

118 $$\begin{aligned} \left\{ \begin{array}{ll} \displaystyle G_0=&{} \left[ 1-\frac{\lambda _3^2}{2} + \frac{2 \xi _0+\xi _8}{3} +\xi _3 \right] ,\\ \displaystyle G_1 =&{} -\frac{1}{4\sqrt{3}} \left[ \sqrt{3} \Delta _A +\lambda _3 (\sqrt{2} \lambda _0 + \lambda _8) \right] \\ &{} +\frac{1}{2} \left[ \epsilon ^\prime \cos {\delta _P} - \epsilon \sin {\delta _P} \right] , \\ \displaystyle G_2 =&{} -\frac{\lambda _3}{2 z_A^2\sqrt{6}} \left[ \lambda _0 -\sqrt{2} \lambda _8\right] \\ &{}-\frac{1}{z_A \sqrt{2}} \left[ \epsilon ^\prime \sin {\delta _P} + \epsilon \cos {\delta _P} \right] , \\ \displaystyle G_3 =&{} \frac{\sqrt{2}}{3} (\xi _0-\xi _8). \end{array} \right. \end{aligned}$$

As actually, one imposes $\xi _0=\xi _8$, one has $G_3 =0$.

#### A.2.2 The $VV\eta$ Lagrangian

The $VV\eta$ Lagrangian is given by:

119 $$\begin{aligned} \begin{array}{ll} \displaystyle \mathcal{L}_{VVP}(\eta )=&{} \displaystyle \frac{C}{2} \epsilon ^{\mu \nu \alpha \beta } \Biggl \{ K_1 \partial _\mu \rho ^-_\nu \partial _\alpha \rho ^+_\beta + K_2 \partial _\mu \rho ^I_\nu \partial _\alpha \rho ^I_\beta \\ &{}+ K_3 \partial _\mu \omega ^I_\nu \partial _\alpha \omega ^I_\beta + K_4 \partial _\mu \Phi ^I_\nu \partial _\alpha \Phi ^I_\beta \Biggr .\\ &{} \displaystyle \Biggl . + K_5 \partial _\mu \omega ^I_\nu \partial _\alpha \Phi ^I_\beta + K_6 \partial _\mu \rho ^I_\nu \partial _\alpha \omega ^I_\beta \Biggr \}~\eta . \end{array} \end{aligned}$$

Defining:

120 $$\begin{aligned} \left\{ \begin{array}{ll} \displaystyle H_1= \frac{1}{12 z_A} ~\kappa _0 \kappa _8 ,\\ \displaystyle H_2 = \frac{1}{12} \left[ \kappa _0^2 -6 \right] , \\ \displaystyle H_3 = \frac{\sqrt{2}}{12 z_A^3} \left[ \kappa _8^2 -2 z_A^2 (3+2 \xi _0+4 \xi _8) \right] , \end{array} \right. \end{aligned}$$

where:

121 $$\begin{aligned} \displaystyle \kappa _0= \sqrt{2} \lambda _0 + \lambda _8 ~~~~ \mathrm{and}~~~~ \kappa _8= \lambda _0 - \sqrt{2} \lambda _8, \end{aligned}$$

the $VV\eta$ couplings are:

122 $$\begin{aligned} \left\{ \begin{array}{ll} \displaystyle K_1= 2 \left[ H_1 \cos {\delta _P} + H_2 \sin {\delta _P} \right] ,\\ \displaystyle K_2 = H_1 \cos {\delta _P} + (H_2 - \xi _3) \sin {\delta _P} , \\ \displaystyle K_3 = H_1 \cos {\delta _P} + \left[ H_2 - \frac{2 \xi _0+\xi _8}{3} \right] \sin {\delta _P} , \\ \displaystyle K_4 = H_3 \cos {\delta _P} + \frac{\sqrt{2}}{z_A} H_1 \sin {\delta _P} , \\ \displaystyle K_5 = -\frac{(\xi _0 -\xi _8)}{3 z_A} \left[ 2 \cos {\delta _P} + z_A \sqrt{2} \sin {\delta _P} \right] , \\ \displaystyle K_6 = \frac{\lambda _3 \kappa _8}{2 z_A\sqrt{3}} \cos {\delta _P} +\frac{1}{2 \sqrt{3}} \left[ \sqrt{3} \Delta _A + \lambda _3 \kappa _0 \right] \sin {\delta _P} - \epsilon . \end{array} \right. \end{aligned}$$

#### A.2.3 The $VV{\eta ^\prime }$ Lagrangian

The $VV{\eta ^\prime }$ Lagrangian is given by:

123 $$\begin{aligned} \begin{array}{ll} \displaystyle \mathcal{L}_{VVP}({\eta ^\prime })=&{} \displaystyle \frac{C}{2} \epsilon ^{\mu \nu \alpha \beta } \Biggl \{ K^\prime _1 \partial _\mu \rho ^-_\nu \partial _\alpha \rho ^+_\beta + K^\prime _2 \partial _\mu \rho ^I_\nu \partial _\alpha \rho ^I_\beta \\ &{}+ K^\prime _3 \partial _\mu \omega ^I_\nu \partial _\alpha \omega ^I_\beta + K^\prime _4 \partial _\mu \Phi ^I_\nu \partial _\alpha \Phi ^I_\beta \Biggr . \\ &{} \displaystyle \Biggl . + K^\prime _5 \partial _\mu \omega ^I_\nu \partial _\alpha \Phi ^I_\beta + K^\prime _6 \partial _\mu \rho ^I_\nu \partial _\alpha \omega ^I_\beta \Biggr \}~{\eta ^\prime }, \end{array} \end{aligned}$$

the $VV{\eta ^\prime }$ couplings are:

124 $$\begin{aligned} \left\{ \begin{array}{ll} \displaystyle K^\prime _1= 2 \left[ - H_2 \cos {\delta _P} + H_1 \sin {\delta _P} \right] ,\\ \displaystyle K^\prime _2 = -(H_2 - \xi _3)\cos {\delta _P} + H_1 \sin {\delta _P} ,\\ \displaystyle K^\prime _3 = -\left[ H_2 - \frac{2 \xi _0+\xi _8}{3} \right] \cos {\delta _P} + H_1 \sin {\delta _P} , \\ \displaystyle K^\prime _4 = H_3 \sin {\delta _P} - \frac{\sqrt{2}}{z_A} H_1 \cos {\delta _P} , \\ \displaystyle K^\prime _5 = -\frac{(\xi _0 -\xi _8)}{3 z_A} \left[ - z_A \sqrt{2} \cos {\delta _P} +2 \sin {\delta _P} \right] ,\\ \displaystyle K^\prime _6 = -\frac{1}{2 \sqrt{3}} \left[ \sqrt{3} \Delta _A + \lambda _3 \kappa _0 \right] \cos {\delta _P} + \frac{\lambda _3 \kappa _8}{2 z_A\sqrt{3}} \sin {\delta _P} -\epsilon ^\prime . \end{array} \right. \end{aligned}$$

## Appendix B: The *APPP* and *VPPP* anomalous sectors

### B.1 The APPP Lagrangian

The *APPP* Lagrangian is given by:

125 $$\begin{aligned}&\displaystyle \mathcal{L}_{APPP}\nonumber \\&\quad = D ~\epsilon ^{\mu \nu \alpha \beta } A_\mu \mathrm {Tr} \left[ Q \partial _\nu P \partial _\alpha P \partial _\beta P\right] , ~~ \nonumber \\&\displaystyle D = -i\frac{N_c e}{3 \pi ^2 f_\pi ^3} \left[ 1-\frac{3}{4}(c_1-c_2+c_4)\right] . \end{aligned}$$

Limiting oneself to the Lagrangian pieces relevant for our purpose, it can be written:

126 $$\begin{aligned} \displaystyle \mathcal{L}_{APPP}= & {} D \epsilon ^{\mu \nu \alpha \beta } A_\mu \left\{ g_{\gamma \pi ^0} \partial _\nu \pi ^0 + g_{\gamma \eta } \partial _\nu \eta + g_{\gamma {\eta ^\prime }} \partial _\nu {\eta ^\prime }\right\} \nonumber \\&\quad \partial _\alpha \pi ^- \partial _\beta \pi ^+ , \end{aligned}$$

in terms of fully renormalized PS fields. Defining:

127 $$\begin{aligned} \displaystyle \kappa _0= \sqrt{2} \lambda _0 + \lambda _8 ~~~~ \mathrm{and}~~~~ \kappa _8= \lambda _0 - \sqrt{2} \lambda _8, \end{aligned}$$

the couplings can be written:

128 $$\begin{aligned} \left\{ \begin{array}{ll} \displaystyle g_{\gamma \pi ^0} &{}= -\frac{1}{4} \left[ 1 - \frac{\Delta _A}{2} -\frac{\lambda _3}{2\sqrt{3}} \left( \kappa _0 +\sqrt{3}\lambda _3 \right) \right. \\ &{}\left. -\epsilon \sin {\delta _P} +\epsilon ^\prime \cos {\delta _P} \right] ,\\ \displaystyle g_{\gamma \eta } &{}= -\frac{\kappa _0 + \sqrt{3} \lambda _3}{24 z_A} \left[ \kappa _8 \cos {\delta _P} + z_A \kappa _0 \sin {\delta _P} \right] \\ &{}+\frac{\sin {\delta _P}}{4} \left( 1 -\frac{\Delta _A}{2} \right) + \frac{\epsilon }{4},\\ \displaystyle g_{\gamma {\eta ^\prime }} &{}= ~~ \frac{\kappa _0 + \sqrt{3} \lambda _3}{24 z_A} \left[ z_A \kappa _0 \cos {\delta _P} - \kappa _8 \sin {\delta _P} \right] \\ &{}- \frac{\cos {\delta _P}}{4} \left( 1 -\frac{\Delta _A}{2} \right) + \frac{\epsilon ^\prime }{4}. \end{array} \right. \end{aligned}$$

### B.2 The VPPP Lagrangian

The *VPPP* Lagrangian is given by:

129 $$\begin{aligned} \displaystyle \mathcal{L}_{VPPP}= & {} E ~\epsilon ^{\mu \nu \alpha \beta } \mathrm {Tr} \left[ V_\mu \partial _\nu P \partial _\alpha P \partial _\beta P\right] , \nonumber \\ \displaystyle E= & {} -i\frac{N_c g}{4 \pi ^2 f_\pi ^3} \left[ c_1-c_2-c_3\right] . \end{aligned}$$

Its relevant part can be rewritten:

130 $$\begin{aligned} \displaystyle \mathcal{L}_{VPPP}= & {} E ~\epsilon ^{\mu \nu \alpha \beta } \sum _{V = (\rho ^I,\omega ^I,\phi ^I)} V_\mu \nonumber \\&\quad \times \left\{ g_{V \pi ^0} \partial _\nu \pi ^0 + g_{V \eta } \partial _\nu \eta + g_{V {\eta ^\prime }} \partial _\nu {\eta ^\prime }\right\} \nonumber \\&\quad \partial _\alpha \pi ^- \partial _\beta \pi ^+ , \end{aligned}$$

in terms of ideal vector fields and fully renormalized PS fields. The corresponding couplings are:

131 $$\begin{aligned} \left\{ \begin{array}{ll} \displaystyle g_{\rho ^I \pi ^0} &{}= \frac{1}{4} \left[ \frac{\Delta _A}{2} + \frac{1}{2\sqrt{3}} \lambda _3 \kappa _0 \right. \\ &{}\quad \left. +\epsilon \sin {\delta _P}-\epsilon ^\prime \cos {\delta _P} \right] ,\\ \displaystyle g_{\rho ^I \eta } &{}= -\frac{\kappa _0}{24 z_A} \left[ \kappa _8 \cos {\delta _P} + z_A \kappa _0\sin {\delta _P} \right] \\ &{}\quad +\frac{\sin {\delta _P}}{4} \left( 1+\xi _3 \right) ,\\ \displaystyle g_{\rho ^I {\eta ^\prime }} &{}= \frac{\kappa _0}{24 z_A} \left[ z_A \kappa _0 \cos {\delta _P} - \kappa _8 \sin {\delta _P} \right] \\ &{}\quad - \frac{\cos {\delta _P}}{4} \left( 1+\xi _3 \right) , \end{array} \right. \end{aligned}$$

and:

132 $$\begin{aligned} \left\{ \begin{array}{ll} \displaystyle g_{\omega ^I \pi ^0} &{}= -\frac{3}{4} \left[ 1 - \frac{\lambda _3^2}{2} + \frac{2 \xi _0 +\xi _8}{3} \right] ,\\ \displaystyle g_{\omega ^I \eta } &{}= -\frac{\lambda _3 \sqrt{3}}{8 z_A} \left[ \kappa _8 \cos {\delta _P} + z_A \kappa _0\sin {\delta _P} \right] \\ &{}\quad - \frac{3 \Delta _A}{8} \sin {\delta _P} + \frac{3 }{4} \epsilon ,\\ \displaystyle g_{\omega ^I {\eta ^\prime }} &{}= \frac{\lambda _3 \sqrt{3}}{8 z_A} \left[ z_A \kappa _0 \cos {\delta _P} - \kappa _8 \sin {\delta _P} \right] \\ &{}\quad + \frac{3 \Delta _A}{8} \cos {\delta _P}+ \frac{3 }{4} \epsilon ^\prime . \end{array} \right. \end{aligned}$$

Finally, we also have:

133 $$\begin{aligned} \begin{array}{lll} \displaystyle g_{\phi ^I \pi ^0} = -\frac{\sqrt{2}}{4} \left( \xi _0-\xi _8 \right) ,&\displaystyle g_{\phi ^I \eta } = 0,&\displaystyle g_{\phi ^I {\eta ^\prime }} = 0. \end{array} \end{aligned}$$

It should be noted that the condition $F_\pi ^e(0)=1+\mathcal{O}(\delta ^2)$ leads to $\xi _0=\xi _8$.

## Appendix C: The $e^+e^- \rightarrow \pi ^0\pi ^+\pi ^-$ cross section

The amplitude for the $\gamma ^* \rightarrow \pi ^0\pi ^+\pi ^-$ transition involves most of the FKTUY Lagrangian pieces; it can be written:

134 $$\begin{aligned} \displaystyle T(\gamma ^* \rightarrow \pi ^0\pi ^+\pi ^-)=T_{APPP}+T_{VPPP}+T_{VVP}, \end{aligned}$$

labeling each term by the particular piece of the FKTUY Lagrangian from which it originates. As already noted, because $c_3=c_4$ is assumed, there is no $T_{AVP}$ piece.

The $T_{APPP}$ contribution to the full $T(\gamma ^* \rightarrow \pi ^0\pi ^+\pi ^-)$ is:

135 $$\begin{aligned} \displaystyle T_{APPP}= & {} C_{APPP} \left[ 1 -G(\delta _P) \right] \epsilon _{\mu \nu \alpha \beta } ~~\epsilon _\mu (\gamma ) p_0^\nu , p_-^\alpha p_+^\beta ,\nonumber \\ \displaystyle C_{APPP}= & {} -\frac{ie}{4 \pi ^2 f_\pi ^3}\left[ 1-\frac{3}{4} (c_1-c_2+c_4) \right] , \end{aligned}$$

where $\epsilon _\mu (\gamma )$ is the off-shell photon polarisation vector, the other notations being obvious. One has also defined:

136 $$\begin{aligned} G(\delta _P)= & {} \left[ \frac{\Delta _A}{2}+ \epsilon \sin {\delta _P} -\epsilon ^\prime \cos {\delta _P} \right] \nonumber \\&+ \frac{\lambda _3}{2\sqrt{3}} (\sqrt{3}\lambda _3 +\sqrt{2}\lambda _0+\lambda _8) \;. \end{aligned}$$

Three pieces come from the *VPPP*:

137 $$\begin{aligned} T_{VPPP}= & {} \displaystyle C_{VPPP} \left[ \sum _{V=\rho ,\omega ,\phi } \frac{F^e_{V\gamma }(s)}{D_V(s)} g_{V\pi }^R (s) \right] \epsilon _{\mu \nu \alpha \beta } \nonumber \\&\times \epsilon _\mu (\gamma ) p_0^\nu p_-^\alpha p_+^\beta , \end{aligned}$$

where the renormalized vector couplings $g_{V\pi }^R (s)$ to three pions have been derived using the vector relation:

138 $$\begin{aligned} g_{V \pi }^R (s) =\mathcal{R }(s) g_{V \pi }^I, \end{aligned}$$

$\mathcal{R }(s)$ being the matrix given in Eq. (38) or in Eq. (43) of the $\hbox {BHLS}_2$ companion paper [19] depending on whether the primordial breaking is discarded or not. The components of the $g_{V \pi }^I$ vector which refer to the coupling of the ideal vector field combinations are:

139 $$\begin{aligned} \left\{ \begin{array}{ll} \displaystyle g_{\rho \pi }^I&{}=\frac{1}{4} \left[ \frac{\Delta _A}{2}+ \epsilon \sin {\delta _P} -\epsilon ^\prime \cos {\delta _P}\right. \\ &{}\quad \left. +\frac{\lambda _3}{2\sqrt{3}} (\sqrt{2}\lambda _0+\lambda _8) \right] ,\\ \displaystyle g_{\omega \pi }^I&{}=-\frac{3}{4} \left[ 1 +\frac{2\xi _0+\xi _8}{3} -\frac{\lambda _3^2}{2} \right] ,\\ \displaystyle g_{\phi \pi }^I&{}=-\frac{\sqrt{2}}{4} (\xi _0-\xi _8) . \end{array} \right. \end{aligned}$$

The $V-\gamma$ amplitudes $F^e_{V\gamma }(s)$ and the inverse $\rho$ propagators have been constructed in Sect. 11 of [19]. The inverse propagators for the $\omega$ and $\phi$ mesons have been discussed and defined in Sect. 9 of the same reference. We have also defined:

140 $$\begin{aligned} \displaystyle C_{VPPP}= -\frac{3ige}{4 \pi ^2 f_\pi ^3} \left[ c_1-c_2-c_3 \right] \;. \end{aligned}$$

The *VVP* Lagrangian piece in Eq. (117) given in terms of ideal vector fields has to be re-expressed in terms of their renormalized partners as developed in Sect. 12 of [19] – see Eqs. (70-75) therein. The simplest way to write $T(\gamma ^* \rightarrow \pi ^0 \pi ^+ \pi ^-)$ in a way easy to code within our global fit procedure is displayed just below.

We first define the $H_i(s)$ functions:

141 $$\begin{aligned} \left\{ \begin{array}{llll} \displaystyle H_0(s) =1 ,\\ \displaystyle H_1(s) = \frac{1}{D_\rho (s_{+-})} + \frac{1}{D_\rho (s_{0+})} + \frac{1}{D_\rho (s_{0-})},~\\ \displaystyle H_2(s)=\displaystyle \frac{1}{D_\rho (s_{+-})},\\ \displaystyle H_3(s)=\displaystyle {\widetilde{\alpha }} (s_{+-}) \left[ \frac{1}{D_\rho (s_{+-})} - \frac{1}{D_\omega (s_{+-})} \right] , \end{array} \right. \end{aligned}$$

where *s* is the incoming squared energy and the $s_{ij}$’s indicate the invariant mass squared of the corresponding outgoing (*i*, *j*) pairs; the tilde mixing angles are those defined by Equation (43) in [19]. $T_{VVP}$ depends on the three functions $(H_i(s), ~i=1~\cdots 3)$ with the *s*-dependent coefficients $F_i(s)$ given below.

Collecting all terms, the full amplitude is written as:

142 $$\begin{aligned}&T(\gamma ^* \rightarrow \pi ^0 \pi ^+ \pi ^-) \nonumber \\&\quad = \left[ F_0(s) H_0(s)+ C_{VVP}\sum _{i=1\cdots 3} F_i(s) H_i(s) \right] \nonumber \\&\qquad \times \epsilon _{\mu \nu \alpha \beta } ~~\epsilon _\mu (\gamma ) p_0^\nu p_-^\alpha p_+^\beta , \end{aligned}$$

with:

143 $$\begin{aligned} \displaystyle C_{VVP}= -i \frac{3 e g m^2 }{8 \pi ^2 f_\pi ^3} (1+\Sigma _V) c_3\;. \end{aligned}$$

In this way, to write the full amplitude, the various $F_i(s)$ functions only depend on the incoming off-shell photon energy squared *s*; the dependence upon the various sub-energies $s_{ij}$ is, instead, only carried by the $H_i(s)$ functions as clear from Eq. (141). One has:

144 $$\begin{aligned} \left\{ \begin{array}{llll} \displaystyle F_0(s) &{}= C_{APPP} \left[ 1-G(\delta _P)\right] \\ &{}\quad +C_{VPPP}\left[ \frac{F^R_{\rho \gamma }(s)}{D_\rho (s)} g_{\rho \pi }^R (s) +\frac{F^R_{\omega \gamma }(s)}{D_\omega (s)} g_{\omega \pi }^R (s)+ \frac{F^R_{\phi \gamma }(s)}{D_\phi (s)} g_{\phi \pi }^R (s) \right] ,\\ \displaystyle F_1(s) &{}= \displaystyle {\widetilde{\alpha }}(s)\frac{F^R_{\rho \gamma }(s)}{D_\rho (s)} + \left[ 1-\frac{\lambda _3 ^2}{2} \right] \left[ 1+\frac{2\xi _0+\xi _8}{3} \right] \\ &{}\quad \times \frac{F^R_{\omega \gamma }(s)}{D_\omega (s)} +\left[ \frac{\sqrt{2}}{3} (\xi _0-\xi _8) +{\widetilde{\gamma }}(s)\right] \frac{F^R_{\phi \gamma }(s)}{D_\phi (s)} ,\\ \displaystyle F_2(s) &{}= \displaystyle \left[ \epsilon ^\prime \cos {\delta _P}- \epsilon \sin {\delta _P} - \frac{\Delta _A}{2}\right. \\ &{}\quad \left. -\frac{\lambda _3}{2\sqrt{3}} (\sqrt{2} \lambda _0+\lambda _8) \right] \frac{F^R_{\rho \gamma }(s)}{D_\rho (s)} + 2 \xi _3 \frac{F^R_{\omega \gamma }(s)}{D_\omega (s)} , \\ \displaystyle F_3(s) &{}= \displaystyle \frac{F^R_{\rho \gamma }(s)}{D_\rho (s)} , \end{array} \right. \end{aligned}$$

where ${\widetilde{\alpha }}(s)=\alpha (s)+\psi _\omega$ and ${\widetilde{\gamma }}(s)=\gamma (s)+\psi _0$, to possibly keep track of the primordial breaking [19], $\alpha (s)$ and $\beta (s)$ being the angles generated by the dynamic mixing mechanism [14, 19].

A global fit to all cross sections but $e^+e^- \rightarrow \pi ^0 \pi ^+\pi ^-$ allows us to obtain the relevant parameters with a good approximation; then, having at hand all ingredients defining the $F_i(s)$’s, a first minimization run [75] including the $e^+e^- \rightarrow \pi ^0 \pi ^+\pi ^-$ cross section can be performed to also derive a first estimate for $c_1-c_2$. The output of this minuit minimization run is then used as input for a next minimization step. This initiates an iteration procedure involving all cross sections which is carried on up to convergence – when some criterion is met, e.g. generally $\Delta \chi ^2 \le 0.3$ – for the global $\chi ^2$ of all the processes involved in the $\hbox {EBHLS}_2$ procedure.

This method converges in a couple of minimization steps [75]. What makes such a minimization procedure unavoidable is that the $e^+e^- \rightarrow \pi ^0 \pi ^+\pi ^-$ cross section expression implies integrating over the $s_{ij}$’s at each step. This is obviously prohibitively computer time-consuming for a negligible gain. Hence, at each step, one starts by tabulating coefficient functions, exhibited in the next expression between curly brackets:

145 $$\begin{aligned}&\sigma (e^+e^- \rightarrow \pi ^0 \pi ^+\pi ^-,s) = \displaystyle \frac{\alpha _{\mathrm{em}} ~s^2}{192 \pi ^2} \displaystyle \nonumber \\&\quad \times \left[ \left\{ \int G \mathrm{d}x \mathrm{d}y \right\} |F_0(s)|^2 \right. \nonumber \\&\quad \left. + C_{VVP}^2 \sum _{i,j=1\cdots 3} F_i(s) F_j^*(s) \left\{ \int G H_i H_j^* \mathrm{d}x \mathrm{d}y \right\} \right. \nonumber \\&\quad \displaystyle \left. +C_{VVP} \sum _{i=1\cdots 3} \left( F_0(s) F_i^*(s) \left\{ \int G H_i^* \mathrm{d}x \mathrm{d}y\right\} \displaystyle \right. \right. \nonumber \\&\quad \left. \left. + F_0^*(s) F_i(s) \left\{ \int G H_i \mathrm{d}x \mathrm{d}y\right\} \right) \right] \end{aligned}$$

and these tables are used all along the next step. Equation (145) uses the Kuraev–Silagadze parameterization [119] and its kernel *G*(*x*, *y*) function; these are noted in Appendix H of [14].

## Appendix D: Energy shift of resonances and secondary ISR photons

For the BESIII data sample [24] of concern here, photon radiation effects have been unfolded by utilizing the PHOKHARA code [120], which also includes second-order photon emission.^70^ In the leading ISR process, $\sqrt{s}=3.773$ GeV is the energy at which the primary ISR photon is emitted. Here we address the emission of a second photon near the $\phi$ and $\omega$ resonances, where the corresponding radiation effect appears resonance-enhanced.

The effect of a photon radiated off a Breit–Wigner (BW) resonance is well known from the *Z* resonance physics at LEP I [123–126]. A specific analysis for the process $e^+e^- \rightarrow \pi ^+\pi ^-$ may also be found in [127, 128]. The leading effect is due to initial state radiation, where the observed cross section is given by a convolution^71^:

146 $$\begin{aligned} \sigma ^{\mathrm{obs}}(s)=\int _0^{1} dk \rho _{ini}(k)\;\sigma _{phys}(s(1-k)), \end{aligned}$$

where $k=E_\gamma /E_{e}$ is the energy emitted by the photon in units of the electron or positron energy $E_{e}$ at which the resonance is formed, and $s=4E_e^2$. The photon spectral function is

147 $$\begin{aligned} \rho _{ini}(k)=\beta k^{\beta -1}\,(1+ \delta _1^{v+s}+ \cdots ) +\delta _1^h + \cdots , \end{aligned}$$

with $\beta =\frac{2\alpha }{\pi }\,(L-1)$, $L=\ln \frac{s}{m_e^2}$ and the photon radiation corrections for the virtual + soft (v+s) and the hard (h) parts read:

148 $$\begin{aligned} \delta _1^{v+s}=\frac{\alpha }{\pi } \left( \frac{3}{2} \,L+\frac{\pi ^2}{3}-2 \right) \;; \delta _1^h = \frac{\alpha }{\pi }\,(1-L)(2-k). \end{aligned}$$

**Table 14:** The energy shifts in keV of the resonance locations by local ISR photon emission at $s=M_R^2$. We adopted PDG resonance parameter values (in MeV). The values for $(\delta E)^\mathrm{fit}$ are obtained in a global fit involving all 3$\pi$ data samples (BESIII+NSK)

	$M_R$	$\Gamma _R$	$\delta E$	$(\delta E)^{\mathrm{fit}}$
$\omega$	782.65	8.49	−403	−486 ± 72
$\phi$	1019.46	4.26	−213	−135 ± 59

Let us consider the narrow resonances $\omega$ and $\phi$ which are well parameterized by the Breit–Wigner (BW) formula:

149 $$\begin{aligned} \sigma _\mathrm{BW}(s)=\frac{12\pi \Gamma _e\Gamma _f}{M_R^2}\frac{s}{(s-M_R^2)^2+M_R^2\Gamma _R^2}, \end{aligned}$$

which has its maximum at $\sqrt{s}=M_R\,(1+\gamma ^2)^{\frac{1}{4}}$ with $\gamma =\Gamma _R/M_R$. The peak value is $\sigma _{max}=\frac{12\pi \Gamma _e\Gamma _f}{M_R^2\Gamma _R^2}\,(1+\frac{1}{4} \gamma ^2)$ and the half maximum locations read $(\sqrt{s})_\pm =M_R\,(1+\frac{3}{8} \gamma ^2)\pm \frac{\Gamma _R}{2}\,(1-\frac{1}{8} \gamma ^2)$. The leading photon radiation modifies these resonance parameters to

150 $$\begin{aligned} \sqrt{s}_{\mathrm{max}}= & {} M_R+\frac{\beta \pi \Gamma _R}{8}-\frac{1}{4} \gamma ^2\,M_R, \end{aligned}$$

151 $$\begin{aligned} \rho= & {} \frac{\sigma ^{\mathrm{obs}}}{\sigma _{\mathrm{BW}}}=\left( \frac{\Gamma _R}{M_R}\right) ^\beta \,(1+\delta _1^{v+s}), \end{aligned}$$

152 $$\begin{aligned} \sqrt{s}_+-\sqrt{s}_-= & {} \Gamma _R\, \left[ 1-\frac{\pi }{2}\beta \gamma -\frac{5}{8} \gamma ^2\right. \nonumber \\&\left. +\left( \frac{\pi }{4}\beta +\frac{\beta }{2}\ln 2\right) \left( 1+\beta +\frac{\pi }{4}\beta \right) \right] . \end{aligned}$$

The physical resonance appears wider, with a reduced peak cross section, and the position of the observed peak is shifted towards higher energies. Considering the emission of a second photon, $s'=s(1-k)$ in the convolution formula (146) turns into $s''=s'(1-k_2)$, where $s'=s(1-k_1)\sim M_R^2$, i.e. $s=M_R^2$ in the relations listed before. This second photon shifts the peak energy of the $\omega$ and $\phi$ by $\delta E$ as given in Table 14.

The last data column here displays, correspondingly, the values for the shifts $\delta E^{\omega }_{BESIII}$ and $\delta E^{\phi }_{BESIII}$ returned by a global $\hbox {EBHLS}_2$ fit involving all data and, especially for the 3$\pi$ channel, both the NSK and the BESIII samples; these fit values differ from the corresponding calculated ones by only $1\sigma$.

Interestingly, the calculated leading order shifts quite effectively reproduce the shifts found by optimizing the fits of the BESIII data (about a $1\sigma$ distance only for both signals). Therefore, the question is whether BESIII has included (or not) the corresponding corrections when unfolding the raw data from radiation effects.

## Footnotes

1 One should remember that $\tau$-based evaluations of the muon HVP may not be in contradiction with the BMW estimate, as can be seen in the recent [5] (see also [2] for previous evaluations). This, however, supposes that the significant isospin breaking mechanisms involved in the pion form factor in the $\tau$ decay and in $e^+e^-$ annihilations – which can well differ – have all been clearly identified. This is one of the issues addressed in this article.

2 See [13] for a comprehensive review covering the anomalous and non-anomalous sectors.

3 To avoid lengthy repetitions, we refer the reader to the companion paper [19] for details. The present study is essentially an extension of $\hbox {BHLS}_2$ which endorses its content, particularly its vector sector.

4 As one assumes the FKTUY [12] intrinsic parameters $c_3$ and $c_4$ to be equal, *VAP* couplings (*A* being the electromagnetic field) identically vanish.

5 Under conditions on $\delta V$ discussed in Sects. 4 and 7 of [19].

6 For instance, the coefficients of the subtraction polynomial of any given loop function may undergo significant correlations in minimization procedures; they are accounted for in the parameter error covariance matrix returned by the fits.

7 Note the missing square root symbol in the definition for $X_V$ given in [19].

8 See also Sect. 4.3 in [14].

9 The corresponding fits have been performed fixing $\theta _P$ through the condition $\theta _0\equiv 0$ and imposing the condition $\epsilon \epsilon ^\prime =-\epsilon _0^2 \sin {2 \theta _P}$, as recalled in Sect. 2; one yields now more favorable $\chi ^2$ by having released these constraints, as will be seen shortly.

10 This issue was already addressed in [41] in the context of an oversimplified version of the broken HLS model.

11 In the present paper, we focus solely on the BS variant – which carries three fewer floating parameters than the RS variant – as RS and BS return similar pictures with the collection of existing samples and, especially, in the $\tau$ sector.

12 See also Sect. 9 below.

13 All breaking parameters occurring in $\hbox {BHLS}_2$, in particular those associated with the breaking of the isospin symmetry or of the nonet symmetry, are considered as being of order $\delta$, a generic perturbation parameter. All expressions derived from our Lagrangian are understood to be truncated at this order and, therefore, terms of order $\mathcal{O}(\delta ^2)$ or higher are always discarded.

14 The derivation of $\hbox {EBHLS}_2$ is mainly addressed in Sects. 4 and 6.

15 One should remember that, in contrast to the neutral vector current, the charged vector current is conserved only in the isospin symmetry limit.

16 In the broken HLS frameworks, the pion form factor fulfills $F^e_\pi (0)= 1+ \mathcal{O} (\delta ^2)$; when $\lambda _3$ is left free, the fit returns a departure from 1 by 2 permil, whereas it becomes 1.3% when $\lambda _3$ is fixed to zero. This is the origin of the “shift” exhibited by the two curves shown in Fig. 1.

17 The recent [59] develops such a kinetic breaking focusing on a treatment of the $\eta -{\eta ^\prime }$ system. Previous studies, like [60, 61] for instance, already considered such a breaking mechanism.

18 Regarding the vector meson sector, the layout of the full renormalization procedure has already been defined and thoroughly described in [19]; it applies here without any modification and will not be rediscussed.

19 We have already heuristically defined the first non-leading order in perturbative expansions by some generic (perturbation) $\delta$ parameter; this notation is preferred to the previous naming $\epsilon$ used in our [19] to avoid confusion with breaking parameters to be introduced below.

20 In the literature, $\lambda$ is named $\Lambda _1$ [32, 33, 37]. Removing the derivative symbols in Eq. (17) generates a singlet mass term – the topological susceptibility – to account for the ${\eta ^\prime }$ mass.

21 Assuming $\lambda$ to be positive, which is supported by our former fit results [14, 19].

22 As stated in Sect. 2, the breaking parameter $\Sigma _V$ is phenomenologically out of reach, and we impose $\Sigma _V \equiv 0$ within our fits; it is, nevertheless, kept in the model expressions for information.

23 One may note that $F_2(s)$ in Eq. (144) corrects for an error in [19] missed in the Erratum ($3\xi _3/2 \rightarrow 2 \xi _3$).

24 Actually, the 3$\pi$ channel is marginally sensitive to the differences between $\hbox {BHLS}_2$ and its extension to $\hbox {EBHLS}_2$.

25 In the process of sample combination frameworks, additional issues may arise; for instance, the consistency of the absolute energy calibration of the various experiments with each other should be addressed as done in [19] with the dikaon data samples collected by CMD3 [71, 72] and BaBar [40].

26 Numerically, $\mu$ is derived using the function values *M*(*s*) at the central values of the fit parameters.

27 We have preferred skipping the first few data points more subject to non-negligible background; the spectrum is thus fitted in the energy range $\sqrt{s} \in [0.73 \div 1.05]$ GeV.

28 For short, these will be referred to below as NSK.

29 Thus, the normalization correction applied to each of the NSK 3$\pi$ data samples is determined by the fit of solely the BESIII data [24] within the global framework.

30 Our reference energy is actually defined consistently by more than 50 data samples. As an important part of these have been collected at the VEPP-2M Collider in Novosibirsk, we denote, when needed, our reference energy by $E_\mathrm{NSK}$.

31 Note that, for the NSK scan experiments, photon emission on resonances is corrected locally resonance by resonance.

32 Of course, it is unrealistic to expect that the spectrum and uncertainties defining any data sample have been *perfectly* determined – see below.

33 The CMD3 data have, nevertheless, been dealt with to estimate systematics in the muon HVP evaluation [19].

34 KLOE indicates the simultaneous use of KLOE10 and KLOE12

35 One should note that the estimates for $a_\mu (\pi ^+\pi ^-, \sqrt{s}\le 1.05)~ \mathrm{GeV}$ differ marginally: $493.18 \pm 0.90$ ($\mathcal{H}_R$ + NSK + KLOE) and $493.75 \pm 0.79$ ($\mathcal{H}_R$ + NSK + KLOE85) in units of $10^{-10}$.

36 There is no explicit statement in [25] about how the systematics should be understood; however, this assumption corresponds to what is usually understood with the data collected at the Novosibirsk facilities. This topic is further discussed just below.

37 For convenience, here, the $3\pi$ annihilation channel and data are discarded from the fit procedure.

38 The pion form factor squared table in [25] gives numbers with only one decimal digit, so adding 0.04 to the statistical uncertainty rounds down to 0.

39 It has been found appropriate to reintroduce the spacelike data from [54, 55] within the minimization procedure. As the pion form factor in the spacelike and timelike regions is the same analytic function, this is a constraint.

40 Actually, this appears to be the natural way, if not the only one, to reliably and consistently derive the correction to the normalization of the various spectra.

41 See below the sections devoted to the $[\pi ^0,\eta ,{\eta ^\prime }]$ mixing.

42 This means that $\hbox {BHLS}_2$ and $\hbox {EBHLS}_2$ actually carry an identical parameter freedom.

43 Our [15] provided a study of $\phi$ mass region in the BaBar spectrum.

44 A part of this effect might already be accounted for in the experimental uncertainties.

45 In the HLS region ($\sqrt{s} < 1.05$ GeV), we have the FSR contributions $4.26 \times 10^{-10}$ from $\pi ^+\pi ^-\gamma$, $0.17 \times 10^{-10}$ from $\pi ^+\pi ^-\pi ^0\gamma$ and $0.38 \times 10^{-10}$ from $K^+K^-\gamma$. Altogether this amounts to $4.81 \times 10^{-10}$, to which a 2% uncertainty is attributed, i.e. $0.10 \times 10^{-10}$.

46 A model uncertainty, estimated to $\pm 0.3 \times 10^{-10}$ in Sect. 19, has been added linearly to the systematics.

47 It is provided by the low-energy tails of channels like $e^+ e^- \rightarrow 4 \pi$, $2\pi \eta$ or ${\eta ^\prime }\gamma \cdots$, the thresholds of which are smaller than the $\phi$ meson mass.

48 Using only the BNL datum leads to a significance for the $\Delta a_\mu$ central value of $4.7 \sigma$.

49 The three-pion data are discarded from the fits reported in this Table.

50 Including the former data collected in [76].

51 Actually, among the data traditionally used to address this topic, only the $J/\psi$ decay information remains outside the $\hbox {EBHLS}_2$ scope.

52 Let us remember that the $\lambda$ parameters introduced via the generalized ’t Hooft term are treated as $\mathcal{O}(\delta ^{1/2})$

53 In our previous papers as well as below – see Eq. (112) – we preferred defining the ideal mixing angle by $\theta _I=\arctan {1/\sqrt{2}}\simeq 35^\circ$ in more natural correspondence with the $\rho ^0-\omega$ ideal mixing angle.

54 See Eqs. (28–30) in [37].

55 It happens that this $f_s$ differs from the $F_s$ defined in the preceding section; they are related by $f_s f_\pi =F_s^2$.

56 The condition $\lambda _q=0$ implies that $\lambda _0$ and $\lambda _8$ are either simultaneously nonvanishing or simultaneously vanishing; we show just below that $\lambda _q=0 \Longrightarrow \lambda _3=0$.

57 Actually, each of the solutions below is twofold degenerated; indeed, as each physical quantity exhibits a dependence only upon squares of the $\lambda _i$, any solution $\{\lambda _i=\lambda _i^0, i=0,3,8 \}$ carries the same physics as its twin $\{\lambda _i=-\lambda _i^0, i=0,3,8 \}$.

58 This comes down in dropping out the products $\lambda _i \lambda _j$ for $i\ne j \in (0,3,8)$ in all the expressions given in the sections above and in the appendices.

59 Another formulation [39] in terms of the quark mass difference and of the mixing angle named here $\theta _P$ is noted below; it has been used in our previous studies [14, 15, 19, 75] and its fit properties will be discussed below.

60 In this expression, the subtracted electromagnetic contribution to kaon mass difference is estimated as $\Delta M_K = \Delta _\pi = M_{\pi ^0}^2-M_{\pi ^\pm }^2 =-1.24\times 10^{-3}$ $\hbox {GeV}^2$. However, as discussed in [38], its exact magnitude is rather controversial; for instance, Moussallam [111] rather yields $\Delta M_K=k \Delta _\pi$ with $k=2 \div 3)$.

61 The $\pi ^0 VP$ and $\eta VP$ are hidden inside the $\pi ^0 \gamma$ and $\eta \gamma$ annihilation cross sections and are of comparable quality in both solution $A_\pm$ fits.

62 In our fits, $c_3=c_4$ is imposed [14, 19].

63 A closer look at the various channel contributions indicates that this excess comes entirely from the anomalous channels, in particular from the $3 \pi$ annihilation, which then contributes $[46.30\pm 0.36]\times 10^{-10}$, whereas the solution reported in Table 7 only yields $[44.22 \pm 0.32] \times 10^{-10}$.

64 The ${\eta ^\prime }V \gamma$ couplings are explicitly involved in the data of the $\hbox {EBHLS}_2$ bunch; the $\eta V \gamma$ couplings are treated as part of the $e^+ e^- \rightarrow \eta \gamma$ annihilation cross sections.

65 We know of no update of this old result.

66 A kinetic breaking effect going beyond the usual ’t Hooft determinant term – which only provides a correction to the singlet term – has been proposed by other authors using different Lagrangians, see [59] for instance.

67 It is shown that this results in mere energy shifts, albeit different between the $\omega$ and $\phi$ peak regions; both values are found consistent with BESIII expectations. A possible origin for this difference is discussed in Appendix D.

68 The $0.60\div 0.71$ GeV region of the BaBar spectrum has been eliminated from the fit to stay close to what SND suggests [25], whereas no cut has been applied in the global fit involving the KLOE spectra; this obviously enhances the probability of the (SND20+Babar) combination.

69 In case the kinetic breaking term given by Eq. (21) is replaced by Eq. (22), the Kroll conditions yield a unique nontrivial solution.
